# Analysis and tuning of hierarchical topic models based on Renyi entropy approach

**DOI:** 10.7717/peerj-cs.608

**Published:** 2021-07-29

**Authors:** Sergei Koltcov, Vera Ignatenko, Maxim Terpilovskii, Paolo Rosso

**Affiliations:** 1 Laboratory for Social and Cognitive Informatics, National Research University Higher School of Economics, St. Petersburg, Russia; 2 Pattern Recognition and Human Language Technology Research Center, Universitat Politècnica de València, Valencia, Spain

**Keywords:** Topic modeling, Renyi entropy, Hierarchical topic models, Optimal number of topics

## Abstract

Hierarchical topic modeling is a potentially powerful instrument for determining topical structures of text collections that additionally allows constructing a hierarchy representing the levels of topic abstractness. However, parameter optimization in hierarchical models, which includes finding an appropriate number of topics at each level of hierarchy, remains a challenging task. In this paper, we propose an approach based on Renyi entropy as a partial solution to the above problem. First, we introduce a Renyi entropy-based metric of quality for hierarchical models. Second, we propose a practical approach to obtaining the “correct” number of topics in hierarchical topic models and show how model hyperparameters should be tuned for that purpose. We test this approach on the datasets with the known number of topics, as determined by the human mark-up, three of these datasets being in the English language and one in Russian. In the numerical experiments, we consider three different hierarchical models: hierarchical latent Dirichlet allocation model (hLDA), hierarchical Pachinko allocation model (hPAM), and hierarchical additive regularization of topic models (hARTM). We demonstrate that the hLDA model possesses a significant level of instability and, moreover, the derived numbers of topics are far from the true numbers for the labeled datasets. For the hPAM model, the Renyi entropy approach allows determining only one level of the data structure. For hARTM model, the proposed approach allows us to estimate the number of topics for two levels of hierarchy.

## Introduction

The large flow of news generated by TV channels, electronic news sources and social media is very often represented as a hierarchical system. In such a system, news items or messages are divided into a number of global topics, such as politics, sports, or health. Within each of the main topics, the documents usually demonstrate a considerable diversity and can be divided into subtopics, such as COVID-19, healthcare availability or healthy food within the global topic of health. The hierarchical division of information content is highly convenient and seems to reflect fundamental cognitive features of humans (Cohen, 2000; Palmer, 1977; Taylor et al., 2015). Therefore, starting from 2004, active development of probabilistic topic models that allow for identifying hierarchical topical structures in large datasets has begun (Blei et al., 2003; Li & McCallum, 2006). However, such models have a set of parameters, which need to be tuned to obtain a topical solution of higher quality. Correspondingly, a problem of tuning hierarchical topic models arises. A solution to this problem is hindered by a number of factors. First, there are no generally accepted or appropriate metrics of quality that take into account the features of hierarchical modeling, namely, the relations between pairs of parent and child topics. Second, the number of publicly available datasets with the hierarchical mark-up, which can be used to tune and compare hierarchical models, is very limited. Third, when applying hierarchical topic models on real datasets, the type of topical structure of data (non-hierarchical against hierarchical, as well as the number of levels in the case of hierarchical structure) is usually not known in advance. Fourth, hierarchical topic models, just like flat topic models, possess a certain level of semantic instability, which means that different runs of the algorithm on the same source data lead to different solutions, which, in turn, correspond to different local maxima of the model posterior: the model may arrive to different local maxima depending on the randomness in initialization and sampling. An analysis and discussion of topic models instability can be found in work (Koltsov et al., 2016). This problem complicates the search for optimal model hyperparameters on a given dataset. Thus, investigation and assessment of the ability to tune hierarchical topic models is an important task.

In this work, we investigate the behavior of three hierarchical models, namely, hierarchical latent Dirichlet allocation (hLDA) (Blei et al., 2003), hierarchical Pachinko allocation (hPAM) (Mimno, Li & McCallum, 2007), and hierarchical additive regularization of topic models (hARTM) (Chirkova & Vorontsov, 2016), in terms of two metrics: log-likelihood and Renyi entropy. We conduct experiments on four marked-up collections, two of which are non-hierarchical and two others have two-level hierarchical mark-up. The latter means that assessors have assigned each news item to a lower-level topic which in turn has been assigned to a higher-level topic, and have also determined the overall number of topics at each level and the number of lower-level topics within each higher-level topic. The goal of our research is threefold. First, we aim to estimate the ability of hierarchical models to identify whether the data structure is hierarchical or non-hierarchical. Second, we seek to develop a metric of quality that is suitable for hierarchical models, notably for topic number search at different levels of hierarchy. Third, based on this metric, we aim to offer an approach for tuning model hyperparamters so as to find the true number of topics or a value close to it. For this, we propose an extension of our entropic approach (Koltcov, 2018), which was developed earlier for non-hierarchical topic models, as a partial solution to the hierarchical model tuning. The approach is based on the information theory, according to which the information maximum corresponds to the entropy minimum. This means that topic modeling solutions with minimal entropy are likely to be the most informative which indicates a potential utility of entropic approach for tuning all model hyperparameters. However, in this paper we focus on topic number optimization specifically.

To simplify the structure of this work, an overview of hierarchical models and existing metrics of quality is provided in Appendix A. Thus, our work consists of the following parts. ‘Entropic approach to hierarchical topic modeling’ describes the entropic approach for estimating the quality of topic models and introduces the adaptation of this approach to hierarchical models. ‘Computational experiments’ outlines the design of our computer experiments for each of the considered models. ‘Numerical results’ contains an analysis of the behavior of hierarchical models under variation of hyperparameters and the number of topics for the three models on four datasets. ‘Discussion’ interprets the obtained results and reviews the possible limitations of the proposed approach. ‘Conclusion’ summarizes our findings and contains practical recommendations for choosing between hierarchical models. Appendix B contains additional experiments with different types of pre-processing that investigate the influence of the latter on behavior of Renyi entropy.

## Entropic approach to hierarchical topic modeling

The entropic approach for topic models is based on the concept that an information system consisting of a large number of words and documents represents a statistical system. The state of such a statistical system can be characterized by the value of entropy. It is well known that the maximum entropy of a statistical system corresponds to either chaos or a uniform distribution of the system elements. However, unlike real physical systems, a text collection can be subject to procedures (for instance, clustering or topic modeling) that change the value of entropy by ordering the data. Based on the fact that entropy equals minus information *S* = − *I* (Beck, 2009), one can implement the process of document clustering or topic modeling in such a way that leads to the entropy minimum (information maximum), which corresponds to a highly non-equilibrium distribution. Since the procedure of clustering of text collections strongly depends on the values of model parameters, which include the number of topics as a parameter, an algorithm of model tuning can be organized in the form of searching for parameters that would lead to minimum entropy. Let us note that modern widely used topic models usually include no hyperparameters optimization algorithms (or include only for some of them). Thus, this is the user who has to select the values of model parameters based on some external metrics of quality.

Recent research (Koltcov, 2018; Koltcov et al., 2020) has demonstrated that the most convenient metric for estimating the state of a textual statistical system is Renyi entropy, whose calculation is entirely based on the words’ probabilities of belonging to a particular cluster or topic obtained in the process of topic modeling (matrix *Φ* = {*ϕ*_*wt*_}, where *ϕ*_*wt*_ is the probability of word *w* in topic *t*; for more details we refer the reader to Appendix A). Calculation of Renyi entropy for a topic model is based on two observable variables: (1) Gibbs-Shannon entropy of the model; (2) internal energy of the model. Gibbs-Shannon entropy *S* can be calculated as follows: }{}S = \ln (\rho ) = \ln ({N \over {WT}}), where *N* is the number of words with *ϕ*_*wt*_ > 1/*W*, *W* is the number of unique words in the dataset, *T* is the number of topics. The internal energy *E* of a topic model can be expressed in the following way: }{}E = - \ln (\tilde P) = - \ln \left( {{1 \over T}\sum\nolimits_{wt} ({\phi _{wt}} \cdot {1_{\{ {\phi _{wt}} > 1/W\} }})} \right), where \sum\nolimits_{wt} ({\phi _{wt}} \cdot {1_{\{ {\phi _{wt}} > 1/W\} }}) is the sum of probabilities of words, each of which is above the threshold 1/*W*. Then, Renyi entropy can be calculated through free energy (*F* = − *qE* + *S*) in the following way:

(1) }{}S_q^R = \displaystyle{F \over {q - 1}} = \displaystyle{{q\ln (\tilde P) + \ln (\rho )} \over {q - 1}},

where the deformation parameter *q* = 1/*T* is the inverse number of topics. Thus, Renyi entropy of a topic model explicitly contains ’number of topics’ as a deformation parameter. Application of Renyi entropy for tuning different non-hierarchical topic models is considered in work (Koltcov et al., 2020).

As shown in Blei et al. (2003) and Mimno, Li & McCallum (2007), the structure in hierarchical topic models can be represented as a graph, where each node at each level is a topic *t* of the corresponding degree of abstractness. While flat topic modeling results in obtaining one distribution of probabilities of all words over all topics (represented in matrix Φ), and one distribution of probabilities of all documents over all topics (represented in matrix Θ), hierarchical topic modeling constructs one pair of these matrices per each level of hierarchy (Φ_*i*_, Θ_*i*_, where *i* is the level of the hierarchy). The number of levels, and most often the number of nodes-topics per each level, is a value pre-selected by a user. In some models, topics of a lower level are united in clusters each of which is nested in only one topic of a higher level; in other models, hierarchy levels are unrelated and only differ by the number of topics, so that lower levels contain more topics. At all levels, matrices Φ_*i*_, Θ_*i*_ use the same number of words *W* and documents *D* which equals the total number of words and documents in the collection, respectively. However, the share of words with high probabilities (*i.e*., probabilities above the threshold) is the lower, the higher is the level of hierarchy.

As it can be seen, instead of a single topical solution, hierarchical topic modeling obtains a set of solutions whose number equals to the number of levels *L*. Each solution can be characterized by the following variables: (1) the number of topics *T*_*i*_ on level *i*; (2) the number of words (*N*_*i*_) with high probabilities: \phi _{wt}^i > 1/W; (3) the sum of probabilities above the threshold: }{}{P_i} = \sum\nolimits_{wt} \phi _{wt}^i \cdot {1_{\{ \phi _{wt}^i > 1/W\} }}. Based on these variables, one can calculate the internal energy *E*_*i*_ and Gibbs-Shannon entropy *S*_*i*_ of the the solution of level *i* with respect to the equilibrium state of that level: }{}{E_i} = - \ln ({{{P_i}} \over {{T_i}}}), }{}{S_i} = \ln ({{{N_i}} \over {W{T_i}}}). Using *S*_*i*_ and *E*_*i*_, one can determine free energy and Renyi entropy of level *i*. Free energy is expressed as follows: *F*_*i*_ = *E*_*i*_ − *T*_*i*_ · *S*_*i*_. Renyi entropy of level *i* can be expressed in the following way: }{}S_i^R = {{{F_i}} \over {1 - q}}, where *q* = 1/*T*_*i*_ is the deformation parameter characterizing each level of the hierarchy.

By measuring the value of entropy at each level while consecutively increasing the number of topics, it is possible to find a solution optimal in terms of information theory. In such a design, the process of clustering of words by topics starts with the minimum information (maximum entropy) when all the elements (words) of the statistical system are assigned to one or two topics and ends also with the maximum entropy when all the elements are almost uniformly distributed over topics (when the number of topics is large). The locations of the global minimum and of several possible local minima of Renyi entropy, as a function of the number of topics, are determined by the features of the data. Thus, Renyi entropy }{}S_i^R serves a measure of disequilibrium for a given system. By additionally varying other hyperparameters one can estimate how optimal each combination of them is.

## Results

### Data

In our numerical experiments, the following datasets were used:

- ‘Lenta’ dataset (from lenta.ru news agency, available at https://www.kaggle.com/yutkin/corpus-of-russian-news-articles-from-lenta). This dataset contains 8,630 documents with a vocabulary of 23,297 unique words in the Russian language. Each of these documents is manually assigned to a class from a set of 10 topic classes. Since some of the topics are closely related to each other, 7–10 topics can describe the documents of this dataset.
- ‘20 Newsgroups’ dataset (http://qwone.com/~jason/20Newsgroups/) is a widely used dataset in the field of topic modeling. It consists of 15,425 news articles in the English language with 50,965 unique words. Each of the news items is assigned to one of 20 topic groups. Since some of these topics might be combined, 14–20 topics can describe this dataset’s documents according to Basu, Davidson & Wagstaff (2008).
- ‘WoS’ dataset (available at https://data.mendeley.com/datasets/9rw3vkcfy4/1) is a dataset in the English language with a two-level hierarchical mark-up. The original dataset contains 46,985 abstracts of published papers available from the *Web of Science*. The first level contains seven categories (domains): computer science, electrical engineering, psychology, mechanical engineering, civil engineering, medical science, and biochemistry. The second level is comrpised of 134 specific topics (areas), each of which belongs to one of the categories of the first level. The number of unique words is 80,337. This dataset is often used as a benchmark for hierarchical classification (Sinha et al., 2018). However, this dataset exhibits a highly unbalanced distribution of the number of documents per sub-category. For instance, some sub-categories contain more than 700 documents, while others are represented by less than 50 documents. Therefore, we also consider a balanced subset of this dataset (described below), where poorly presented topics, *i.e*., topics with a small number of documents, were deleted.
- ‘Balanced WoS’ dataset (available at https://data.mendeley.com/datasets/9rw3vkcfy4/1) is a class-balanced subset of the ‘WoS’ dataset, which contains 11,967 abstracts. The first level contains seven categories and the second level consists of 33 areas.
- ‘Amazon’ dataset (available at https://www.kaggle.com/kashnitsky/hierarchical-text-classification/version/1) is a dataset with a three-level hierarchical mark-up. It contains 40,000 product reviews in English from *Amazon*. The vocabulary of this dataset consists of 31,486 unique words. Level 1 of the hierarchical mark-up contains six categories, level 2 contains 64 categories, and level 3 contains 510 categories. We consider only the first two levels since the third level contains ‘unknown’ labels. Let us note that the original dataset is highly imbalanced. Some sub-categories contain less than 50 documents, while other sub-categories contain more than 2,000 documents. Therefore, a balanced subset of this dataset is also considered.
- ‘Balanced Amazon’ dataset is a subset of the ‘Amazon’ dataset that includes only sub-categories with the number of documents above 500. As a result, level 1 contains six categories and level 2 contains 27 sub-categories. The total number of documents is 32,774, and the number of unique words is 28,422.

Statistical features of the above datasets are summarized in Table 1.

**Table 1:** Statistics of the datasets.

Dataset	Number of documents	Vocabulary size	Labeling	Number of topics	
				*T*_1_	*T*_2_
Lenta	8,630	23,297	Non-hierarchical	10	
20 Newsgroups	15,425	50,965	Non-hierarchical	20	
WoS	46,985	80,337	Hierarchical	7	134
Bal. WoS	11,967	36,488	Hierarchical	7	33
Amazon	40,000	31,486	Hierarchical	6	64
Bal. Amazon	32,774	28,422	Hierarchical	6	27

### Computational experiments

In our numerical experiments, we used implementations of hPAM and hLDA models from *tomotopy* package, version 0.9.1 (https://bab2min.github.io/tomotopy/v0.9.1/en/), and the implementation of hARTM model from *BigARTM* package, version 0.10.1 (https://bigartm.readthedocs.io/en/stable/). With each of them, we performed topic modeling on both types of our datasets—those with the hierarchical and the flat mark-up. Correspondingly, the results are divided into two parts. In the first part, we analyze the applicability of the models for the datasets with flat structure, while focusing on the datasets with hierarchical mark-up in the second part. Since topic modeling possesses a certain level of instability that leads to fluctuations in the word probabilities, all calculations were performed at least six times (for each combination of hyperparameters), and then the results were averaged. For each model we calculated Renyi entropy, perplexity and log-likelihood, the two latter having been chosen as the most common metrics for hyperparameters tuning in topic modeling. However, as perplexity is in fact a reciprocal geometric mean of log-likelihood (see Appendix A), its behavior has turned out to be nearly identical to the latter. Therefore, we do not report it in our analysis. In this paper we also leave out metrics related to semantic coherence or semantic stability, such as standard topic coherence (Mimno et al., 2011), tf-idf coherence (Nikolenko, Koltcov & Koltsova, 2017), Jaccard index or their extensions, since their use needs significant adaptation to our task and therefore deserves a separate investigation. Thus, while these metrics, after an adaptation, can be used independently, a more promising avenue is to include them as an entropy parameter into two-parametric Sharma–Mittal entropy, as is shown in Koltcov, Ignatenko & Koltsova (2019). However, this is beyond the scope of this paper.

After entropy and log-likelihood is calculated for a sequence of solutions with varying number of topics, we plot the values of these metrics as functions of the number of topics and investigate whether the minima of these graphs, if any, fall on or near the value of the number of topics suggested by human markup. As we do not infer the methods of calculation of either entropy or log-likelihood from the data, we do not divide our collections into training and test sets, and both metrics are calculated on the entire datasets. In an additional set of experiments described in Appendix B we evaluate the influence of three different types of text preprocessing on the location of the minimum of entropy as a function of the number of topics.

Below we describe specific features of the main experiments for each of the three tested algorithms.

#### hPAM model

The hPAM model depends on the following parameters: (1) number of topics at the second and the third levels; (2) hyperparameter *η*; (3) hyperparameter *α*. The number of topics at the first level of hPAM model is always set to one. Moreover, in this model, the user can only set the initial value of parameter *α*, and then the algorithm tunes it during the modeling. Our experiments demonstrated that variation of the initial value of *α* (*α*_0_) does not influence the results of modeling, namely, different values of *α*_0_ lead to almost the same topic solutions and the same final value of *α*. Therefore, in the rest of the paper, we fix *α*_0_ to 0.0001. For a more detailed description of the model, we refer the reader to Appendix A.

In our work, hPAM model was studied in two stages. First, we investigated the second hierarchical level in the following way: the number of topics on the third level was fixed *T*_2_ = 1 while hyperparameter *η* was varied in the range (0.001, 1) and the number of topics on the second level (*T*_1_) was varied in the range (2, 200). For each solution, we calculated Renyi entropy and log-likelihood of the second level of the hierarchy. Then, we found and fixed several pairs of the values of *T*_1_ and *η* that were the closest to minimum Renyi entropy. Second, we investigated the third hierarchical level, where the number of topics *T*_2_ on the third level was varied under condition of a fixed pair of *T*_1_ and *η*. For each combination of *T*_2_ and the pair (*T*_1_; *η*), Renyi entropy of the third level was calculated.

#### hLDA model

The hLDA model has the following parameters: (1) depth of the hierarchy; (2) hyperparameter *α*, which is tuned by the model automatically; (3) hyperparameter *γ*; (4) hyperparameter *η*. hLDA model is non-parametric; therefore, it infers the number of topics on each level automatically.

In this work, we studied the dependence of the number of inferred topics on the parameter *η*, which was varied in the range (0.001,1). The depth of the hierarchy was set to three as in the experiments in the original work (Blei et al., 2003). The influence of parameter *γ* was not investigated in this work. Since this model is highly unstable and can produce different numbers of topics for the runs with the same values of hyperparameters, we ran the model 10 times for each value of *η*. Then, we estimated the range of the derived numbers of topics on the second and the third levels. The mean values of Renyi entropy and log-likelihood were calculated for each level.

#### hARTM model

The hARTM model has the following parameters: (1) number of topics at each level of the hierarchy; (2) seed—a parameter describing the initialization procedure (it defines the work of the random number generator). This model was also studied in two stages. First, the number of topics on the second level was fixed (*T*_2_ = 1), and the number of topics on the first level was varied in the range of (2, 200) topics. Based on the minimum Renyi entropy location, the optimal *T*_1_ was chosen. Second, the number of topics on the second level was varied under the condition of fixed *T*_1_. For each run of the model, the parameter seed was randomly selected to investigate the variability of the model output.

### Numerical results

#### hPAM model (investigation of the second hierarchical level)

**Figure 1:**
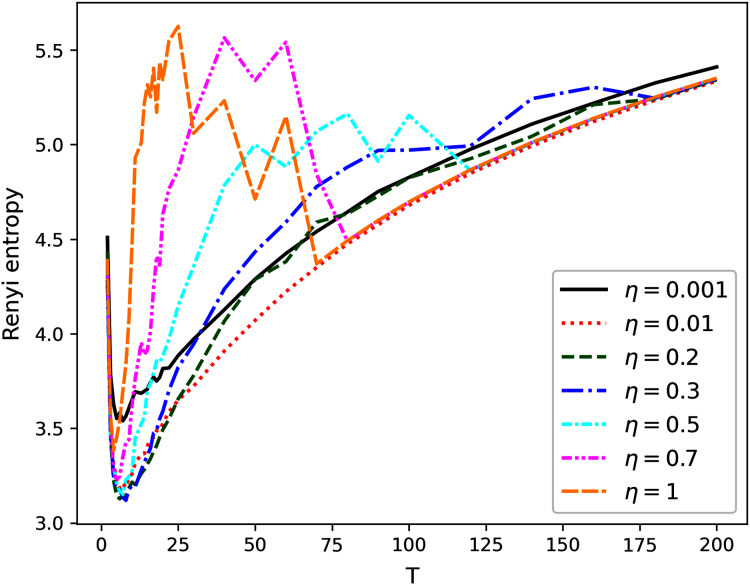
Renyi entropy curves (hPAM). Lenta dataset.

**Figure 2:**
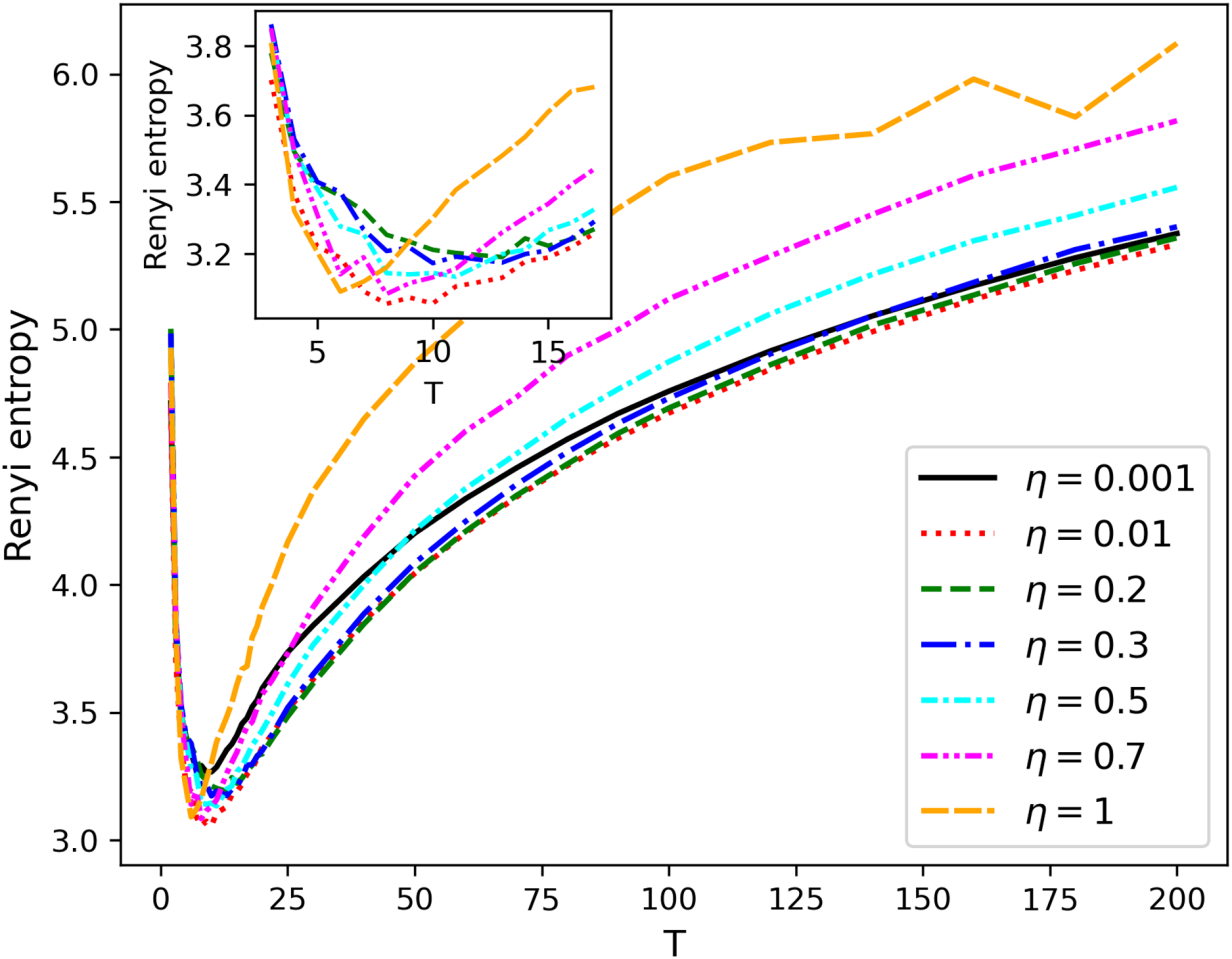
Renyi entropy curves (hPAM). 20 Newsgroups dataset.

**Figure 3:**
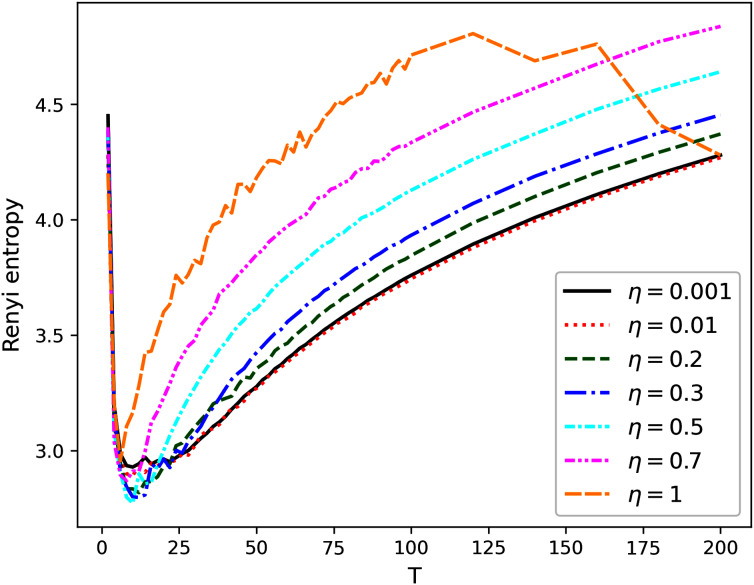
Renyi entropy curves (hPAM). Balanced WoS dataset.

**Figure 4:**
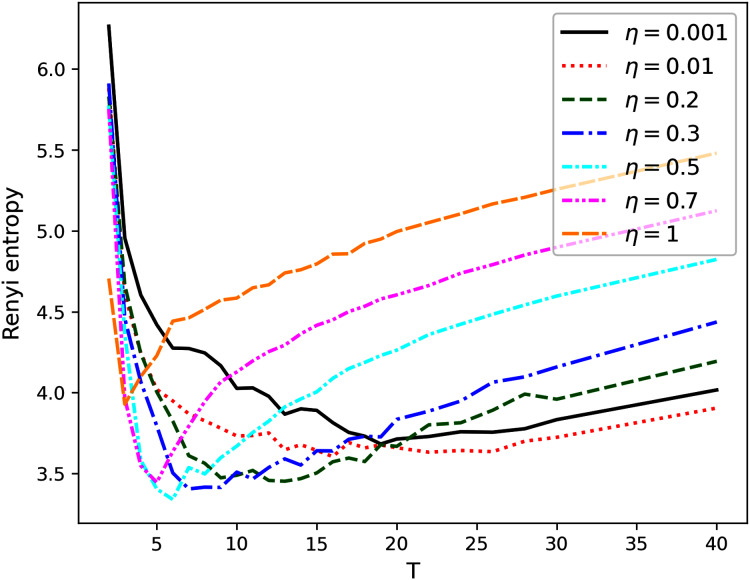
Renyi entropy curves (hPAM). Balanced Amazon dataset.

Figures 1–4 contain the results of the first stage of our test for hPAM model. They demonstrate the behavior of Renyi entropy under variation of *η* and the number of topics on the second level of hierarchy for different datasets. A similar pattern of Renyi entropy behavior for all datasets is observed in the ranges of small (about 2–3) and large (about 100–200) numbers of topics. These ranges correspond to the two extreme states of the statistical system characterized by entropy maximum. Moreover, one can see that the location and the value of minimum Renyi entropy significantly depend on the parameter *η*. Large values of *η* (*η* > 0.7) lead to significant fluctuations in the Renyi entropy for large numbers of topics that complicates finding entropy minimum the more, the higher the number of topics is. Correspondingly, the further increase of *η* is inadvisable.

The behavior of Renyi entropy for this model allows us to find approximations of the optimal number of topics on the second level for different datasets and the optimal value of *η* by means of selecting the values that correspond to the minimum entropy. Since we test the model on the datasets with human mark-up, we can estimate the error of the found approximation of the number of topics. For non-hierarchical datasets (Lenta and 20 Newsgroups), the error corresponds to *±*2 topics (Figs. 1 and 2). For hierarchical datasets we obtain the error of *±*4 topics (Figs. 3 and 4).

The list of values of *η* and corresponding numbers of topics with the values of average minimum Renyi entropy on the second hierarchical level is given in Table 2 (*T*_1_ refers to the number of topics on the second level of the hierarchy). Potentially interesting combinations of parameters that were used in our calculations at the second stage are highlighted in bold.

**Table 2:** Minimum points of Renyi entropy for hPAM model. Potentially interesting combinations of parameters that were used in our calculations at the second stage are highlighted in bold.

*η*	Lenta		20 Newsgroups		Bal. WoS		WoS		Bal. Amazon		Amazon	
	min.	*T*_1_	min.	*T*_1_	min.	*T*_1_	min.	*T*_1_	min.	*T*_1_	min	*T*_1_
0.001	3.54	7	3.27	9	3.66	8	3.25	8	3.68	19	3.93	14
0.01	3.18	6	**3.06**	**10**	2.89	10	2.96	9	3.6	16	3.74	15
0.2	**3.13**	**6**	3.19	13	2.82	12	**2.67**	**14**	3.45	13	3.47	12
0.3	**3.12**	**8**	3.17	10	**2.79**	**12**	**2.68**	**8**	**3.4**	**7**	**3.39**	**7**
0.5	**3.15**	**7**	3.14	9	**2.77**	**10**	**2.66**	**9**	**3.34**	**6**	**3.29**	**6**
0.7	3.23	5	**3.08**	**8**	2.87	8	2.95	5	3.44	5	4.01	3
1	3.38	4	3.09	6	2.96	6	3.39	6	3.93	3	3.93	3

**Figure 5:**
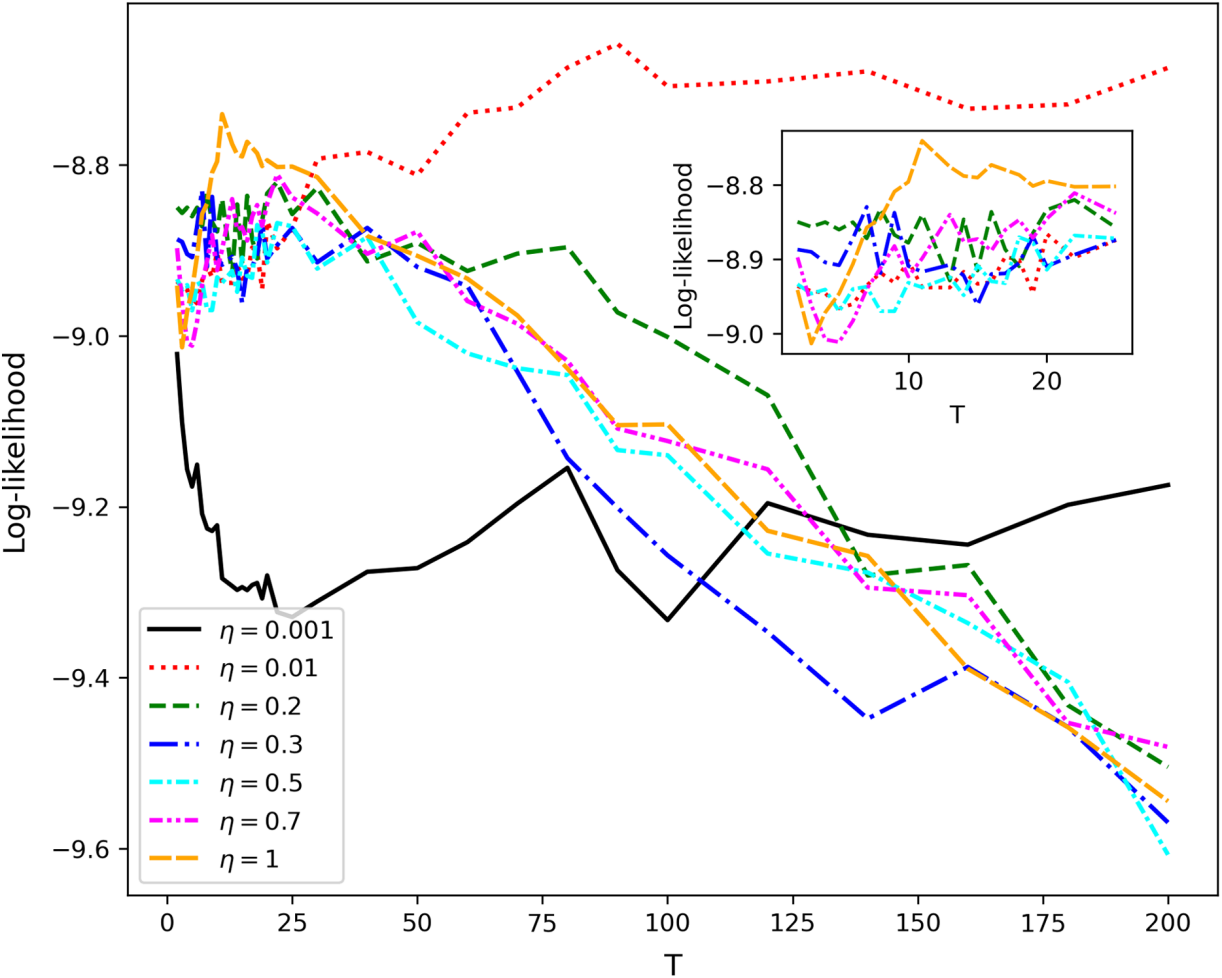
Log-likelihood curves (hPAM). Lenta dataset.

**Figure 6:**
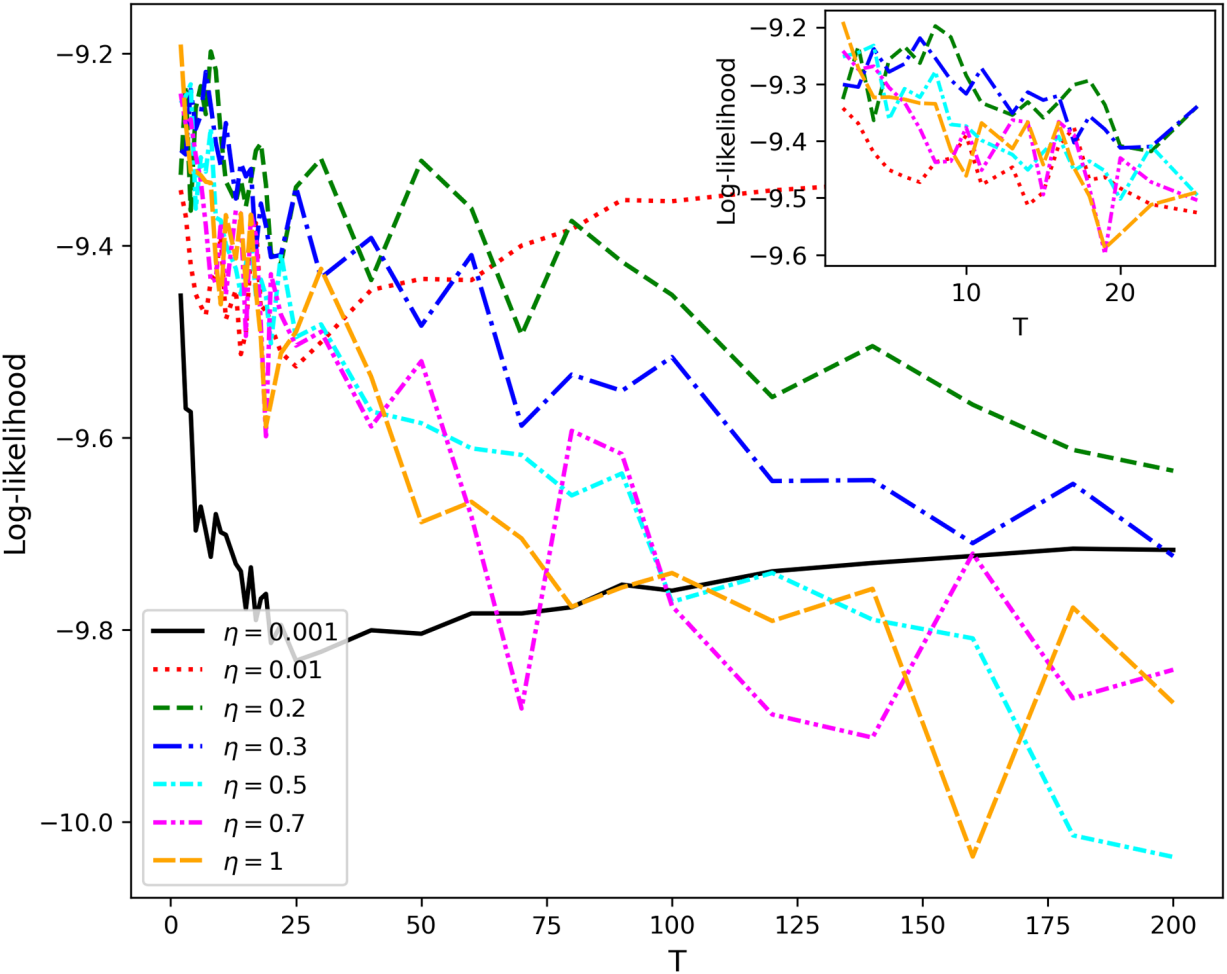
Log-likelihood curves (hPAM). 20 Newsgroups dataset.

Calculation of log-likelihood under variation of *T*_1_ and *η* demonstrates that this metric is not useful for selecting the optimal values of *η* and *T*_1_ since it has large fluctuations in the entire range of *T*_1_ (Figs. 5 and 6).

#### hPAM model (investigation of the third hierarchical level)

**Figure 7:**
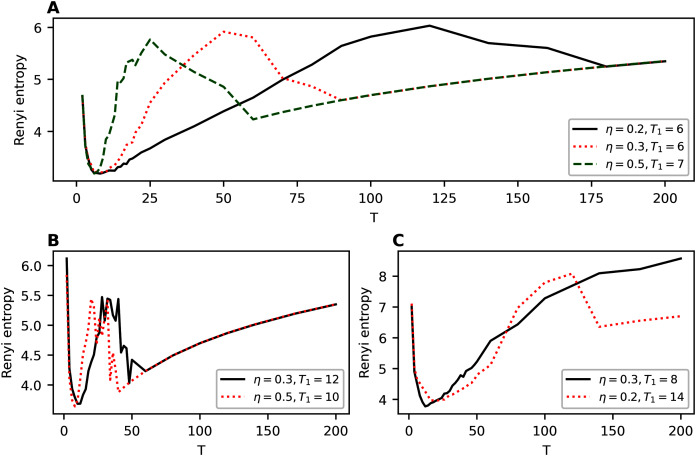
Renyi entropy curves (hPAM). (A) Lenta dataset. (B) Balanced WoS dataset. (C) WoS dataset.

Figure 7 demonstrates the behavior of Renyi entropy under variation of *T*_2_ for *T*_1_ and *η* chosen during investigation of the second hierarchical level. For almost all datasets except 20 Newsgroups, Renyi entropy curves have significant fluctuations and spikes. It should be noted that such fluctuations are typical for both balanced and unbalanced datasets. Moreover, parameter *η* visibly influences the location of a spike. Therefore, the estimation of the number of topics on the third level is much less accurate than that on the second level. Our calculations demonstrate that in the region of large fluctuations the model deteriorates. This means that the number of words with high probabilities becomes constant and does not change with the growth of the number of topics. Correspondingly, the sum of high probabilities also becomes constant, *i.e*., statistical features of obtained solutions do not change. In this case, Renyi entropy changes only because the number of topics changes while other variables are constant. Thus, due to the features of hPAM model, the selection of the number of topics on the third level is complicated. Moreover, the dependence of log-likelihood on *T*_2_ has similar behavior as for the second hierarchical level and, thus, does not allow us either to choose the right number of topics for hPAM model or to determine whether the dataset has a hierarchical or flat structure.

#### hLDA

Our calculations demonstrate that hLDA model cannot be used in real applications since it infers very different numbers of topics for different runs with the same parameters. Moreover, the inferred numbers of topics are far away from the true number for considered datasets. In addition, the order of dispersion and the mean value of the predicted number of topics on each level significantly depends on the value of parameter *η*. Due to high instability for *η* < 0.3 and incorrect output for *η* > 0.3, there is no sense in applying Renyi entropy approach to this model. Finally, log-likelihood metric does not allow us to choose the right number of topics either. The results of our calculations for hLDA model on different datasets are summarized in Table 3. Table 3 demonstrates the range of the derived number of topics for 10 runs on each dataset and for each value of hyperparameter *η*.

**Table 3:** Range of the derived number of topics by hLDA model for the second (T1) and the third hierarchical levels (T2).

*η*	Lenta		20 Newsgroups		Balanced WoS		Balanced Amazon	
	*T*_1_	*T*_2_	*T*_1_	*T*_2_	*T*_1_	*T*_2_	*T*_1_	*T*_2_
0.001	6–11	31–67	288–358	911–1,402	482–652	1,751–2,242	108–148	561–654
0.01	6–11	13–30	81–111	274–334	68–93	325–453	23–36	108–122
0.2	2–3	5–7	6–11	14–18	2–5	6–13	3	5–6
0.3	2	2–4	4–9	7–11	2–3	3–7	2–3	3–4
0.5	2	2–3	3–5	5–9	2	2–3	2–3	3–4
0.7	2	2–3	3–4	3–7	2	2–3	2	2–4
1	3	2–3	2–4	3–6	2	2–3	2	2–3

#### hARTM

**Figure 8:**
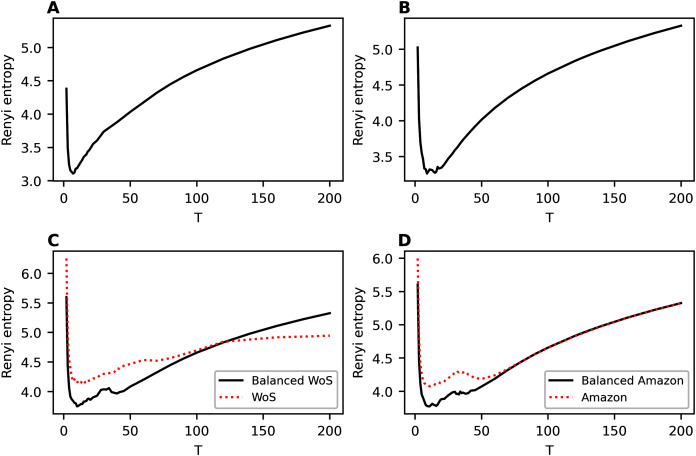
Renyi entropy curves (hARTM). (A) Lenta dataset. (B) 20 Newsgroups dataset. (C) Balanced WoS dataset and WoS dataset. (D) Balanced Amazon dataset and Amazon dataset.

Figure 8 demonstrates the behavior of Renyi entropy on the first level of hierarchy obtained with hARTM algorithm for different datasets. For non-hierarchical datasets, we clearly observe only one minimum of Renyi entropy. Moreover, the location of this minimum is close to the human mark-up, namely seven topics for Lenta dataset and 14 topics for 20 Newsgroups dataset. The behavior of Renyi entropy on the second level of hierarchy is almost identical to that of the first level; therefore, we do not provide figures. Let us note that Renyi entropy curve for hARTM model does not have sharp jumps compared to the hPAM model. Thus, the entropy approach can be successfully used for determining the structure of non-hierarchical datasets modeled with hARTM.

For hierarchical datasets, we observe two minima of Renyi entropy which allows us to identify two levels in the data structure. The first (global) minimum of Renyi entropy approximately corresponds to the first level of the hierarchical mark-up of the dataset and the second minimum corresponds to the second level. For instance, Renyi entropy for the balanced WoS dataset has a global minimum at the value of 10 topics, while human mark-up suggests seven topics. The second (local) minimum of Renyi entropy corresponds to 36–42 topics, and the mark-up discerns 34 topics. For the balanced Amazon dataset, the first minimum of Renyi entropy corresponds to 10 topics, with 6 topics on the first level provided by the mark-up. The second minimum is found at 38 topics, whereas the mark-up indicates 27 topics on the second level. Thus, the estimation of the number of topics on the second level has a larger error compared to that of the first level. However, to the best of our knowledge, Renyi entropy approach provides the best accuracy of estimating the number of topics for this hierarchical model. Moreover, our approach allows us to determine the very presence of hierarchical structure in the data. Let us note that balancing the datasets allows improving the results of topic modeling in terms of topic salience, making them strongly pronounced. This is achieved by obtaining higher probabilities of the most probable words (that is, by obtaining more skewed distributions of word probabilities within a topic). This, in turn, leads to the formation of a more pronounced entropy minimum and, on average, to lower values of entropy across the runs of the algorithm with different parameters. Figures 8C and 8D demonstrate the difference between Renyi entropy for the balanced and unbalanced datasets. The effect of balancing is especially pronounced in the region of entropy minima, while for large numbers of topics the effect is less observable. This happens because that increase in the number of topics leads to an almost uniform distribution of word probabilities regardless of the dataset content.

**Figure 9:**
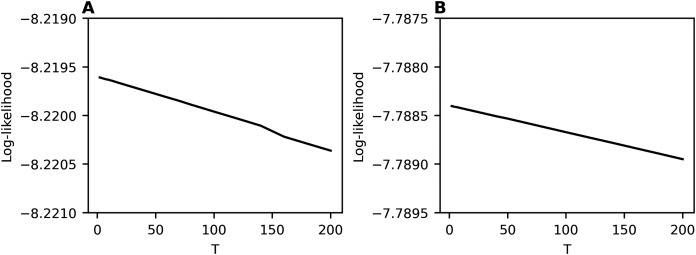
Log-likelihood curves (hARTM). (A) Lenta dataset. (B) Balanced WoS dataset.

Figure 9 demonstrates the behavior of log-likelihood depending on the number of topics for non-hierarchical and hierarchical datasets. Let us note that the behavior is monotone and does not allow determining the dataset structure.

## Discussion

As our experiments demonstrate, hLDA model is very unstable which means that its different runs with the same parameters produce radically different topical structures of the same data. This instability can be significantly reduced by changing parameter *η*, which controls the sparsity of topics. However, how to choose the optimal value of *η* is an open question since neither log-likelihood nor Renyi entropy allow us to tune this parameter.

A somewhat more promising result has been obtained for hPAM model which depends on the parameter *η* and the number of topics on different levels. Variation of these parameters in experiments demonstrated that Renyi entropy allows determining only one level of data structure for this model, namely, the number of topics on the second level. Variation of the number of topics on the next level leads to significant fluctuations of Renyi entropy for both non-hierarchical and hierarchical datasets. Correspondingly, it seems that sharp jumps of Renyi entropy on the third level are related to the model’s features rather than to the datasets’ structure. Additionally, the behavior of log-likelihood as a function of the number of topics and parameter *η* does not allow determining the dataset structure since the maxima and the minima of log-likelihood do not correspond to the structure of mark-up either for hierarchical or non-hierarchical datasets.

Among the considered models, hARTM is the most stable since the procedure of initialization of this model has almost no effect on the obtained values of Renyi entropy. Moreover, Renyi entropy for this model demonstrates that for the datasets with non-hierarchical mark-up (for both the English-language and the Russian-language datasets), there is only one minimum, which is located close to the number of topics from the mark-up. Further increase in the number of topics does not lead to jumps (in contrast to hPAM model) but leads to a smooth growth of Renyi entropy which coincides with the results of modeling with non-hierarchical topic models (such as Latent Dirichlet allocation (Blei, Ng & Jordan, 2003; Griffiths & Steyvers, 2004) and granulated Latent Dirichlet Allocation (Koltcov et al., 2016)) on the same datasets (Koltcov et al., 2020). However, for the datasets with hierarchical mark-up, Renyi entropy has two clear minima. One of these minima corresponds to the number of topics from the mark-up of the first level, and the second is close to the values from the mark-up of the second level. The difference between the number of topics obtained by searching for Renyi entropy minimum and the results of the mark-up is about 3-4 topics for the first level and 8-10 topics for the second level. Thus, estimation of the number of topics on the second level has a poorer quality.

To the best of our knowledge and based on our results, our approach is the first method allowing to detect the presence of hierarchical or non-hierarchical structure in the text data, and, thus, can be used as a hierarchy detector. However, it is useful only for hARTM model among the considered ones. At the same time, the behavior of log-likelihood significantly depends on the values of hyperparameters and often it has no clear maxima which, in turn, complicates the application of this metric for determining the optimal number of topics on different hierarchical levels. Moreover, since human mark-up also usually has its own limitations to precision or inter-annotator agreement, the achieved accuracy of prediction of the topic number with Renyi entropy can be viewed as considerable.

Based on the additional experiments with different types of data pre-processing presented in Appendix B, we also conclude that removing short and rare words leads to the decrease in Renyi entropy (and, correspondingly, to the increase in information). At the same time, the location of a global minimum stays almost unchanged. However, extensive pre-processing may lead to disappearance of local minima at the second hierarchical level. Therefore, it is recommended to remove short words and not to remove words with a frequency above 5. In general, cutting off the long tail of the frequency distribution affects the results of the entropy calculation, since Renyi entropy is calculated as the difference between high frequency words and low frequency words.

The proposed Renyi entropy approach has some limitations related to the method of Renyi entropy calculation and to the distribution of words in text collections. First, these distributions have long and heavy tails. Words from such tails are assigned to topics with small probabilities. Since Renyi entropy measures the difference between words with high probabilities and words with small probabilities, it can only detect topics that are comprised of highly probable words. However, lower levels of hierarchy tend to contain narrower topics, each of which is comprised of words with smaller probabilities. Correspondingly, the ability to distinguish the sets of topics disappears as the number of hierarchy level increases. Thus, Renyi entropy approach is suitable for determining the number of topics and values of hyperparameters for one or two levels of hierarchical structures. Another limitation of our approach is the presence of fluctuations in Renyi entropy values. These fluctuations are related to fluctuations of word probabilities, which, in turn, are caused by the stochastic nature of topic modeling. The problem of minimizing the instability of topic modeling has not yet been solved, but the stabilization of topic models through word embeddings can be potentially interesting. The application of Renyi entropy approach to topic models with word embeddings is beyond the scope of this work and could be considered a next potential stage in the development of the entropic approach to topic modeling.

## Conclusion

In this work, we investigated the ability of hierarchical topic models to correctly determine the hierarchical structure in data by applying three topic modeling algorithms to four datasets with a human mark-up of topics. Two of these datasets have a flat mark-up, while the mark-up of two others is two-level. Three of the datasets are in the English language, and one is in Russian. Thus, we analyze the models and test our approach on a variety of data structures and languages. We first formulated the principle of Renyi entropy calculation for hierarchical topic models. Next, we consecutively analyzed the chosen topic models using log-likelihood metric and Renyi entropy. Based on the results of our calculations, we can conclude the following.

First, our Renyi entropy-based approach can be extended to hierarchical topic models since the accuracy of the approximations of the optimal topic number for some of such models is not inferior to that for non-hierarchical models demonstrated in Koltcov (2018) and Koltcov et al. (2020). Second, our calculations on the test datasets demonstrated that hLDA model in its current form is not applicable to practical tasks due to its extreme instability (although it is open to regularization in future research). Third, for hPAM model, the proposed Renyi entropy approach allows selecting the number of topics only for one level of hierarchy. Determining the number of topics on the next levels is complicated by the large fluctuations of entropy for large numbers of topics. Thus, hPAM model, in conjunction with Renyi entropy as a metric to optimize, can be used for modeling datasets with non-hierarchical topical structures. Although this is suboptimal, let us note that log-likelihood metric does not allow tuning most of the model parameters on either hierarchical or flat data. Fourth, based on our calculations, it can be concluded that hARTM model provides the best results as, when applied to this model, Renyi entropy approach allows detecting both the hierarchical and non-hierarchical structures of the datasets. In the case of non-hierarchical dataset structure, we observe only one minimum of Renyi entropy under variation of the number of topics for both languages. In the case of a two-level dataset structure, Renyi entropy has two minima, each of which approximately corresponds to the topic number at the respective level of the hierarchy.

hARTM model is thus the easiest for tuning and, additionally, the most stable among the considered algorithms, while Renyi entropy is obviously a better quality metric compared to log-likelihood. We can also conclude that Renyi entropy is better than perplexity as the latter behaves very similar to log-likelihood whose derivative it is. Thus, for the purposes of topic number optimization it is recommended to select other model parameters based on the minimum of Renyi entropy.

The main limitation of Renyi entropy approach is that it can detect the true number of topics only for one or two levels of a topic hierarchy. Topic numbers at the subsequent levels are hard to predict since the probabilities of words at these levels get smaller and more uniform, thus determining the formation of less pronounced topics with unclear boundaries that, furthermore, are much more prone to random shifts from one run to another. This, in turn, hinders discrimination between genuine local minima of Renyi entopy and its random fluctuations. The problem of detecting poorly pronounced topics and the related task of topic model stabilization can possibly be solved by means of word embeddings technology, which appears a promising direction for a future research.

Our paper also provides important insights for the use of the entropic approach in a broader task of topic modeling parameter optimization. In general, optimal model parameters (be it *α*, *η* or the number of topics) are those which produce a solution with the best value of one or more metrics of quality. However, when such metric cannot be optimized directly, as it is in the case of the number of topics, or its optimization is very time-consuming, an optimizable substitute metric should be found, of which Renyi entropy is a promising example. While in this work we have shown the utility of Renyi entropy metric for finding an optimal number of topics, our earlier work (Koltcov, Ignatenko & Koltsova, 2019) also demonstrates that its two-parametric extension (Sharma–Mittal entropy) can also help selecting semantically stable solutions (which is usually very computationally complex)—*i.e*. it can point at such combinations of parameters that produce similar sets of most probable words in topics across multiple runs of the algorithm. Application of two-parametric entropy to hierarchical topic models is a future work.

However, apart from the stability and correctness of topic number identification, topic interpretability is of significant importance for topic modeling quality. This has not been explored yet in our framework. Furthermore, the very measurement of interpretability needs development. Few existing metrics, such as standard coherence (Mimno et al., 2011) or tf-idf coherence (Nikolenko, Koltcov & Koltsova, 2017) aim at measuring interpretability of individual topics, while methods of generalization of those for the entire topic solutions are multiple, and none has been tested. Moreover, the correspondence of coherence to interpretability as defined by humans is far from perfect (Nikolenko, Koltcov & Koltsova, 2017). Neither it is obvious that a single topic modeling solution could be found combining the best interpretability, stability and the optimal number of topics; these different measurements of quality might be competing. Our research, in addition to our previous investigation in Koltcov, Ignatenko & Koltsova (2019), points to the prospect of applicability of the entropic approach to simultaneous parameter optimization in topic modeling. For this, more research into topic interpretability and into multi-parametric entropies should be carried out.

## Appendix A

### Classic probabilistic topic models

Let us briefly discuss the most widely used topic models and the existing quality metrics in the field of topic modeling. Assume that we have a document collection *D* with a vocabulary of all unique words denoted by *W*. A document *d* contains a sequence of words {*w*_1_, *w*_2_,…,*w*_*n*_}, where *w*_*i*_ ∈ *W*. So, the words and the documents are the only observable variables. The goal of topic modeling is to retrieve hidden topics in the document collection, where each topic is characterized by its distribution over the vocabulary. Thus, the primary goal of topic modeling is to find the word distribution for each topic and to find the proportions of topics in each document. In most probabilistic topic models, it is assumed that, first, there exists a finite number *T* of topics, and each entry of a word *w* in document *d* is associated with a certain topic *t*. Second, it is assumed that the order of words in documents is not important for TM (‘bag-of-words’ model) and, in addition, the order of documents in the collection is neither important. Third, a conditional independence assumption states that document *d* and word *w* are independent conditioned on the latent topic *t*, *i.e*., words, conditioned on the latent class *t*, are generated independently of the specific document identity *d* (Hofmann, 2001).

Two basic topic models are probabilistic latent semantic analysis (pLSA) (Hofmann, 1999) and its Bayesian version called Latent Dirichlet Allocation (LDA) (Blei, Ng & Jordan, 2003). Mathematically, the probability of word *w* in document *d* can be expressed as follows (Hofmann, 1999):

}{}p(w|d)=\sum_{t\in T}p(w}|t})p(t|d)=\sum_{t\in T}\phi_{wt}\theta_{td}

where *t* is a topic, *p*(*w*|*t*) is the distribution of words by topics, and *p*(*t*|*d*) is the distribution of topics by documents. The output of TM is represented with two matrices, namely, matrix *Φ*: = {*ϕ*_*wt*_}≡ {*p*(*w*|*t*)} containing the distribution of words by topics and matrix *Θ*: = {*θ*_*td*_}≡ {*p*(*t*|*d*)} containing the distribution of topics by documents (or, in other words, the proportions of topics in documents). Many modifications of the LDA model were developed for various specific applications. However, these models share the same practical issue: to build a model, the user has to set the number of topics unknown in advance in many cases.

Generally, standard quality metrics such as perplexity (Heinrich, 2004; Newman et al., 2009; Zhao et al., 2015), log-likelihood (Wallach, Mimno & McCallum, 2009; Heinrich, 2004; Griffiths & Steyvers, 2004), and semantic coherence (Mimno et al., 2011; Stevens et al., 2012) are used to select the number of topics or to tune other model parameters. Perplexity is a metric that evaluates the efficiency of the model to predict the data. For LDA-based models, the perplexity for document collection }{}{\hat D} with *D* documents can be calculated as follows (Heinrich, 2004):

}{}{\rm perplexity}(\hat{D}) = {\mathop{\rm exp}\nolimits} \left( { - {{\sum\nolimits_d {\log } p(d)} \over {\sum\nolimits_d {{N_d}} }}} \right) = {\mathop{\rm exp}\nolimits} \left( { - {{\sum\nolimits_d {\sum\nolimits_w {n_d^w} } \ln (\sum\nolimits_t {{\phi _{wt}}} {\theta _{td}})} \over {\sum\nolimits_d {{N_d}} }}} \right),

where *N*_*d*_ is the number of words in document *d*, }{}c = \{ {c_d}\} _{d = 1}^D is the number of times word *w* was observed in document *d*. A lower perplexity value means better model quality. Perplexity is closely related to likelihood, namely, perplexity is a reciprocal geometric mean of the likelihood, where likelihood for a document *d* is expressed as (Heinrich, 2004)

}{}P(d|\Phi ,\Theta ) = \prod\limits_w {{{(\sum\limits_t {{\phi _{wt}}} {\theta _{td}})}^{n_d^w}}} .

In turn, log-likelihood of document collection \hat{D} can be calculated for LDA-based models as follows (Wallach, Mimno & McCallum, 2009; Heinrich, 2004):

\ln (P(\hat{D}|\Phi ,\Theta )) = \sum\limits_d {\sum\limits_w {n_d^w} } \ln (\sum\limits_t {{\phi _{wt}}} {\theta _{td}}).

The number of topics and the other model parameters are selected when finding maximum log-likelihood (Griffiths & Steyvers, 2004).

Semantic coherence is another type of quality metrics. It aims at measuring the interpretability of inferred topics (Mimno et al., 2011) rather than the predictive power of the model. Semantic coherence can be calculated as an average of the topic coherence scores, where each topic coherence score is expressed as

}{}C(t,W(t)) = \sum\limits_{m = 2}^M {\sum\limits_{l = 1}^{m - 1} {\log } } ({{D(v_m^t,v_l^t) + 1} \over {D(v_l^t)}}),

W(*t*) = (*v*_1_^t^, … , *v_M_^t^*) is a list of *M* most probable words in topic *t*, *D*(*v*) is the number of documents containing word *v*, and *D*(*v*,*v*′) is the number of documents where words *v* and *v*′ co-occur. The typical value for *M* is 5–20 words.

The discussion on the application of the above metrics to the task of determining the number of topics for pLSA and LDA models and limitations of these metrics can be found in papers (Koltcov, Ignatenko & Koltsova, 2019; Koltcov et al., 2020).

#### Nonparametric topic models

Further development of topic models occurred in the direction of nonparametric models. The main idea of nonparametric topic modeling is to infer the model structure (namely, the number of topics) from the data. Theoretically, nonparametric models are able to select the number of topics automatically according to available data. Such models introduce a prior distribution on potentially infinite partitions of integers using some stochastic process that would give an advantage in the form of a higher prior probability for solutions with fewer topics. In works (Teh et al., 2006; Teh et al., 2004), a topic model based on hierarchical Dirichlet process (HDP) was first proposed. This model can be considered as an infinite extension of LDA model (Heinrich, 2008). More complicated models that are based on the Indian buffet process are considered in works (Chen, Zhou & Carin, 2012; Williamson et al., 2010). Detailed surveys on nonparametric models can be found in Hjort et al. (2010) and Rasmussen & Williams (2006). However, nonparametric models possess a set of parameters that can significantly influence the inferred number of topics and results of TM in general (Vorontsov, Potapenko & Plavin, 2015). Moreover, in work (Koltcov et al., 2020), we demonstrated that, in real applications, the number of topics inferred by HDP model does not correspond to the number of topics obtained with human judgment. Thus, the application of this type of models is complicated in practice.

### Hierarchical topic models

The next important step in the development of topic models was dictated by the intention to organize topics into a hierarchy. It resulted in the development of hierarchical topic models. In contrast to flat topic models (such as pLSA or LDA), the hierarchical topic models are able to discover a topical hierarchy, which comprises levels of topical abstraction. Usually, each node in the hierarchy corresponds to a topic, which, in turn, is represented by the distribution over words. Different hierarchical topic models are based on different prior assumptions on the distribution of topics and on the type of hierarchical structure to be inferred. The two most widely used in practice hierarchical topic models (Liu et al., 2016) are hierarchical latent Dirichlet allocation (hLDA) (Blei et al., 2003) and hierarchical Pachinko allocation (hPAM) (Mimno, Li & McCallum, 2007).

hLDA model is a hierarchical and nonparametric extension of LDA model. In the framework of this model, it is assumed that each sub-topic (child topic) has a single parent topic, thus, providing a tree of topics. Moreover, it is assumed that all topics of a document are found within a path in that tree. It imposes significant restrictions on the inferred topical components of documents. Thus, for instance, a document can not be devoted to several specific sub-topics within the assumptions of the model. Another feature of this model is that the first level of the hierarchy always contains one topic (the root of the hierarchy). hLDA model learns topic hierarchies based on the nested Chinese Restaurant Process (nCRP), which specifies a prior for the model. Nested Chinese Restaurant Process is a hierarchical version of the Chinese Restaurant process and is used to express uncertainty about possible *L*-level trees. To illustrate nCRP, assume that there are infinitely many Chinese restaurants with infinitely many tables. One restaurant is associated with a root (level 1) and each table of this restaurant has a card with a reference to another restaurant (level 2). Tables of those restaurants, in turn, have references to other restaurants (level 3), and this structure repeats. Each restaurant is referred to exactly once, therefore, the structure produces an infinitely-branched tree of restaurants. Imagine that a tourist enters the root restaurant and selects a table according to

(2) \eqalign{  p({\rm occupied\;table\;i\;|\;previous\;customers}) = {{{m_i}} \over {\gamma + m - 1}} \cr  p({\rm new\;table\;|\;previous\;customers}) = {\gamma \over {\gamma + m - 1}}, \cr}

where *m*_*i*_ is the number of previous customers at table *i*, *m* is the number of customers in the restaurant, including the tourist, and *γ* is a parameter that controls how often a customer chooses a new table. The next day, the tourist goes to the restaurant identified on the card of the table chosen the day before and selects a table according to Eq. (2). This process is repeated for *L* days. After *M* tourists proceed this process, the collection of their paths describes a particular *L*-level tree. In terms of topic modeling, customers correspond to documents, and restaurants correspond to topics. In the framework of hLDA model, the following prior distributions are assumed: (1) nCRP prior with hyperparameter *γ* on possible trees; (2) symmetric Dirichlet prior with hyperparameter *η* on the distribution of words by topics *ϕ*_*wt*_; (3) *L*-dimensional Dirichlet prior with hyperparameter *α* on mixing proportions *θ*_*td*_ of the topics along the path from the root to the leaf.

The generative process of hLDA model can be described as follows:

- For each node *t*, draw *ϕ*_·__*t*_∼ *Dir(η)*.
- For each document d: draw a path of topics c_d_ = {c_d1_,…,c_dL_} according to nCRP with parameter γ (2); draw the topic mixing proportion θ_d_ ∼ Dir(α). For each position n of word in the document, choose the level assignment z_dn_∼ Mult(θ_d_) (as the level is chosen in the path, the topic is determined), then draw a word from the chosen topic w_dn_∼ Mult(φ· c_d_z_dn___).

Thus, a document is drawn by choosing an L-level path through the restaurants (topics) and then sampling the words from the L chosen topics. The inference can be approximated by means of Collapsed Gibbs sampling Blei, Griffiths & Jordan (2010) and Chen et al. (2018). The expressions for assessment of z_dn_ and c_d_ variables are the following:

}{}p({z_{dn}} = l|{w_{dn}} = v,W,{z_{\neg dn}},{\bf{c}}) \propto (a_{dl}^{\neg dn} + \alpha ){{b_{{c_{dl}},v}^{\neg dn} + \eta } \over {s_{{c_{dl}}}^{\neg dn} + V\eta }},

}{}p({{\bf{c}}_d} = \widetilde {\bf{c}}|W,z,{{\bf{c}}_{\neg d}}) \propto p(\tilde {\bf{c}}|{{\bf{c}}_{\neg d}})\prod\limits_{l = 1}^L {{{B(b_{{c_l}}^{\neg d} + b_{{c_l}}^d + \eta )} \over {B(b_{{c_l}}^{\neg d} + \eta )}}} ,

where *W* is the vocabulary (set of words), *V* is the vocabulary size, *z* = {*z*_*d*_}_*d* = 1_^*D*^, ¬* dn* means all the tokens excluding token *w*_*dn*_, }{}{\bf{c}} = \{ {{\bf{c}}_d}\} _{d = 1}^D\; ,\; a_{dl}^{\neg dn} is a counter that equals the number of tokens in level *l* in document *d* excluding token *w*_*dn*_, }{}b_{c_{dl},v}^{\neg dn} is a counter that equals the number of tokens of the word *v* assigned to topic *c*_*dl*_ excluding the current token *w*_*dn*_, }{}s_{c_{dl}}^{\neg dn} is a counter that equals the number of tokens assigned to topic *c*_*dl*_ excluding the current token *w*_*dn*_, \tilde {\bf{c}} = \{ {c_1},\ldots,{c_L}\} is a path in the hierarchy, }{}b_{c_{l}}^{\neg d} is a counter that equals the number of tokens assigned to topic *c*_*l*_ excluding the tokens of document *d*, }{}b_{c_{l}}^{d} is the number of tokens of document *d* that were assigned to topic *c*_*l*_, *B*(·) is the multivariate beta function and }{}p(\tilde {\bf{c}}|{{\bf{c}}_{\neg d}}) is according to nCRP (2). The details can be found in Chen et al. (2018). Then, matrix *Φ* is calculated according to

}{}{\phi _{wt}} = \displaystyle{{{c_{wt}} + \eta } \over {\sum\nolimits_{v = 1}^V {c_{vt}} + \eta V}},

where a counter *c*_*wt*_ equals the number of instances word *w* was assigned to topic *t*.

We would like to note that hLDA model has three hyperparameters: (1) *α* is a parameter of Dirichlet distribution, which controls smoothing over levels in the tree; (2) *η* is hyperparameter of another Dirichlet distribution, which controls the sparsity of topics; (3) *γ* is a parameter of the nested Chinese restaurant process, which controls how often a document will choose new, *i.e*., not previously encountered paths.

In hPAM model, in contrast to hLDA, it is assumed that each child topic can be related to each node (topic) from the upper level, thus, resulting in a directed acyclic graph of topics. Therefore, in hPAM model, a child topic has several parent topics. It reduces the necessity of inferring the correct tree structure that is crucial in hLDA. Moreover, it allows the documents to contain several specific sub-topics. Moreover, hPAM model always infers a three-level hierarchy, where the first level corresponds to a root topic, the second level corresponds to super-topics, and the third level contains sub-topics. Another important difference between hLDA and hPAM models is that hLDA is a nonparametric model, *i.e*., it infers the number of topics on each level automatically while hPAM model is parametric, therefore, a user has to manually select the number of topics on each level. Thus, the number of topics on each level of the hierarchy is a model parameter. Moreover, hPAM model has two hyperparameters: *η* is a parameter of a prior Dirichlet distribution for *ϕ*_*t*_, which controls sparseness of topics, and *α* is a parameter of a prior Dirichlet distribution for *θ*. Let us note that since hPAM constructs a directed acyclic graph, each node (topic) at a given level has a distribution over all nodes on the next lower level. Let *T*_1_ be the number of ‘super-topics’ and *T*_2_ be the number of ‘sub-topics’.

The generative process of hPAM model is as follows (hPAM model 2):

- For each vertex (topic) *t* of the graph, draw *ϕ*_·__*t*_∼ *Dir*(*η*).
- For each document *d*, sample *θ*_0_ from a *T*_1_ + 1-dimensional Dirichlet distribution with hyperparameter *α*_0_ and distribution *θ*_*T*_ from a *T*_2_ + 1-dimensional Dirichlet distribution with hyperparameter *α*_*T*_ for each super-topic. The first component of *θ*_0_ determines the probability of the event that a word is generated by the root topic while the other *T*_1_ components of *θ*_0_ determine the distribution of the root topic over super-topics. Analogously, the first component of *θ*_*T*_ defines the probability of the event that a word is generated by the super-topic *T*, and the other *T*_2_ components determine the distribution of the super-topic *T* over sub-topics.
- For each word *w*: sample a super-topic *z*_*T*_ ∼ *Mult*(*θ*_0_). If *z*_*T*_ = 0, sample a word from *ϕ*_·__0_ ∼ *Dir*(*η*). Otherwise, sample a sub-topic *z*_*t*_ ∼ *Mult*(*θz*_*T*_). If *z*_*t*_ = 0, then sample a word from *ϕ*_·__*z_T_*_∼ *Dir*(*η*). Otherwise, sample a word from *φ*_*z_t_*_.

Thus, matrix of the distribution of words by topics has dimension *W* × (*T*_1_ + *T*_2_ + 1). The inference algorithm is based on Gibbs sampling method Mimno, Li & McCallum (2007). Sampling distribution for a given word *w* in document *d* is as follows:

(3) }{}p({z_T} = x,{z_t} = y|{w_{dn}} = w,W,{{\bf z}_{\neg w}}) \propto (a_{dx}^{\neg dn} + {\alpha _{0,x}}) \cdot \displaystyle{{b_{d,x,y}^{\neg dn} + {\alpha _{x,y}}} \over {\sum\nolimits_{y = 1}^{{T_2}} (b_{d,x,y}^{\neg dn} + {\alpha _{x,y}})}} \cdot \displaystyle{{c_{wk}^{\neg dn} + \eta } \over {\sum\nolimits_w (c_{wk}^{\neg dn} + \eta )}},

where *x* ∈ {0,1,…, *T*_1_} is the index of super-topic if *x* ≠ 0; *y* ∈ {0,1,…,*T*_2_} is the index of sub-topic if *y* ≠ 0; ***z***_*¬w*_ is the topic assignments for all other words, counter }{}a_{dx}^{\neg dn} equals the number of tokens (excluding the current tokem *w_dn_*) in document *d* that were assigned to super-topic *x*, *x* ≠ 0, and to the root topic if *x* = 0; *α_0,x_* means the *x*-th component of vector *α_0_*; counter }{}b_{d, x, y}^{\neg dn} equals the number of tokens (excluding the current token *w_dn_*) in document *d* that were assigned to super-topic *x* and sub-topic *y*; counter }{}c_{wk}^{\neg dn} equals the number of tokens of the word w that were generated by topic *k*, where *k* is the root topic if *x* = 0, *k* = 1, …,*T*_1_ is a super-topic if *y* = 0 and *k* = *T*_1_ + 1, …, *T*_2_ + 1 is a sub-topic otherwise. Then, a pair of indices (*x*, *y*) is sampled from the distribution (3) and assigned to the current word w_dn_. Matrix *Φ* is calculated according to

}{}{\phi _{wt}} = \displaystyle{{{c_{wt}} + \eta } \over {\sum\nolimits_{v = 1}^V {c_{vt}} + \eta V}},

where a counter *c*_*wt*_ equals the number of instances word *w* was generated by topic *t*. One can use the fixed point update equations described in Minka (2000) to optimize the asymmetric Dirichlet parameters *α*. Thus, in most of the publicly available model implementations hyperparameter *η* is fixed and has to be set by a user while hyperparameters *α*_*t*_ are optimized during the training step.

Besides hLDA and hPAM, many other hierarchical topic models have been developed, for instance, nested hierarchical Dirichlet process (nHDP) (Paisley et al., 2015), hierarchical probabilistic latent semantic analysis (hPLSA) (Gaussier et al., 2002), topic hierarchies of Hierarchical Dirichlet Processes (hHDP) (Zavitsanos, Paliouras & Vouros, 2011), hierarchical latent tree analysis (HLTA) (Chen et al., 2017), hierarchical stochastic block model (hSBM) (Gerlach, Peixoto & Altmann, 2018), and hierarchical Additive Regularization of Topic Models (hARTM) (Chirkova & Vorontsov, 2016).

To relax the restrictions of hLDA that each document follows one path down the tree of topics, nHDP model was proposed (Paisley et al., 2015). Two main changes with respect to hLDA model are the following: (1) each word follows its own path to a topic; (2) each document has a distribution on possible paths in a shared tree. In the framework of this model, all documents share a global nCRP drawn according to the stick-breaking construction. Let us denote this tree by *T*. From a root Dirichlet process *G*_*i*0_, a path is followed by drawing *ϕ*_*l*__+ 1_ ∼ *G*_*il*_ (*G*_*il*_ denotes Dirichlet process for the children of node *i*_*l*_,) for *l* = 0,1,2,…, where *i*_0_ is the root index, and *i*_*l*_ = (*i*_1_,…,*i*_*l*_) indexes the Dirichlet process associated with the topic *ϕ*_*l*_ = *θ*_*il*_. For each document *d*, a tree *T*_*d*_ is constructed, where for each *G*_*il*_, a corresponding }{}G_{{i_l}}^{(d)} \in {T_d} is drawn according to the Dirichlet process: }{}G_{{i_l}}^{(d)}\sim DP(\beta {G_{{i_l}}}). Using this construction, each document has a tree with document specific transition probabilities defined over the same subset of nodes. To generate a document with this tree, for each node *i*_*l*_, a document specific beta random variable is drawn. Namely, given that the path for word *w*_*d*,*n*_ is at node *i*_*l*_, stop with probability *U*_*d*,__*il*_ ∼ *Beta*(*γ*_1_, *γ*2). If we do not select topic *θ*_*il*_, then continue by selecting node *i*_*l*__+ 1_ according to }{}{G_{i_l^d}}. Thus, for word *w*_*d*,*n*_ in document *d*, the probability that it is generated by topic *ϕ*_*dn*_ = *θ*_*il*_ is as follows }{}Pr({\phi _{d,n}} = {\theta _{{i_l}}}|{T_d},{U_d}) = \left( {\prod\nolimits_{m = 0}^{l - 1} {G_{{i_m}}^{(d)}} (\{ {\theta _{{i_{m + 1}}}}\} )} \right) \cdot \left( {{U_{d,{i_l}}}\prod\nolimits_{m = 1}^{l - 1} {(1 - {U_{d,{i_m}}})} } \right). The left term is the probability of path *i*_*l*_, the right term is the probability that the word selects the *l*th topics, but not the first *l* − 1 topics, *i*_*m*_ denotes the first *m* values in *i*_*l*_ (*m*≤ *l*). The authors propose a stochastic variational inference (Paisley et al., 2015), which is scalable to large datasets, for this model.

In the framework of hPLSA, the generative process is as follows. First, a document class *α* is chosen with probability *p*(*α*) from a predefined number of classes. Second, a document *d* is chosen according to conditional probability *p*(*d*|*α*). Third, a topic for each word position is chosen according to class-conditional probability *p*(*t*|*α*). Forth, a word is sampled according to *p*(*w*|*t*). In the hierarchical structure of hPLSA, the document classes are the leaves of the hierarchy while topics occupy non-leaf nodes of the hierarchy. pLSA model is a special case of hPLSA since if only one topic per class is sampled then the result corresponds to the flat topic solution of pLSA. If a topic is shared among classes, then it is placed at a higher level of the hierarchy. hPLSA model has certain limitations, which are analogous to limitations of pLSA, namely, it possesses a large number of parameters that have to be estimated, and this number grows linearly with the size of the dataset. It can lead to model overfitting.

In the framework of hHDP, two models are proposed. The first one (hvHDP) results in a hierarchy, where internal nodes are represented as probability distributions over topics and over words. Thus, in hvHDP model, all nodes can be considered as topics. In the second model (htHDP), only leaf nodes are represented as distributions over words. Thus, only the leaf nodes are essentially the topics. Both models are non-parametric and exploit the mixture model of hierarchical Dirichlet processes (HDP). At each level of the hierarchy, there is a Dirichlet process for each document and a global Dirichlet process over all the Dirichlet processes at that level. Thus, each level is associated with a HDP. These assumptions allow inferring the number of nodes on each level automatically. According to the authors (Zavitsanos, Paliouras & Vouros, 2011), hPAM and hLDA are the closest relatives of hvHDP in terms of inferred hierarchical structure. Indeed, analogously to hLDA, hHDP model is able to infer the number of topics automatically. In addition, analogously to hPAM, hHDP allows a child node to have several parent nodes, thus providing a more flexible structure. In turn, htHDP resembles the PAM model (Li & McCallum, 2006) since the words in both models are generated only at the leaf level. In contrast to PAM, htHDP is fully non-parametric and is able to infer the depth of the hierarchy and the number of nodes at each level. However, hHDP has a set of hyperparameters (*H*, *α*, *γ*) that can potentially lead to different hierarchies in terms of the depth and the number of nodes (topics) on each level.

HLTA is a probabilistic hierarchical topic model, however, it has significant differences with respect to LDA-based models. First, HLTA models a collection of documents without specifying a document generation process. The latent variables are unobserved attributes of the documents. Second, each observed variable is related to a word and is a binary variable that represents the presence or absence of the word in a document. Third, topics in HLTA are clusters of documents. Namely, each binary latent variable in HLTA partitions a document collection into two soft clusters of documents. The document clusters are interpreted as topics. HLTA provides a tree-structured model, where the word variables are at the leaves and the latent variables are at the internal nodes. In turn, each latent variable can be described by a set of top words according to their mutual information, *i.e*., by a set of words that are the best ones to characterize the difference between the two clusters due to the fact that their occurrence probabilities in the two clusters differ the most. Latent variables at high levels of the hierarchy correspond to more general topics, while latent variables at low levels correspond to more specific topics. The construction of the hierarchy is based on the subsequent application of flat models. The details of the model construction can be found in the original work (Chen et al., 2017).

The authors of hSBM model propose an approach that relates topic modeling and community detection in complex networks. Here, the word-document matrix is considered a bipartite network, and the problem of inferring topics becomes a problem of inferring communities. The authors claim that their nonparametric approach automatically determines the depth of the hierarchy and the number of groups for both documents and words. Let us note that this model clusters both documents and words into hierarchical categories separately. Thus, hSBM model splits the network into groups on different levels, which are organized as a hierarchical tree. The construction of the hierarchy is based on the subsequent application of stochastic block models (which were originally developed for community detection in networks) yielding a hierarchy of nested stochastic block models, where each level clusters the groups of the levels below. A detailed description of adaptation of stochastic block models to topics modeling can be found in Gerlach, Peixoto & Altmann (2018).

hARTM is a hierarchical version of the proposed earlier Additive Regularization of Topic Models (ARTM) approach. In the framework of hARTM, it is allowed for a topic to have several parent topics and, moreover, the authors claim that the model can automatically determine the number of sub-topics for each topic (Chirkova & Vorontsov, 2016). However, the number of topics on each level of the hierarchy has to be specified by a user. To construct a hierarchy, it is proposed to learn several flat topic models and then to tie them *via* regularization. Thus, for already learned *ϕ*^*l*^, *i.e*., the matrix containing the distribution of words by topics for topics on level *l*, it is proposed to implement matrix decomposition *ϕ*^*l*^ ∼ *ϕ*^*l*^ ^+ 1^ *ψ*, where matrix *ψ* = {*p*(*t*^*l*^ ^+ 1^|*t*^*l*^)} contains interlevel distributions of sub-topics *t*^*l*^ ^+ 1^ in parent topics *t*^*l*^, *ϕ*^*l*^ ^+ 1^ is the matrix containing the distribution of words by sub-topics *t*^*l*^ ^+ 1^ with additional sparsing regularizers. So, one can infer the hierarchy level by level *via* finding parent topics for each sub-topic using interlevel regularizers. A more detailed description of the model and the model inference can be found in work (Chirkova & Vorontsov, 2016).

In addition to a wide variety of unsupervised hierarchical topic models, many semi-supervised and supervised extensions of hLDA have been proposed. For instance, supervised hierarchical latent Dirichlet allocation (SHLDA) (Nguyen, Boyd-Graber & Resnik, 2013), constrained hierarchical Latent Dirichlet Allocation (constrained-hLDA) (Wang et al., 2014), hierarchical labeled-LDA (HLLDA) (Petinot, McKeown & Thadani, 2011), and semi-supervised hierarchical latent Dirichlet allocation (SSHLDA) model (Mao et al., 2012).

Although many hierarchical topic models have been developed, there are still no well-established quality metrics for topic hierarchies (Chen et al., 2017; Zavitsanos, Paliouras & Vouros, 2011; Belyy et al., 2018). Therefore, hierarchical topic models are often compared by the same quality metrics as flat models, namely, by means of per-word log-likelihood (Chambers, Smyth & Steyvers, 2010; Chen et al., 2017), perplexity (Zavitsanos, Paliouras & Vouros, 2011) or semantic coherence (Chen et al., 2017). However, log-likelihood and perplexity are criticized for their inability to account for the interpretability of topic solutions that, in turn, is essential for end-users. Moreover, it was demonstrated that improved likelihood may lead to lower interpretability (Chang et al., 2009). In contrast, semantic coherence is closer to the human evaluation of topic modeling output, however, it measures only topic interpretability and does not take into account the parent-child relations in topic hierarchies (Belyy et al., 2018). Therefore, semantic coherence only partially reflects the quality of a hierarchical model ignoring the hierarchical relations of topics.

## Appendix B

To estimate the influence of datasets pre-processing on the behavior of Renyi entropy in hierarchical models, the following experiments were conducted. First, let us describe additional pre-processing steps. At the first stage, words of length less than three letters were removed. At the second stage, in addition to short words, rare words with frequencies below 5 were also removed. For instance, after the initial pre-processing used in the main text of the paper, the 20 Newsgroups dataset consists of 50,965 unique words. After the first stage of additional pre-processing, the size of vocabulary is 25,956, and after the second stage it is 19,086 unique words. Analogously, Balanced WoS dataset contains 36,488 unique words in the experiments described in the main text. After the first stage of additional pre-processing it contains 31,725 unique words and 14,526 words after the second stage. For the obtained datasets with different types of pre-processing, hPAM and hARTM models were implemented to study the behavior of Renyi entropy in the same way as in section ‘Computational experiments’ of the main body of the article.

### hPAM model (Influence of pre-processing on entropy of the second hierarchical level)

**Figure A1:**
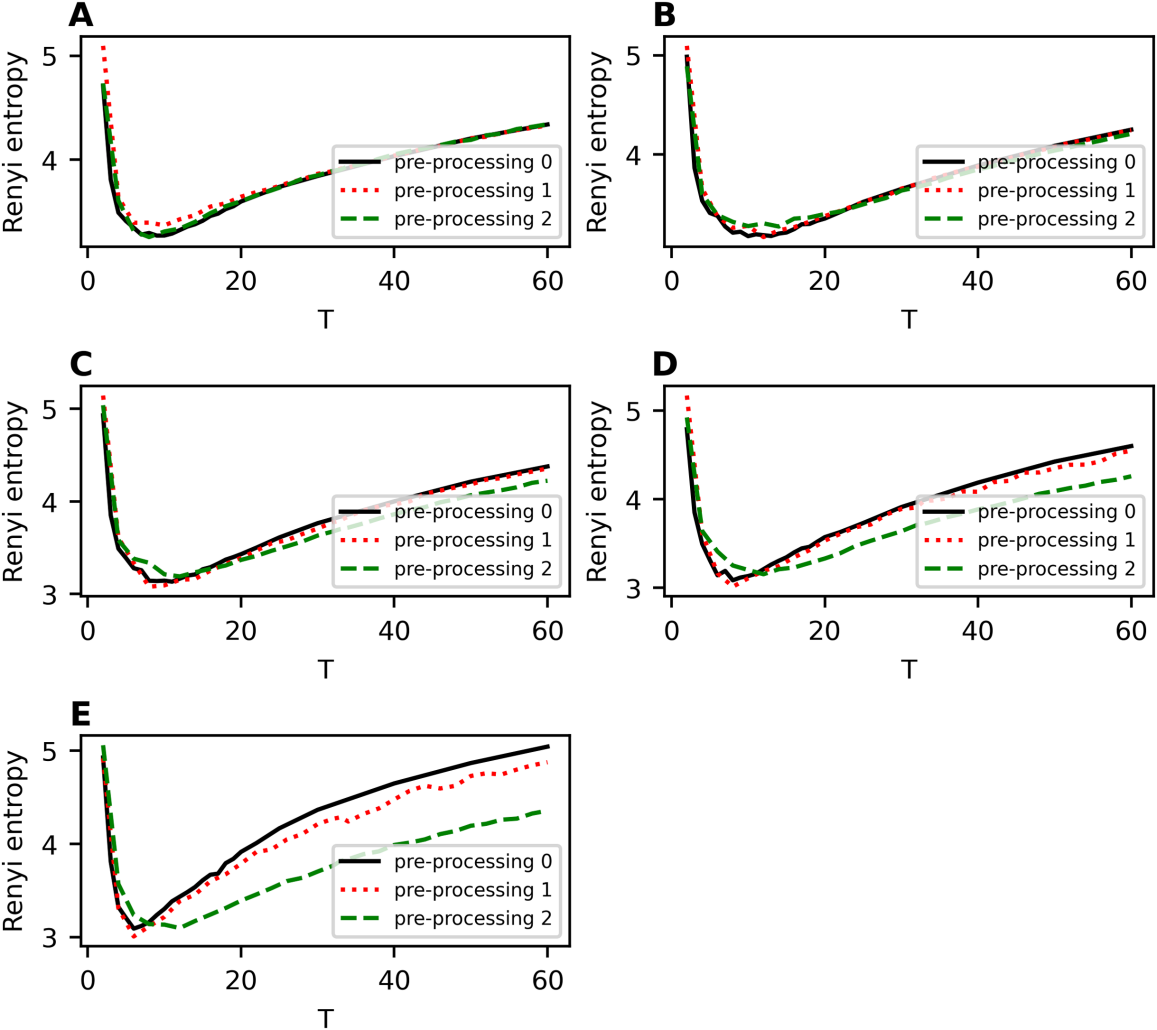
Renyi entropy curves (hPAM). 20 Newsgroups dataset. (A) *η* = 0.001. (B) *η* = 0.3. (C) *η* = 0.5. (D) *η* = 0.7. (E) *η* = 1.

Figure A1 demonstrates Renyi entropy curves on the second hierarchical level for 20 Newsgroups dataset for different values of parameter *η*. Here and further, “pre-processing 0” refers to the initial pre-processing; “pre-processing 1” refers to the first stage of additional pre-processing; “pre-processing 2” refers to the second stage of additional pre-processing. Based on the calculations, one can conclude the following. Removing only short words slightly worsens Renyi entropy, with the worsening being only in raising the entropy curve. The range of topics, where minima of Renyi entropy are observed, corresponds to 6–12 topics for the dataset with the first stage of additional pre-processing. The closest result to the human markup is observed at *η* = 0.3. Removing short and rare words leads to entropy curve raising and shifting of minima points. The range of minima points for the second stage of pre-processing corresponds to 8–14 topics. Since human mark up corresponds to 14–20, one can conclude that additional pre-processing with removing of short and rare words has a positive effect on the results of topic modeling on a dataset with a non-hierarchical labeling.

**Figure A2:**
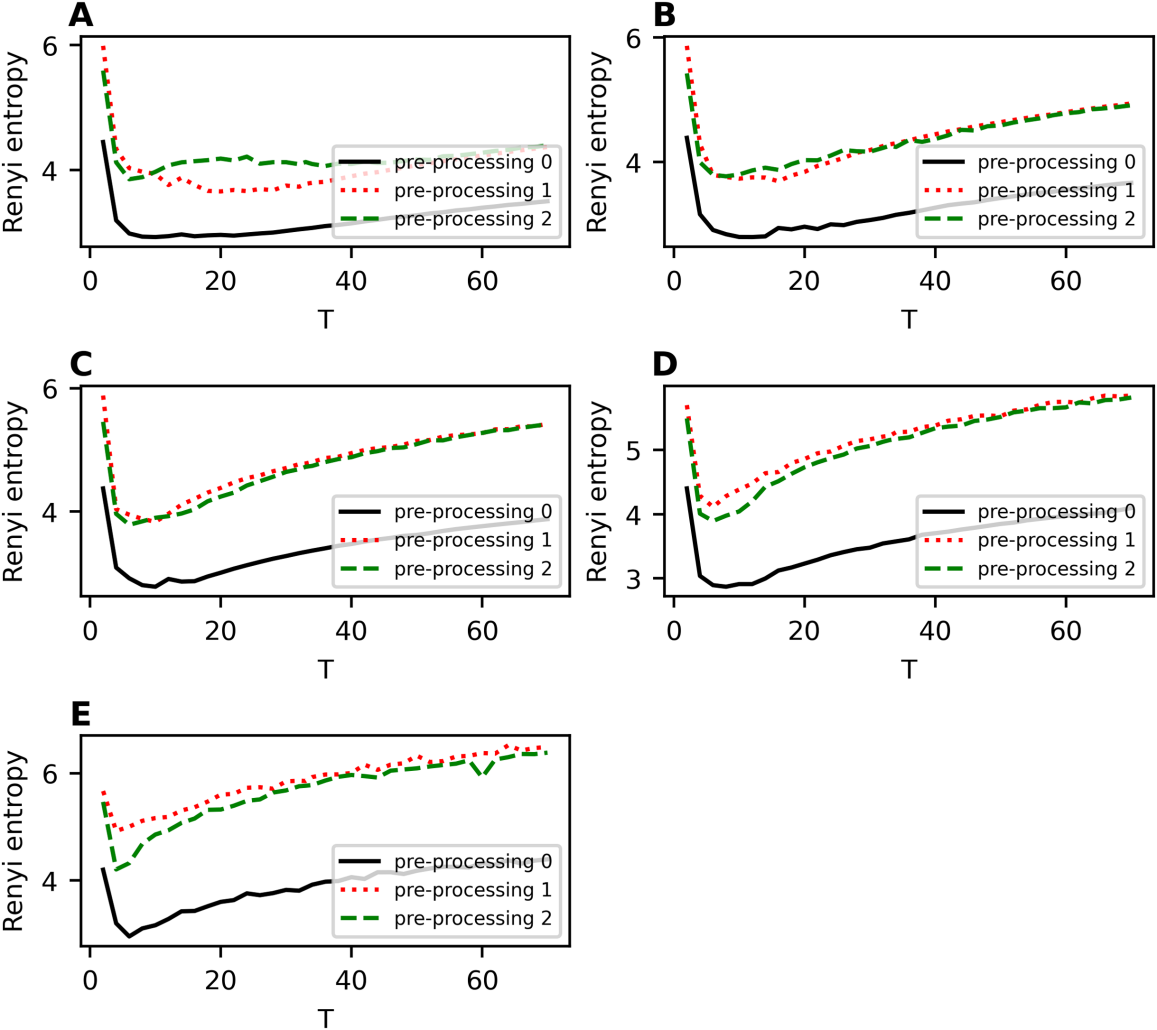
Renyi entropy curves (hPAM). Balanced WoS dataset. (A) *η* = 0.001. (B) *η* = 0.3. (C) *η* = 0.5. (D) *η* = 0.7. (E) *η* = 1.

Figure A2 demonstrates the results for balanced WoS dataset. One can see that removing only short words leads to raising of the whole Renyi entropy curve, however, the overall structure of the curve does not change. The range of topics with minimum Renyi entropy corresponds to 6-10 topics, *i.e*., there is a small variation associated with the change in parameter *η*. Removing of short and rare words leads to a back-down shift of Renyi entropy. Moreover, variation of parameter *η* does not influence significantly the minimum location under condition of this type of pre-processing. In general, Renyi entropy minimum corresponds to six topics that, in turn, is very close to close to the human mark-up. Thus, extensive pre-processing is necessary to obtain a better result of topic modeling.

### hPAM model (Influence of pre-processing on entropy of the third hierarchical level)

**Figure A3:**
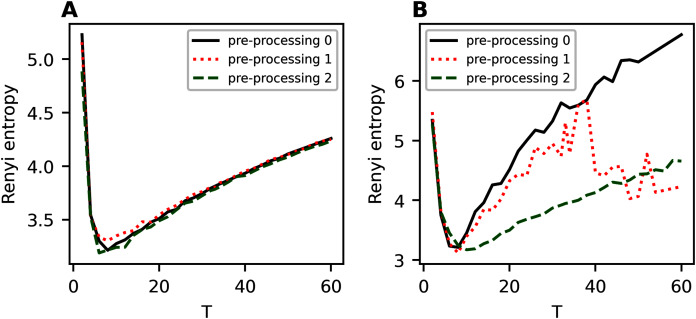
Renyi entropy curves (hPAM). 20 Newsgroups dataset. (A) *η* = 0.01, T_1_ = 10. (B) *η* = 0.7, T_1_ = 8.

Figure A3 demonstrates Renyi entropy curves on the third hierarchical level for 20 Newsgroups dataset for fixed values of *η* and *T*_1_. The results demonstrate that removing short and rare words improves Renyi entropy. For instance, sharp fluctuations appearing for hPAM model may disappear with a more extensive pre-processing.

**Figure A4:**
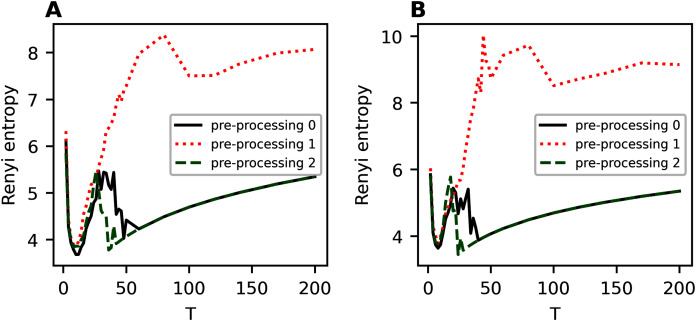
Renyi entropy curves (hPAM). Balanced WoS dataset. (A) *η* = 0.3, T_1_ = 12. (B) *η* = 0.5, T_1_ = 10.

Figure A4 demonstrates Renyi entropy curves on the third hierarchical level for balanced WoS dataset. One can see that in the region of the first entropy minimum, the difference between Renyi entropy curves for different pre-processing procedures is very small. A significant difference occurs where the HPAM model deteriorates, that is, for more than 25 topics. In addition, the second stage of pre-processing leads to smaller entropy values.

### hARTM model (Influence of pre-processing on entropy of the first hierarchical level)

**Figure A5:**
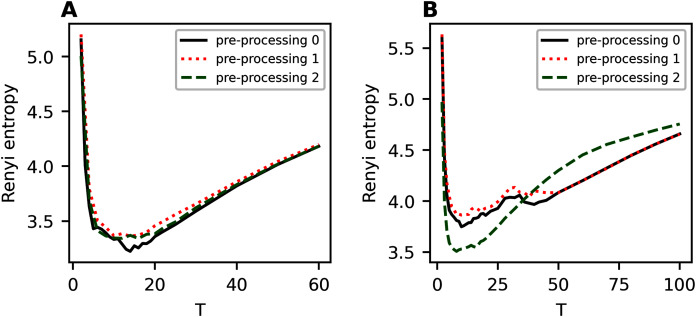
Renyi entropy curves (hARTM). (A) 20 Newsgroups dataset. (B) balanced WoS dataset.

Figure A5 demonstrates Renyi entropy curves for hARTM model on the first hierarchical level for two datasets, 20 Newsgroups, and balanced WoS. One can see that different types of pre-processing have almost no effect on the resulting Renyi entropy curves for 20 Newsgroups dataset, *i.e*., the dataset with non-hierarchical structure. For all types of pre-processing, the minimum corresponds to 14 topics, which is close to the human markup. Thus, hARTM model is stable and reproduces the results in terms of Renyi entropy. For balanced WoS dataset, we obtain that Renyi entropy curves are similar for initial and for the first additional pre-processing, namely, they have two clear minima corresponding to two hierarchical levels. However, more extensive pre-processing leads to the disappearance of the second local minimum in the region of 38 topics and the appearance of a small, not clearly pronounced minimum in the region of 16-18 topics. In addition, Renyi entropy curve for more extensive pre-processing is lower on average, which means a slightly better model.

### hARTM model (Influence of pre-processing on entropy of the second hierarchical level)

**Figure A6:**
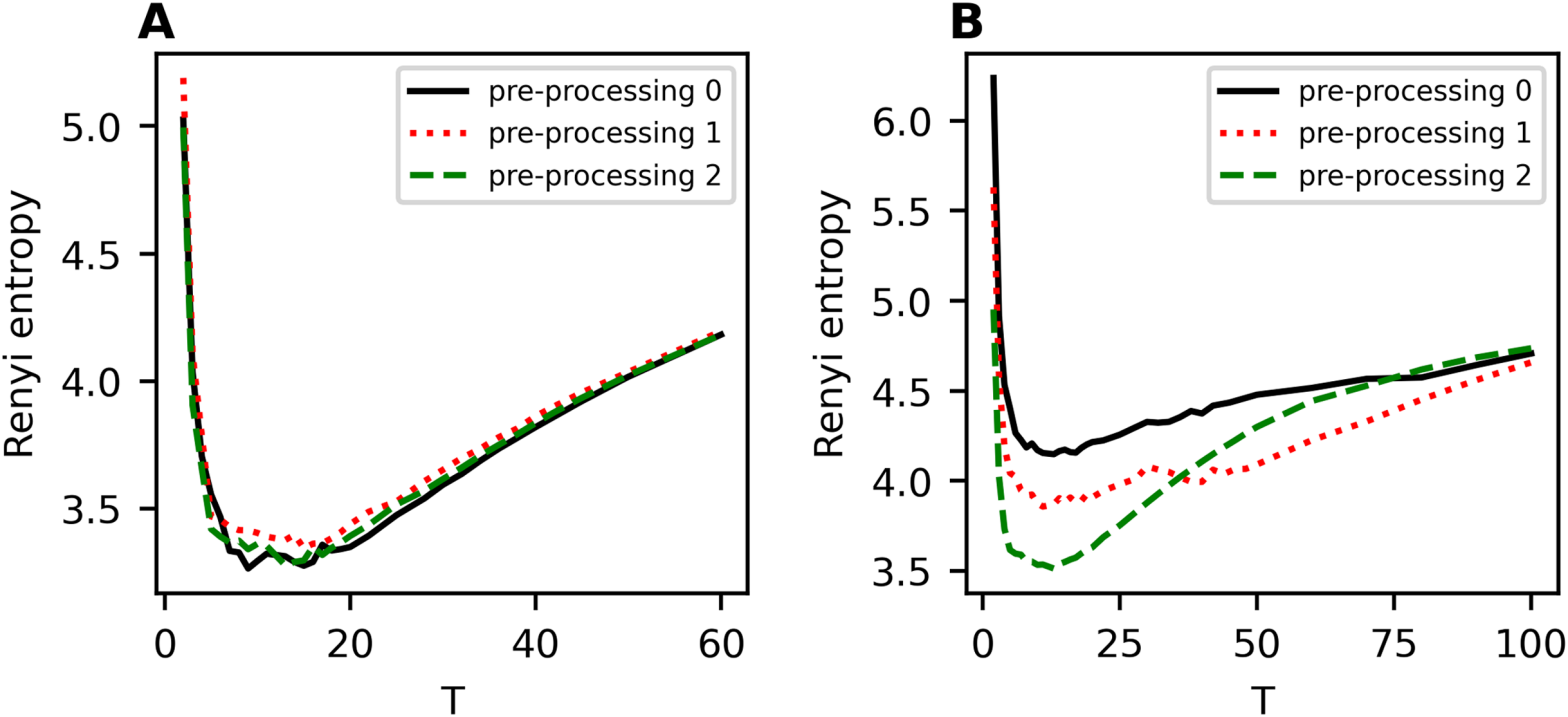
Renyi entropy curves (hARTM). (A) 20 Newsgroups dataset. (B) balanced WoS dataset.

Figure A6 demonstrates Renyi entropy curves for the hARTM model on the second hierarchical level for two datasets. Again, for the 20 Newsgroups dataset, we observe that different types of pre-processing have almost no effect on Renyi entropy curves and entropy minimum corresponds to 14–15 topics. In general, the behavior of Renyi entropy curves on the second level is almost identical to that of the first level. For the balanced WoS dataset, extensive pre-processing leads to smaller entropy values on average; however, it also leads to disappearance of the second local minimum.

## Supplemental Information

10.7717/peerj-cs.608/supp-1 Supplemental Information 1 Lemmatized and balanced subset of Amazon dataset. Each row contains lemmatized texts of product reviews from Amazon.

10.7717/peerj-cs.608/supp-2 Supplemental Information 2 Lemmatized texts of the 20 Newsgroups dataset. Each row contains lemmatized news texts.

10.7717/peerj-cs.608/supp-3 Supplemental Information 3 Lemmatized texts from Amazon dataset. Each row contains lemmatized texts of product reviews from Amazon in CSV format.

10.7717/peerj-cs.608/supp-4 Supplemental Information 4 Jupyter notebooks for Renyi entropy calculation (for hLDA, hPAM, hARTM models). Each notebook takes a lemmatized dataset as an input, runs a hierarchical topic model for a range of model parameters and calculates Renyi entropy on each level of hierarchical solution.

10.7717/peerj-cs.608/supp-5 Supplemental Information 5 Lemmatized news texts from Lenta.ru. Each row contains lemmatized texts of news articles from Lenta.ru news agency in csv format.

10.7717/peerj-cs.608/supp-6 Supplemental Information 6 'Requirements' file. The versions of the packages used in experiments.

10.7717/peerj-cs.608/supp-7 Supplemental Information 7 Lemmatised texts from 20 Newsgroups dataset after removing short words. Each row contains lemmatized texts of news texts from 20 Newsgroups dataset in CSV format (short words are removed).

10.7717/peerj-cs.608/supp-8 Supplemental Information 8 Lemmatized texts from 20 Newsgroups dataset after removing short and rare words. Each row contains lemmatized texts from 20 Newsgroups dataset in CSV format after removing short and rare words.

10.7717/peerj-cs.608/supp-9 Supplemental Information 9 Balanced subset of Web of Science dataset. Each row contains lemmatized texts of abstracts from Web of Science in csv format.

10.7717/peerj-cs.608/supp-10 Supplemental Information 10 Lemmatized texts from balanced WoS dataset after removing short and rare words. Each row contains lemmatized texts from balanced WoS dataset in CSV format after removing short and rare words.

10.7717/peerj-cs.608/supp-11 Supplemental Information 11 Lemmatized texts from balanced WoS dataset after removing short words. Each row contains lemmatized texts from balanced WoS dataset in CSV format after removing short words.

10.7717/peerj-cs.608/supp-12 Supplemental Information 12 Examples of top words from topical solutions for 20 Newsgroups dataset and balanced WoS dataset.

10.7717/peerj-cs.608/supp-13 Supplemental Information 13 Lemmatized texts from Web of Science dataset. Each row contains lemmatized texts of abstracts from Web of Science in csv format.

10.7717/peerj-cs.608/supp-14 Supplemental Information 14 Matrices of the distribution of words by topics for some topical solutions for 20 Newsgroups and balanced WoS datasets. The first column corresponds to unique words, other columns conatin probability distributions for topics.

## Additional Information and Declarations

### Data Availability

The following information was supplied regarding data availability: Datasets and code are available at GitHub: https://github.com/hse-scila/hierarchical-models. 20 Newsgroups dataset is available at GitHub: https://github.com/hse-scila/hierarchical-models/blob/master/datasets/20news_lem.csv. Lenta dataset is available at GitHub: https://github.com/hse-scila/hierarchical-models/blob/master/datasets/lenta_lem.csv. Amazon dataset is available at GitHub: https://github.com/hse-scila/hierarchical-models/blob/master/datasets/Amazon_lem.csv. Amazon balanced dataset is available at GitHub: https://github.com/hse-scila/hierarchical-models/blob/master/datasets/Amazon_balanced_lem.csv. WoS dataset is available at GitHub: https://github.com/hse-scila/hierarchical-models/blob/master/datasets/WoS_lem.csv. WoS balanced dataset is available at GitHub: https://github.com/hse-scila/hierarchical-models/blob/master/datasets/WoS_balanced_lem.csv.
