# Supplementary material for: Analysis and tuning of hierarchical topic models based on Renyi entropy approach
Source: PeerJ Comput Sci. 2021 Jul 29;7:e608. doi: 10.7717/peerj-cs.608 (PMC8330431; doi:10.7717/peerj-cs.608)
Supplement: Supplemental Information 12 [file peerj-cs-07-608-s012.pdf]

Top words of topics obtained with hARTM model on the first hierarchical level for 20 Newsgroups dataset.

T1

['encrypt', 'chip', 'clipper', 'secur', 'govern', 'phone', 'system', 'other', 'which', 'public', 'some', 'your', 'algorithm', 'messag', 'onli', 'articl', 'more', 'escrow', 'privaci', 'could', 'inform', 'know', 'need', 'number', 'like', 'make', 'technolog', 'secret', 'been', 'their', 'protect', 'even', 'netcom', 'comput', 'data', 'just', 'david', 'devic', 'than', 'agenc', 'access', 'time', 'enforc', 'such', 'wiretap', 'communic', 'peopl', 'them', 'then', 'work']

T2

['thank', 'know', 'drive', 'card', 'anyon', 'pleas', 'work', 'like', 'need', 'articl', 'driver', 'just', 'appl', 'problem', 'help', 'system', 'comput', 'mail', 'which', 'some', 'monitor', 'want', 'look', 'disk', 'onli', 'scsi', 'video', 'also', 'could', 'price', 'good', 'appreci', 'machin', 'board', 'email', 'info', 'them', 'control', 'more', 'hard', 'sound', 'advanc', 'when', 'your', 'other', 'time', 'speed', 'think', 'than', 'these']

T3

['game', 'year', 'team', 'play', 'articl', 'player', 'more', 'last', 'think', 'like', 'time', 'than', 'basebal', 'good', 'just', 'first', 'pitch', 'when', 'better', 'been', 'well', 'season', 'know', 'score', 'some', 'leagu', 'onli', 'were', 'look', 'their', 'start', 'dave', 'best', 'make', 'ball', 'base', 'mike', 'back', 'pitcher', 'much', 'come', 'other', 'then', 'point', 'gatech', 'becaus', 'even', 'after', 'could', 'give']

T4

['entri', 'output', 'defin', 'build', 'rule', 'remark', 'contest', 'check', 'onam', 'loop', 'stream', 'return', nan, 'year', 'obfusc', 'printf', 'char', 'part', 'quantize', 'ndet', 'fprintf', 'lilley', 'line', 'uuencod', 'file', 'echo', 'open', 'pars', 'ioccc', 'stderr', 'size', 'bhjn', 'count', 'winner', 'length', 'manchest', 'howel', 'judg', 'nrhj', 'author', 'endif', 'graem', 'prog', 'thex', 'here', 'cjpeg', 'indent', 'struct', 'toad', 'landon']

T5

['were', 'armenian', 'their', 'peopl', 'them', 'fire', 'said', 'turkish', 'when', 'been', 'then', 'kill', 'children', 'koresh', 'muslim', 'into', 'govern', 'start', 'know', 'armenia', 'turk', 'time', 'batf', 'could', 'come', 'turkey', 'after', 'over', 'didn', 'happen', 'first', 'burn', 'went', 'articl', 'some', 'other', 'waco', 'down', 'like', 'today', 'genocid', 'even', 'just', 'greek', 'those', 'soviet', 'live', 'becaus', 'onli', 'build']

T6

['some', 'articl', 'medic', 'effect', 'your', 'caus', 'like', 'food', 'diseas', 'doctor', 'which', 'patient', 'know', 'more', 'other', 'been', 'also', 'problem', 'time', 'peopl', 'studi', 'when', 'drug', 'just', 'veri', 'most', 'treatment', 'cancer', 'test', 'than', 'these', 'take', 'becaus', 'good', 'help', 'their', 'should', 'think', 'infect', 'onli', 'mani', 'gordon', 'pain', 'pitt', 'make', 'seem', 'much', 'such', 'after', 'year']

T7

['christian', 'your', 'believ', 'jesus', 'peopl', 'which', 'some', 'other', 'know', 'think', 'church', 'word', 'when', 'their', 'bibl', 'onli', 'mean', 'then', 'them', 'christ', 'were', 'faith', 'thing', 'becaus', 'love', 'just', 'time', 'more', 'like', 'question', 'also', 'life', 'exist', 'come', 'these', 'even', 'articl', 'those', 'make', 'read', 'religion', 'been', 'book', 'good', 'point', 'mani', 'paul', 'than', 'homosexu', 'lord']

T8

['peopl', 'your', 'right', 'articl', 'more', 'their', 'like', 'think', 'than', 'make', 'them', 'just', 'govern', 'other', 'state', 'some', 'want', 'should', 'onli', 'when', 'becaus', 'bill', 'well', 'good', 'thing', 'which', 'then', 'even', 'weapon', 'believ', 'were', 'those', 'crime', 'such', 'person', 'time', 'been', 'case', 'much', 'point', 'mean', 'know', 'these', 'mani', 'firearm', 'most', 'reason', 'take', 'drug', 'post']

T9

['file', 'imag', 'program', 'avail', 'graphic', 'mail', 'version', 'system', 'softwar', 'list', 'includ', 'format', 'your', 'user', 'also', 'data', 'other', 'inform', 'code', 'sourc', 'send', 'jpeg', 'post', 'which', 'site', 'packag', 'unix', 'support', 'server', 'comput', 'color', 'more', 'directori', 'some', 'anonym', 'line', 'email', 'name', 'copi', 'librari', 'look', 'convert', 'address', 'internet', 'machin', 'archiv', 'comp', 'info', 'base', 'request']

T10

['space', 'nasa', 'orbit', 'launch', 'earth', 'articl', 'mission', 'henri', 'some', 'like', 'moon', 'system', 'which', 'time', 'more', 'satellit', 'shuttl', 'other', 'year', 'also', 'planet', 'just', 'first', 'could', 'cost', 'project',

'than', 'into', 'scienc', 'high', 'toronto', 'were', 'data', 'work', 'solar', 'surfac', 'model', 'object', 'univers', 'point', 'develop', 'design', 'physic', 'flight', 'much', 'been', 'engin', 'observ', 'onli', 'star']

T11

['window', 'problem', 'when', 'work', 'know', 'program', 'display', 'some', 'manag', 'applic', 'font', 'which', 'your', 'screen', 'mous', 'then', 'think', 'error', 'want', 'like', 'microsoft', 'make', 'just', 'widget', 'chang', 'time', 'help', 'color', 'server', 'need', 'stephanopoulo', 'file', 'look', 'someth', 'onli', 'other', 'call', 'thank', 'question', 'articl', 'xterm', 'start', 'should', 'creat', 'into', 'memori', 'under', 'resourc', 'line', 'myer']

T12

['state', 'presid', 'year', 'nation', 'univers', 'report', 'inform', 'april', 'american', 'unit', 'research', 'group', 'number', 'health', 'public', 'been', 'their', 'servic', 'includ', 'which', 'program', 'page', 'confer', 'plan', 'were', 'washington', 'issu', 'also', 'offic', 'school', 'center', 'said', 'work', 'canada', 'meet', 'depart', 'other', 'organ', 'news', 'general', 'institut', 'press', 'provid', 'offici', 'time', 'week', 'fund', 'hous', 'member', 'last']

T13

['articl', 'like', 'just', 'when', 'your', 'more', 'engin', 'know', 'some', 'think', 'than', 'look', 'good', 'time', 'veri', 'thing', 'them', 'much', 'power', 'also', 'realli', 'drive', 'make', 'back', 'light', 'onli', 'should', 'want', 'here', 'other', 'work', 'which', 'wire', 'were', 'well', 'auto', 'ground', 'turn', 'then', 'year', 'problem', 'speed', 'been', 'even', 'into', 'most', 'where', 'down', 'take', 'could']

T14

['israel', 'articl', 'your', 'isra', 'arab', 'moral', 'peopl', 'right', 'their', 'some', 'think', 'which', 'other', 'were', 'just', 'islam', 'kill', 'jewish', 'onli', 'object', 'them', 'like', 'know', 'peac', 'more', 'state', 'such', 'when', 'than', 'fact', 'human', 'palestinian', 'muslim', 'becaus', 'keith', 'make', 'should', 'question', 'mani', 'attack', 'time', 'mean', 'then', 'even', 'said', 'opinion', 'could', 'been', 'these', 'those']

T15

['articl', 'your', 'some', 'like', 'which', 'other', 'peopl', 'know', 'were', 'more', 'when', 'just', 'their', 'think', 'them', 'time', 'onli', 'than', 'been', 'also', 'make', 'then', 'work', 'these', 'year', 'want', 'right', 'should', 'could', 'system', 'good', 'well', 'window', 'look', 'even', 'need', 'becaus', 'thing', 'problem', 'file', 'into', 'veri', 'most', 'mani', 'much', 'first', 'those', 'question', 'such', 'call']

Top words of topics obtained with hARTM model on the second hierarchical level for 20 Newsgroups dataset (the number of topics on the first hierarchical level is set to one).

T1

['encrypt', 'chip', 'secur', 'clipper', 'govern', 'phone', 'system', 'other', 'which', 'your', 'public', 'some', 'algorithm', 'articl', 'more', 'messag', 'escrow', 'could', 'privaci', 'inform', 'number', 'need', 'know', 'like', 'make', 'secret', 'technolog', 'their', 'been', 'even', 'netcom', 'protect', 'data', 'than', 'agenc', 'just', 'enforc', 'access', 'time', 'comput', 'such', 'peopl', 'communic', 'david', 'devic', 'them', 'wiretap', 'then', 'work', 'crypto']

T2

['drive', 'card', 'know', 'thank', 'anyon', 'work', 'problem', 'like', 'need', 'driver', 'just', 'appl', 'articl', 'system', 'which', 'disk', 'comput', 'monitor', 'some', 'plea', 'help', 'scsi', 'video', 'want', 'when', 'also', 'machin', 'price', 'board', 'good', 'look', 'more', 'could', 'control', 'them', 'your', 'hard', 'time', 'than', 'memori', 'think', 'sound', 'other', 'speed', 'power', 'appreci', 'make', 'these', 'question', 'info']

T3

['game', 'year', 'team', 'articl', 'play', 'player', 'last', 'more', 'think', 'like', 'time', 'than', 'baseb', 'good', 'first', 'just', 'pitch', 'when', 'better', 'been', 'season', 'well', 'know', 'score', 'leagu', 'some', 'dave', 'look', 'were', 'their', 'mike', 'start', 'best', 'ball', 'make', 'base', 'back', 'pitcher', 'over', 'much', 'come', 'other', 'point', 'could', 'then', 'after', 'give', 'gatech', 'even', 'becaus']

T4

['jfif', 'libxmu', 'part', 'ndet', 'lilley', 'georg', 'bhjn', 'howel', 'manchest', 'nrhj', 'here', 'file', 'rtrace', 'some', 'cjpeg', 'pmfq', 'waii', 'acker', 'bhjm', 'unizh', 'kouhia', 'bxlt', 'juhana', 'scodal', 'wwiz', 'gizw', '

undeclar', 'salmon', 'roman', 'begin', 'cellar', 'prof', 'zbhj', 'mbxn', 'mitchel', 'gifconvert', 'jpegview', 'pictpixi', 'western', 'cliff', 'radius', 'bizm', 'doubl', 'deniaud', 'pnei', 'suresh', 'ampr', 'referenc', 'huff man', 'sphere']

T5

['were', 'armenian', 'their', 'peopl', 'them', 'said', 'fire', 'when', 'been', 'turkish', 'then', 'kill', 'children' , 'govern', 'koresh', 'know', 'into', 'muslim', 'start', 'time', 'armenia', 'turk', 'could', 'come', 'batf', 'hap pen', 'after', 'didn', 'over', 'articl', 'first', 'some', 'turkey', 'burn', 'like', 'went', 'other', 'waco', 'down', ' today', 'just', 'even', 'genocid', 'those', 'greek', 'becaus', 'live', 'soviet', 'right', 'never']

T6

['some', 'articl', 'medic', 'effect', 'caus', 'food', 'your', 'disea', 'which', 'like', 'doctor', 'patient', 'other', 'more', 'been', 'know', 'problem', 'also', 'drug', 'time', 'studi', 'when', 'peopl', 'veri', 'most', 'treatment ', 'than', 'these', 'cancer', 'test', 'health', 'just', 'their', 'were', 'help', 'should', 'take', 'becaus', 'good', 'i nfect', 'year', 'gordon', 'mani', 'pain', 'pitt', 'experi', 'case', 'after', 'such', 'think']

T7

['christian', 'your', 'believ', 'jesus', 'peopl', 'which', 'some', 'other', 'know', 'think', 'church', 'word', ' mean', 'when', 'their', 'bibl', 'then', 'them', 'thing', 'christ', 'were', 'becaus', 'just', 'exist', 'faith', 'more' , 'love', 'like', 'question', 'time', 'life', 'also', 'come', 'articl', 'these', 'even', 'make', 'religion', 'those', ' read', 'been', 'point', 'good', 'than', 'mani', 'reason', 'person', 'paul', 'book', 'homosexu']

T8

['peopl', 'your', 'right', 'articl', 'more', 'their', 'think', 'like', 'than', 'make', 'govern', 'state', 'them', 'oth er', 'just', 'some', 'want', 'should', 'bill', 'well', 'becaus', 'when', 'which', 'thing', 'good', 'weapon', 'tho se', 'then', 'believ', 'were', 'even', 'know', 'such', 'time', 'crime', 'been', 'case', 'mean', 'person', 'these' , 'point', 'much', 'mani', 'money', 'most', 'firearm', 'control', 'clinton', 'take', 'reason']

T9

['file', 'imag', 'mail', 'graphic', 'avail', 'program', 'softwar', 'list', 'system', 'format', 'version', 'user', ' post', 'data', 'also', 'inform', 'includ', 'send', 'other', 'comput', 'your', 'packag', 'jpeg', 'email', 'site', 'a ddress', 'plea', 'code', 'some', 'sourc', 'color', 'which', 'look', 'internet', 'anonym', 'more', 'unix', 'sup port', 'find', 'copi', 'group', 'free', 'convert', 'line', 'info', 'archiv', 'need', 'comp', 'tool', 'help']

T10

['space', 'nasa', 'orbit', 'launch', 'articl', 'earth', 'some', 'henri', 'mission', 'like', 'which', 'moon', 'syste m', 'more', 'time', 'satellit', 'shuttl', 'other', 'just', 'planet', 'than', 'first', 'year', 'also', 'cost', 'into', 'cou ld', 'high', 'project', 'were', 'toronto', 'work', 'solar', 'design', 'power', 'much', 'develop', 'scienc', 'ph ysic', 'data', 'observ', 'flight', 'make', 'light', 'been', 'veri', 'long', 'spacecraft', 'these', 'object']

T11

['window', 'file', 'program', 'problem', 'your', 'when', 'applic', 'display', 'work', 'font', 'server', 'mana g', 'which', 'mous', 'widget', 'some', 'microsoft', 'error', 'screen', 'motif', 'like', 'know', 'line', 'chang', 'then', 'make', 'help', 'need', 'stephanopoulo', 'entri', 'other', 'color', 'includ', 'should', 'client', 'want', 'system', 'look', 'time', 'open', 'call', 'xterm', 'resourc', 'under', 'start', 'support', 'just', 'function', 'defi n', 'also']

T12

['univ', 'state', 'presid', 'nation', 'year', 'inform', 'unit', 'april', 'book', 'report', 'american', 'research', ' group', 'page', 'work', 'includ', 'public', 'confer', 'offic', 'number', 'press', 'school', 'program', 'been', 'center', 'washington', 'also', 'general', 'publish', 'york', 'organ', 'depart', 'which', 'meet', 'issu', 'plan', 'institut', 'servic', 'their', 'offici', 'world', 'canada', 'educ', 'member', 'present', 'week', 'said', 'last', 'ti me', 'intern']

T13

['articl', 'like', 'just', 'your', 'when', 'know', 'think', 'look', 'engin', 'good', 'more', 'some', 'thing', 'time ', 'than', 'veri', 'them', 'realli', 'much', 'make', 'also', 'want', 'back', 'drive', 'here', 'well', 'work', 'then', 'were', 'year', 'been', 'uiuc', 'auto', 'problem', 'other', 'even', 'take', 'which', 'could', 'should', 'speed', ' light', 'turn', 'around', 'anyon', 'down', 'becaus', 'price', 'chang', 'into']

T14

['israel', 'articl', 'isra', 'arab', 'your', 'moral', 'right', 'peopl', 'their', 'some', 'which', 'other', 'think', 'w ere', 'islam', 'jewish', 'kill', 'just', 'object', 'peac', 'them', 'state', 'know', 'like', 'such', 'when', 'muslim' , 'fact', 'human', 'more', 'palestinian', 'than', 'keith', 'should', 'attack', 'make', 'becaus', 'mani', 'time', 'question', 'opinion', 'even', 'countri', 'said', 'been', 'those', 'these', 'mean', 'most', 'could']

T15

['articl', 'your', 'some', 'like', 'which', 'other', 'peopl', 'know', 'were', 'more', 'when', 'just', 'their', 'thi nk', 'them', 'time', 'than', 'been', 'also', 'make', 'then', 'work', 'these', 'year', 'want', 'right', 'should', 'c ould', 'system', 'good', 'well', 'window', 'look', 'even', 'need', 'becaus', 'thing', 'problem', 'file', 'into', 'veri', 'most', 'mani', 'much', 'first', 'those', 'question', 'such', 'call', 'state']

Top words of topics obtained with hARTM model on the first hierarchical level for balanced WoS dataset (pre-processing 1).

T1_lev1

['enthesi', 'residu', 'manifold', 'reducta', 'sort', 'meanwhil', 'tailor', 'kinet', 'amyotroph', 'retain', 'ques tion', 'greater', 'input', 'nacl', 'dimens', 'cassett', 'rich', 'anoth', 'enlarg', 'tetracyclin', 'extensor', 'near bi', 'adopt', 'arteriol', 'match', 'cross', 'hydrogel', 'fluoxetin', 'tongu', 'loui', 'cari', 'shape', 'lactoferrin' , 'coactiv', 'insuffici', 'note', 'pharynx', 'daili', 'pronounc', 'fifth', 'octam', 'fourteen', 'histocompat', 'a nkk', 'portend', '4255', 'formalin', 'depict', 'epistasi', 'obstacl']

T2_lev1

['profil', 'microtomographi', 'photo', 'theophyllin', 'hydroxid', 'formul', 'elong', 'hyper', 'greec', 'inte nsifi', 'endur', 'verlag', 'unlock', 'coverag', 'subsystem', 'indo', 'flourish', 'southeastern', 'overlook', ' aberr', 'iagr', 'american', 'tall', 'april', 'side', 'satisfi', 'evolut', 'migrat', 'document', 'striat', 'enough', ' fraction', 'reconcil', 'seen', 'inva', 'vehicl', 'friend', 'concept', 'brazilian', 'dentin', 'slow', 'settl', 'ampl itud', 'equal', 'zhejiang', 'arbuscular', 'subsid', 'mississippi', 'chromobacterium', 'schedul']

T3_lev1

['deter', 'drive', 'ciliat', 'controversi', 'enceph', 'solidifi', 'elicitor', 'fasebj', 'stabli', 'cocktail', 'faseb', ' cada', 'ainsi', 'robusta', 'retain', 'forelimb', 'societi', 'transloc', 'michael', 'peptidoglycan', 'sing', 'alga ', 'artifici', 'undifferenti', 'mileston', 'abcc', 'attract', 'habit', 'rebirth', 'wiley', 'doubl', 'mutagenesi', 'o verwint', 'delta', 'cytotox', 'valley', 'serious', 'strongest', 'plasmopara', 'crew', 'para', 'vast', 'salvag', ' pearl', 'asphyxi', 'angstrom', 'subcellular', 'aminoacid', 'conclusionth', 'cornel']

T4_lev1

['cauchi', 'claim', 'detrend', 'miner', 'glcm', 'keypoint', 'narrowband', 'endmemb', 'radarsat', 'momen tum', 'landscap', 'freedom', 'irrev', 'ascend', 'qsar', 'multiparamet', 'colloc', 'stego', 'ascertain', 'alwa y', 'nadir', 'lengthen', 'penetr', 'centr', 'despit', 'automata', 'prospect', 'insula', 'widen', 'springer', 'sub urb', 'document', 'unlik', 'raster', 'leach', 'compart', 'continuum', 'notabl', 'arrow', 'diverg', 'disagr', 'i nerti', 'mmse', 'version', 'mitosi', 'bluefin', 'fibril', 'would', 'signatur', 'fault']

T5_lev1

['opinion', 'isol', 'full', 'sydney', 'build', 'sale', 'negoti', 'devianc', '7030', 'stimul', 'classic', 'appli', 'hy pothalamus', 'mole', 'straightforward', 'primat', 'schmidt', 'prosper', 'exampl', 'submiss', 'shed', 'brin g', 'anecdot', 'antagonist', 'berri', 'tracker', 'liken', 'unsolv', 'heavier', 'parasympathet', 'exagg', 'gene rous', 'punit', 'homophob', 'pleasant', 'homo', 'breath', 'overlay', 'rape', 'collap', 'backlash', 'bought', 'conclusionsit', 'prompt', 'truli', 'delay', 'prize', 'underscor', 'decod', 'ordinarili']

T6_lev1

['rhythm', 'immunogenet', 'christian', 'atherosclerosi', 'quantifi', 'complement', 'cutoff', 'radiographi ', 'anorexigen', 'classic', 'canadian', 'ascia', 'rhipicephalus', 'thereof', 'longest', 'edema', 'insuffici', 's ociodemograph', 'exist', 'anticholinerg', 'nocicept', 'heal', 'auto', 'cuff', 'degen', 'middl', 'commerci', 'noncontinu', 'hydrazin', 'electrocardiogram', 'qrtpcr', 'proband', 'eleven', 'timepoint', 'tertiari', 'geni tourinari', '8144', 'chromat', 'sustain', 'ngrise', 'urem', 'haplogroup', 'taqman', 'kaplan', 'order', 'bron chiectasi', 'anorexia', 'exhibit', 'ipsilat', 'austria']

T7_lev1
['whole', 'fluidic', 'axisymmetr', 'disintegr', 'orif', 'bulg', 'fluidiz', 'ultrasound', 'budget', 'assist', 'polish', 'streamlin', 'automot', 'sudden', 'implicit', 'spin', 'predetermin', 'aircraft', 'stepwi', 'sigma', 'specifi', 'pick', 'alter', 'outsid', 'grow', 'conduc', 'band', 'ident', 'quantiti', 'travel', 'white', 'laid', 'calcium', 'isol', 'quantifil', 'copyright', 'pertain', 'guidelin', 'stock', '2819', 'bottleneck', 'stripe', 'rail', 'anoth', 'debat', 'afflict', 'gradual', 'austrian', 'permafrost', 'sign']
T8_lev1
['testifi', 'ordinarili', 'organiz', 'craft', 'conform', 'cash', 'sick', 'apparel', 'eavesdrop', 'swap', 'vehicular', 'autonomi', 'socket', 'petri', 'wellknown', 'geiger', 'launch', 'broad', 'snippet', 'stamp', 'enrich', 'endless', 'pose', 'irish', 'auckland', 'petroleum', 'incomplet', 'sector', 'invent', 'overcrowd', 'playstat', 'spark', 'replic', 'unconv', 'microcomput', 'immedi', 'discourag', 'michael', 'increment', 'caught', 'overwhelm', 'thermostat', 'causal', 'percentag', 'fault', 'triangul', 'bilinear', 'abnorm', 'anom', 'relativist']
T9_lev1
['causal', 'marriag', 'anytim', 'detent', 'manipul', 'incept', 'spectacular', 'mead', 'parallel', 'metrolog', 'montpelli', 'short', 'later', 'media', 'pioneer', 'airflow', 'likert', 'viewpoint', 'imposs', 'ophthalmologist', 'premi', 'visit', 'televi', 'ascertain', 'lost', 'injuri', 'famili', 'cctv', 'chemic', 'never', 'manifest', 'quot', 'backbon', 'certifi', 'thorn', 'talk', 'pure', 'swot', 'possess', 'caesar', 'thermodynam', 'ballist', 'hayfev', 'burgess', 'borrow', 'colleagu', 'spend', 'phonolog', 'extern', 'alon']
T10_lev1
['polar', 'elucid', 'projector', 'propul', 'extrus', 'simpler', 'signer', 'denoi', 'isogeometr', 'starter', 'crane', 'markovian', 'long', 'reflect', 'substat', 'unravel', 'parasit', 'budget', 'fashion', 'root', 'reflector', 'indistinguish', 'compromi', 'constel', 'behind', 'telescop', 'distributor', 'leaki', 'wound', 'oven', 'metrolog', 'larger', 'bright', 'project', 'invalid', 'salient', 'inevit', 'node', 'notwithstand', 'ascend', 'corpor', 'nanosecond', 'expon', 'automat', 'suspen', 'wise', 'enough', 'consolid', 'weaker', 'stego']

Top words of topics obtained with hARTM model on the second hierarchical level for balanced WoS dataset (pre-processing 1).

T1_lev2
['adjust', 'unexpect', 'illumin', 'sucro', 'dual', 'enterocyt', 'isomera', 'apart', 'agricultur', 'ripe', 'forti', 'aliphat', 'methyltransfera', 'window', 'dogma', 'polypeptid', 'ftir', 'washout', 'biotechnolog', 'pylon', 'bare', 'deltamethrin', 'polystyren', 'strawberri', 'ailment', 'eleven', 'prolin', 'preferenti', 'german', 'grew', '6420', 'commiss', 'fail', 'lipa', 'presum', 'find', 'last', 'catala', 'deal', 'core', 'cheaper', 'labor', 'readi', 'kind', 'resembl', 'straightforward', 'organophosphorus', 'polar', 'pilot', 'dinucleotid']
T2_lev2
['xupv', 'canadian', 'reconnaiss', 'exploit', 'spirali', 'prevail', 'reanali', 'spicatum', 'orient', 'minimum', 'merit', 'tandem', 'strata', 'clean', 'evidenti', 'embay', 'fluvial', 'hidden', 'polarimetri', 'perc', 'ikono', 'burullus', 'nonclimat', 'cokrig', 'brillant', 'cuprit', 'radiant', 'patagonian', 'rojo', 'aviri', 'goci', 'meri', 'nadir', 'georeferenc', 'mesic', 'savart', 'superfacet', 'tien', 'semiempir', 'cosimul', 'dinosaurian', 'polariscop', 'medicaid', 'pzga', 'horus', 'tetrachloroethylen', 'palsar', 'penrith', 'rsscs', 'wtli']
T3_lev2
['shelter', 'clarif', 'flaw', 'begin', 'destruct', 'poor', 'fiction', 'suffici', 'fiscal', 'sent', 'albeit', 'small', 'dilemma', 'delhi', 'teen', 'obscur', 'eighth', 'multidimens', 'sociopolit', 'administ', 'irrespect', 'exert', 'medium', 'sydney', 'uygur', 'prevail', 'wrong', 'taboo', 'underlin', 'overlap', 'turk', '7189', 'period', 'judgment', 'mock', 'transcript', 'superfici', 'necessari', 'gave', 'alongsid', 'unstabl', 'side', 'discov', 'legisl', 'club', 'rise', 'complianc', 'establish', 'provok', 'abus']
T4_lev2
['appli', 'hydroxybutyr', 'focus', 'everi', 'postul', 'bilat', 'gradual', 'autist', 'part', 'counteract', 'deserv', 'natriuret', 'tortuo', 'obvious', 'nascent', 'complaint', 'longer', 'almost', 'fibrosi', 'transmitr', 'debat', 'patch', 'proven', 'suspicion', 'discomfort', 'coordin', 'shortcom', 'rodent', 'live', 'higher', 'brown', 'inter

depend', 'nativ', 'ascertain', 'conven', 'chromosom', 'furthermor', 'enteropathi', 'cognit', 'kappa', 'reli anc', 'seek', 'true', 'volumetri', 'pathomechan', 'object', 'themselv', 'prescrib', 'mendelian', 'monoami n']

T5_lev2

['press', 'respon', 'bayesian', 'wherein', 'notic', 'correct', 'homogen', 'offen', 'purpo', 'vhdl', 'insuffici' , 'asymmetri', 'awaken', 'histor', 'nonzero', 'adverti', 'multitask', 'endpoint', 'coupl', 'readabl', 'evolv' , 'insid', 'inva', 'search', 'intersect', 'crime', 'otherwi', 'serbia', 'spent', 'dedic', '7987', 'vanet', 'closer', 'grasp', 'confront', 'nonetheless', 'pseudo', 'trend', 'drastic', 'batch', 'redefin', 'backup', 'contrari', 'leg aci', 'multius', 'chart', 'inher', 'reject', 'exit', 'stop']

T6_lev2

['defect', 'generic', 'larger', 'closest', 'delaunay', 'insensit', 'pictur', 'omit', 'resort', 'logist', 'sole', 'hyp er', 'enter', 'duplic', 'squar', 'resid', 'kingdom', 'biom', 'movi', 'basca', 'lstm', 'daubechi', 'hopcncc', 'c anni', 'grayscal', 'libflasm', 'hevc', 'ksrc', 'gepsvm', 'semisupervi', 'microblog', 'backpropag', 'jpeg', ' spot', 'biometr', 'reveal', 'retrain', 'declar', 'abid', 'taxonomi', 'minimi', 'thorough', 'exud', 'librari', 'k nowledg', 'critic', 'hough', 'heteroscedast', 'test', 'prohibit']

T7_lev2

['eapp', 'mall', 'assimil', 'transfer', 'stori', 'quantif', 'restructur', 'renew', 'livestock', 'bring', 'take', 'tra deoff', 'abund', 'guarant', 'holi', 'destin', 'conveni', 'zero', 'hundr', 'horizon', 'exampl', 'physic', 'effor t', 'concert', '4998', 'hygien', 'serious', 'clear', 'artifici', 'reliev', 'smaller', 'technician', 'togeth', 'outso urc', 'februari', 'primarili', 'campaign', 'gave', 'shift', 'inexpen', 'arbitrari', 'ethiopian', 'exterior', 'poll utograph', 'cook', 'groyn', 'carrot', 'accompani', 'hellen', 'opposit']

T8_lev2

['climact', 'prece', 'late', 'handicap', 'belong', 'benzo', 'therebi', 'nonspecif', 'frame', 'worksheet', 'har vard', 'neighborhood', 'director', 'necessari', 'sensit', 'stimul', 'riski', 'size', 'otherwi', 'shame', 'bedro om', 'unitari', 'relap', 'alloimmun', 'typolog', 'ancova', 'vocat', 'multic', 'discomfort', 'multilevel', 'co ncept', 'valuabl', 'marit', 'academ', '6321', '5307', 'dutch', 'phenomenon', 'brazilian', 'reliabl', 'royal', 'caution', 'civic', 'intend', 'california', 'indoor', 'prompt', 'adopt', 'exclud', 'discussionit']

T9_lev2

['midlin', 'restraint', 'justifi', 'resum', 'likewi', 'disagr', 'classic', 'novemb', 'sost', '8592', 'french', 'cer clag', 'ever', 'public', 'prematur', 'undoubt', 'placement', 'trabecular', 'tailor', 'japan', 'satisfactori', 'lo osen', 'spite', 'salin', 'weak', 'urinari', 'cataract', 'femor', 'ltda', 'editora', 'commenc', 'cyclosporin', 'i mmobil', 'explain', 'contributori', 'longterm', 'interrupt', 'basri', 'transit', 'arthropathi', 'bcss', 'tertil', ' ochronosi', 'osteomalacia', 'nonbiolog', 'btcp', 'nssa', 'nongarden', 'stoke', 'multimorbid']

T10_lev2

['happen', 'intermitt', 'combin', 'histopatholog', 'hour', 'flank', 'even', 'food', 'tannin', 'focuss', 'azim uth', 'legionella', 'foundat', 'smallhold', 'spoil', 'chlorid', 'prey', 'notic', 'greatest', 'safer', 'realiti', 'po ol', 'burden', 'mangan', 'bigger', 'legal', 'rift', 'inventori', 'heterogen', 'compost', 'gase', 'implement', ' anisaki', 'exhaust', 'overnight', 'exhibit', 'rainbow', 'northward', 'substitut', 'unlik', 'argentina', 'enter ococcus', 'hectar', 'elev', 'plant', 'desorpt', 'provis', 'mediterranean', 'ncnd', 'bioinspir']

T11_lev2

['everi', 'multipli', 'hard', 'chow', 'hump', 'simpli', 'breakwat', 'configur', 'rich', 'admiss', 'penal', 'abs ent', 'hhhc', 'snapshot', 'ieee', 'backward', 'distributor', 'titl', 'decay', 'provi', '7400', 'dimensionless', 'come', 'increment', 'semiparametr', 'nonsmooth', 'characteri', 'interconnect', 'breakdown', 'calibr', ' descent', 'queen', 'colli', 'alya', 'traceless', 'aosvr', 'isoc', 'cocycl', 'tomo', 'integro', 'steinmetz', 'stenc il', 'monomi', 'inviscid', 'diffeomorph', 'tmdl', 'chapman', 'petiga', 'lexicograph', 'belfast']

T12_lev2

['english', 'extra', 'lithium', 'primat', 'fake', 'terminolog', 'equal', 'intrana', 'irrespect', 'intimaci', 'russ el', 'augment', 'protagonist', 'phonolog', 'multifacet', 'futur', 'friend', 'easi', 'volleyb', 'glrlm', 'eoss', ' nonoffend', 'huasmc', 'choreograph', 'hubrist', 'pacc', 'unimod', 'phonet', 'excerpt', 'ammi', 'weird', ' unconfid', 'joseph', 'lifelik', 'chemosensori', 'goosebump', 'eerland', 'courtroom', 'allopar', 'choroide

raemia', 'nocoa', 'pcsk', 'ngldm', 'gesticul', 'deafblind', 'adaptor', 'feit', 'autofluoresc', 'etiquett', 'index']

T13_lev2

['readabl', 'thank', 'chameleon', 'supplementari', 'launch', 'caus', 'within', 'explicit', 'arbitrarili', 'exhibit', 'overhaul', 'neither', 'subgroup', 'over', 'center', 'submiss', 'trivial', 'densiti', 'faster', 'simpler', 'fade', 'unduli', 'burden', '4810', 'fragment', 'compat', 'relativist', 'everi', 'runtim', '5093', 'huge', 'basic', 'evad', 'digest', 'haswel', 'offlin', 'transport', 'readili', 'unavail', 'permiss', 'heidelberg', 'imper', 'irregular', 'merchant', 'fetch', 'lookup', 'tight', 'crosstalk', 'behaviour', 'incoh']

T14_lev2

['zigzag', 'novelti', 'benefici', 'departur', 'molten', 'command', 'parallel', 'investor', 'wise', 'satisfactori', 'indirect', 'injector', 'pipe', 'like', 'inward', 'fluorid', 'meant', 'explain', 'rout', 'unaffect', 'breakout', 'straw', 'poison', 'heav', 'bender', 'neutral', 'berkeley', 'frame', 'bodi', 'carlo', 'cermet', 'mppt', 'rather', 'cobra', 'push', 'ccpp', 'vnand', 'wccb', 'greifswald', 'heatlin', 'ecoscarc', 'planck', 'igfem', 'discriminacion', 'olgw', 'flinak', 'catholyt', 'zahedan', 'flammabl', 'direkt']

T15_lev2

['legaci', 'mine', 'elect', 'satisfactori', 'weak', 'drywal', 'math', 'undertak', 'contributor', 'subsequ', 'tend', 'border', 'fact', 'kaplan', 'assum', 'resolv', 'para', 'substitut', 'central', 'adequ', 'accel', 'agil', 'humanist', 'penetr', 'fellow', 'gearbox', 'biggest', 'transmiss', 'date', 'generat', 'tech', 'disciplinari', 'impart', 'drastic', 'accompani', 'campaign', 'brought', 'convey', 'soccer', 'observ', 'chamber', 'turkey', 'constitu', 'alloc', 'steel', 'grasp', 'georgia', 'clarifi', 'communic', 'except']

T16_lev2

['tropic', 'delimit', 'degr', 'absent', 'broad', 'modul', 'critic', 'hard', 'best', 'fingerprint', 'contemporari', 'linnaeus', 'version', 'genomewid', 'primit', 'attribut', 'suffici', 'secondari', 'abalon', 'rearrang', 'booster', 'fair', 'suitabl', 'perturb', 'earlier', 'competit', 'gross', 'oncolog', 'synergist', 'ubiquit', 'devast', 'constraint', 'highlight', 'acquir', 'simultan', 'repressor', 'invertebr', 'spruce', 'mutagenesi', 'threat', 'retriev', 'serv', 'search', 'circul', 'landscap', 'therebi', 'note', 'mainland', 'anthropolog', 'retin']

T17_lev2

['handl', 'resolv', 'control', 'glean', 'stolen', 'forget', 'preliminari', 'relianc', 'immin', 'ball', 'decept', 'slowli', 'secondari', 'holist', 'omit', 'physiolog', 'prevent', 'suit', 'blend', 'legitim', 'elimin', 'owner', 'momentari', 'visuospati', 'answer', 'redress', 'uncov', 'unaffect', 'grow', 'propel', 'theoriz', 'said', '6481', 'lone', 'nonsoci', 'salienc', 'bayesian', 'expan', 'simpl', 'pleasur', 'debilit', 'consensu', 'harder', 'turn', 'warmer', 'press', 'inflat', 'late', 'somewhat', 'haxbi']

T18_lev2

['insid', 'ipsilat', 'locali', 'length', 'appendag', 'astilbin', 'mincl', 'erdr', 'pdzk', 'pgam', 'liga', 'ectoderm', 'abdsc', 'oxldl', 'simvastatin', 'geldanamycin', 'cfme', 'fifibroblast', 'fgcs', 'ampar', 'importin', 'daratumumab', 'ryanodin', 'adap', 'oculoplast', 'tslp', 'swdi', 'mecp', 'geminin', 'proapoptot', 'adaptor', 'osteocyt', 'lsds', 'lysin', 'epileptiform', 'resveratrol', 'huvec', 'umbil', 'nannochloropsi', 'gliotransmiss', 'cmepa', 'valsartan', 'planarian', 'cgns', 'emigr', 'buch', 'butachlor', 'mastocytosi', 'trypanosom', 'trpm']

T19_lev2

['intend', 'inaccur', 'infus', 'dsms', 'delimit', 'nexus', 'swedish', 'code', 'contractor', 'endeavor', 'spanish', 'consist', 'charact', 'larger', 'differ', 'indian', 'saeta', 'dhts', 'icaro', 'slovak', 'sidi', 'bouzid', 'incentivi', 'archiv', 'divid', 'juli', 'embrac', 'behind', 'indo', 'focal', 'purcha', 'catastroph', 'unstructur', 'netherland', 'undesir', 'generali', 'said', 'guarant', 'script', 'queri', 'illustr', 'elicit', 'merchant', 'bibliograph', 'springboard', 'hurdl', 'theori', 'recruit', 'either', 'imperfect']

T20_lev2

['slowli', 'inabl', 'manageri', 'characteri', 'bolster', 'appar', 'masteri', 'nest', 'remain', 'delay', 'ground', 'underscor', 'focuss', 'immediaci', 'whole', 'deriv', 'gather', 'schizotypi', 'build', 'delhi', 'surplus', 'bias', 'garner', 'temporari', 'monkey', 'longer', 'equilibria', 'sign', 'modest', 'gradual', 'free', 'tell', 'teen', '

tradeoff', 'anger', 'coder', 'macaqu', 'interven', 'loyalti', 'half', 'light', 'relianc', 'depict', 'extant', 'aggreg', 'fictiti', 'recreat', 'strengthen', 'contend', 'vantag']

T21_lev2

['downward', 'agreement', 'know', 'exampl', 'fell', 'germani', 'warp', 'track', 'possibl', 'restrict', 'suffici', 'appreci', 'sole', 'fail', 'absenc', 'seab', 'contrari', 'precondit', 'assum', 'increment', 'latch', 'mitig', 'exceed', 'float', 'tight', 'powder', 'trademark', 'undertaken', 'inflex', 'overlap', 'sometim', 'constitu', 'leach', 'prefabr', 'mechanist', 'intact', 'tide', 'consequ', 'tend', 'despit', 'groyn', 'panel', 'insert', 'narrow', 'index', 'preserv', 'geometr', 'undermin', 'dispo', 'machineri']

T22_lev2

['minor', 'whisker', 'metallurgi', 'biohpp', 'excavatum', 'trueness', 'pectus', 'materialia', 'polycryst', 'laterotrus', 'inch', 'zenotec', 'aiscn', 'sbfem', 'lscm', 'tetragon', 'hvof', 'mcat', 'knitwear', 'cenosph', 'lipus', 'crumb', 'stair', 'piezo', 'afmn', 'orthosi', 'patternmak', 'ossicular', 'hmsc', 'edentul', 'ctrlbrace', 'malocclus', 'sapphir', 'periap', 'acta', 'sandblast', 'hcfs', 'microleakag', 'igzo', 'surgimap', 'archwir', 'extrud', 'dlms', 'phemt', 'ointment', 'subneighborhood', 'chondrocyt', 'stearat', 'zahnfabrik', 'roseoflavin']

T23_lev2

['vectorp', 'elabor', 'cope', 'area', 'converg', 'sever', 'beamform', 'admitt', 'prng', 'intrin', 'accomplish', 'unseen', 'multipli', 'stick', 'occurr', 'gmbh', 'pulsat', 'despit', 'setup', 'interconnect', 'adver', 'sampler', 'bode', 'changeabl', 'inferior', 'petroleum', 'offic', 'faster', 'inter', 'could', 'cnns', 'strict', 'generali', 'broader', 'waveguid', 'decay', 'happen', 'assign', 'fade', 'shrink', 'return', 'cutoff', 'bicmo', 'quit', 'spmsg', 'almost', 'rise', 'content', 'jacobian', 'induc']

T24_lev2

['stir', 'close', 'gaval', 'cuticl', 'aaval', 'secular', 'greatest', 'anoth', 'becom', 'last', 'trend', 'absenc', 'nevertheless', 'manifest', 'insid', 'amongst', 'shown', 'analyt', 'make', 'horizon', 'monitor', 'carolina', 'uncorrel', 'approxim', 'primari', 'geostatist', 'techniqu', 'resid', 'impact', 'french', 'version', 'constant', 'downward', 'stabil', 'prospect', 'often', 'logarithm', 'partit', 'sensibl', 'still', 'year', 'cutoff', 'closer', 'compil', 'modest', 'hamilton', 'sought', 'endolymph', 'csnn', 'venturi']

T25_lev2

['affect', 'depict', 'compar', 'return', 'satisfactori', 'destruct', 'light', 'trend', 'window', 'follow', 'railway', 'amend', 'blisk', 'biochip', 'disconnector', 'tcam', 'fasten', 'chassi', 'consol', 'foundat', 'exceed', 'mutual', 'efficaci', 'compromi', 'competit', 'realtim', 'today', 'crash', 'field', 'matur', 'bridg', 'biconductor', 'diagram', 'downtim', 'written', 'finish', 'streamlin', 'adiabat', 'decrea', 'within', 'devel', 'alreadi', 'articl', 'mean', 'appropri', 'uncontrol', 'preload', 'enorm', '9946', 'known']

T26_lev2

['movement', 'ambigu', 'onto', 'poster', 'render', 'fluenci', 'scanner', 'heighten', 'deriv', 'comorbid', 'behav', 'otherwi', 'pyramid', 'atlas', 'contributor', 'dual', 'semi', 'exotherm', 'avenu', 'elus', 'feedback', 'verifi', 'uptak', 'satisfi', 'shorter', 'cxcr', 'butyr', 'parkinson', 'length', 'induct', 'cord', 'broad', 'lifespan', 'disinhibit', '8859', 'fifti', 'made', 'disorgan', 'anesthesia', 'encompass', 'ssri', 'strongest', 'appropri', 'leucin', 'told', 'hallucin', 'spent', 'longer', 'avoid', '2416']

T27_lev2

['earlier', 'obvious', 'immunocompromi', 'coat', 'commentari', 'testi', 'behalf', 'aspergillus', 'prolin', 'evidenc', 'million', 'francisellosi', 'polym', 'malnutrit', 'bioassay', 'systemat', 'underpin', 'green', 'desir', 'catalyt', 'compo', 'hinder', 'spike', 'crystallograph', 'chemistri', 'enorm', 'gpcr', 'milieu', 'solid', 'polar', 'ifng', 'moieti', 'frog', 'unprec', 'uncontrol', 'orientali', 'casino', 'mitot', 'initio', 'straightforward', 'fresh', 'mammal', 'compromi', 'effort', 'immen', 'enlarg', 'preferenti', 'mortem', 'occupi', 'publish']

T28_lev2

['colostrum', 'microperfus', 'apolipoprotein', 'multiplex', '11498', 'congen', 'prepar', 'cardiometabol', 'prognost', 'belong', 'lesion', 'streptococcus', 'noninva', 'confound', 'infrequ', 'necessari', 'conver', 'klebsiella', 'forearm', 'vapour', 'hand', 'dawley', 'spragu', 'spite', 'resultsth', '9799', 'bacteria', 'landmark', 'iranian', 'unchang', 'filament', 'ioniz', 'tubul', 'proceed', 'work', 'depot', 'visit', 'accreta', 'compr

i', 'malondialdehyd', 'ergomet', 'spontan', 'barcelona', 'averag', 'angiogen', 'greek', 'mutual', 'cresol', 'severinghaus', 'sought']
T29_lev2
['intermedi', 'true', 'seri', 'manag', 'identifi', 'comprehend', 'chosen', 'familiari', 'breath', 'merg', 'special', 'summari', 'seek', 'empha', 'list', 'highlight', 'coach', 'ascertain', 'notion', 'easi', 'effort', 'commut', 'obtrus', 'foss', 'unusu', 'distinct', 'configur', 'countless', 'except', 'biom', 'knowledg', 'viabil', 'click', 'riski', 'button', 'entir', 'regardless', 'icon', 'viewpoint', 'shall', 'alert', 'newer', 'workload', 'tourist', 'keyword', 'street', 'necessit', 'watch', 'ankl', 'millisecond']
T30_lev2
['tropic', 'influenti', 'effect', 'keep', 'ermion', 'necessit', 'imperfect', 'davi', 'follow', 'surg', 'faith', 'never', 'resurg', '2555', 'inquiri', 'deepen', 'scarc', 'alongsid', 'blow', 'prepar', 'outcom', 'happen', 'constant', 'doubt', 'showca', 'sensibl', 'reserv', 'restrict', 'ascertain', '7091', 'freedom', 'fate', 'plausibl', 'ignor', 'desir', 'domain', 'excit', 'themselv', 'interlac', 'realli', 'microbiologist', 'overarch', 'timesc', 'hitherto', 'simpli', 'instrument', 'contradictori', 'reproduc', 'sudden', 'attrit']
T32_lev2
['fewer', 'tire', 'coerciv', 'enlarg', 'sssrm', 'malefg', 'papermak', 'lhsrm', 'squirrel', 'bpmsm', 'epstein', 'cylindroid', 'aero', 'pullulana', 'deflector', 'bemf', 'vermicular', 'thermosiphon', 'hcps', 'ycwg', 'waffl', 'springback', 'aerostat', 'ymphyta', 'forklift', 'bdfm', 'orthogon', 'stepper', 'rmxprt', 'stub', 'supermini', 'tehd', 'airflux', 'hspmm', 'everett', 'bait', 'synrm', 'psat', 'afpm', 'exoskeleton', 'synrelm', 'dsim', 'rbfn', 'magnetomot', 'kbsa', 'blsrm', 'adalin', 'pmsgs', 'maltooligosaccharid', 'slip']
T33_lev2
['alreadi', 'groyn', 'rever', 'counteract', 'empha', 'advoc', 'gross', 'mark', 'tend', 'earlier', 'american', 'setup', 'sought', 'warn', 'bench', 'databa', 'whenev', 'prerequisit', 'vertic', 'northwestern', 'mega', 'favour', 'inher', 'patchi', 'guid', 'depict', 'moment', 'buri', 'shorten', 'columbia', 'herein', 'endang', 'rich', 'agrarian', 'multipl', 'hand', 'rank', 'regardless', 'away', 'viabl', 'cattl', 'stimul', 'street', 'pump', 'colorado', 'sutcliff', 'overestim', 'boost', 'third', 'almost']
T34_lev2
['exist', 'held', 'nurseri', 'intraspecif', 'star', 'herb', 'harvest', 'youngest', 'background', 'exacerb', 'vomit', 'inconsist', 'brazilian', 'bias', 'german', 'prairi', 'group', 'tundra', 'anoth', 'anisotrop', 'equip', 'unprec', 'indiana', 'royal', 'chine', 'contin', 'format', 'rectifi', 'trough', 'longstand', 'countrysid', 'temporari', 'pore', 'kentucki', 'trichlori', 'himalayan', 'spider', 'mucunoid', 'conclusionw', 'karoo', 'uttum', 'sibirica', 'chenopodium', 'fraxinus', 'mandshurica', 'switchgrass', 'twqr', 'lycopersicum', 'camaldulensi', 'collar']
T35_lev2
['slns', 'diversifi', 'intraven', 'cumul', 'incub', 'schedul', 'electrophoresi', 'class', 'grown', 'steadili', 'hazelnut', 'rectum', 'valid', 'rest', 'predispo', 'mere', 'fulli', 'anali', 'germlin', 'irrespect', 'long', 'twice', 'chemoprev', 'thirti', 'interst', 'nationwid', 'inabl', 'strong', 'constitut', 'macular', 'comfort', 'mucosa', 'correl', 'phorbol', 'lymphoma', 'action', 'micronutri', 'herb', 'grape', 'mapk', 'goal', 'crude', 'compat', 'categor', 'accid', 'pork', 'weak', 'program', 'recur', 'overexpress']
T36_lev2
['mobili', 'involv', 'oregon', 'assess', 'unstabl', 'refin', 'constitut', 'flavan', 'maxim', 'aluminum', 'epicatechin', 'sporad', 'spent', 'tire', 'clog', 'throughout', 'appreci', 'suffici', 'picker', 'hatcheri', 'minimi', 'rather', 'excipi', 'forag', 'sylvestri', 'pipelin', 'sandi', 'cerevisia', 'radon', 'earthen', 'make', 'distribut', 'lipophil', 'extract', 'median', 'sucro', 'avoid', 'debat', 'exfiltr', 'emphasi', 'barium', 'progeni', 'regist', 'gold', 'biospher', 'saal', 'recogn', 'prevail', 'capit', 'near']
T37_lev2
['match', 'gradual', 'miniatur', 'besid', 'superson', 'burst', 'anom', 'least', 'among', 'mention', 'regular', 'anthropomorph', 'laid', 'reflector', 'prevail', 'absolut', 'highlight', 'klinkenberg', 'marangoni', 'oblat', 'rabigh', 'xisha', 'submarin', 'naamp', 'scallop', 'cpdhn', 'densimetr', 'ketofol', 'grashof', 'hematocrit', 'herringbon', 'overpredict', 'cpde', 'runout', 'slipper', 'phpaam', 'standoff', 'casson', 'cdms', 'descend',

'strongest', 'dichotom', 'swallow', 'hamiltonian', 'popular', 'migratori', 'intrigu', 'tuber', 'lock', 'squar ']

Top words of topics obtained with hARTM model on the first hierarchical level for balanced WoS dataset (pre-processing 2).

T1_lev1

['gradual', 'boost', 'readili', 'crosslink', 'thaw', 'regardless', '6628', 'pharmacodynam', 'minim', 'distr ess', 'maxim', 'airway', 'carotid', 'issu', 'squamous', 'themselv', 'teen', 'drosophila', 'adren', 'afflict', ' stockpil', 'fission', 'coconut', 'paramount', 'reapprai', 'schwann', 'govadin', 'lidocain', 'cycla', 'proant hocyanidin', 'glycogen', 'subfield', 'mitogen', 'incrna', 'catabol', 'adenocarcinoma', 'egfp', 'tacrin', 'c alpain', 'glutamina', 'oxpho', 'nefa', 'htra', 'gfap', 'taxifolin', 'vasculatur', 'amyloidosi', 'prpc', 'selegil in', 'xfad']

T2_lev1

['mixtur', 'wooden', 'action', 'chile', 'estuarin', 'anoth', 'physiochem', 'palm', 'notabl', 'southeastern', 'spar', 'unsuit', 'broad', 'intensifi', 'destabil', 'germ', 'interrel', 'exploit', 'overcom', 'golden', 'neverthe less', 'widespread', 'cova', 'tabl', 'kenya', 'notwithstand', 'obstruct', 'introduc', 'predomin', 'unfea', 'e mit', 'younger', 'assert', 'bottom', 'minimi', 'microsc', 'lysimet', 'bioret', 'geochem', 'macroinvertebr', 'astm', 'resprout', 'irwh', 'geospati', 'kept', 'rever', 'closur', 'extend', 'organochlorin', 'advantag']

T3_lev1

['aitl', 'attenu', 'polish', 'potenc', 'sibl', 'drastic', 'bioindic', 'oprm', 'anticanc', 'sire', 'rout', 'pinus', 'ca ndida', 'perturb', 'coalesc', 'exact', 'corrobor', 'reconcil', 'organel', 'microfluid', 'ongo', 'glycoprotein ', 'press', 'aldh', 'lifecycl', 'ubiquit', 'cutan', 'dark', 'speciat', 'actinobacteria', 'neighborhood', 'septem b', 'ldlr', 'repres', 'note', 'homogen', 'exot', 'experienc', 'routin', 'center', 'bacteriolog', 'denatur', 'aust ralian', 'subcellular', 'eber', 'beck', '1996', 'repetit', 'articul', 'fret']

T4_lev1

['gaseous', 'trim', '11085', 'pocket', 'unansw', 'correct', 'coverag', 'aster', 'retain', 'watson', 'triad', 'hi nder', 'inhomogen', 'extrapol', 'brui', 'turbin', 'document', 'portabl', 'subtili', 'absenc', 'factori', 'may b', 'cumul', 'leav', 'relativist', 'suppo', 'manno', 'inexpen', 'occa', 'ammonia', 'preci', 'mammalian', 'b egin', 'homo', 'take', 'link', 'peroxida', 'retreat', 'kilometr', 'impur', 'utmost', 'aetiolog', 'conform', 'ch eaper', 'heterogen', 'portland', 'bidirect', 'amongst', 'blast', 'elabor']

T5_lev1

['exagg', 'pleasant', 'continuum', 'wealth', 'lgbtq', 'full', 'preliminari', 'formul', 'accumul', 'unidentifi' , 'permiss', 'phenomena', 'portray', 'submiss', 'worsen', 'manageri', 'placebo', 'advocaci', 'minus', 'ob vious', 'commiss', 'rocket', 'scrambl', 'prize', 'divert', 'basketb', 'area', 'failur', 'trivial', 'discour', 'her eaft', 'elementari', 'caudal', 'desk', 'empti', 'intersect', 'routin', 'rape', 'supplementari', 'sake', 'dimini sh', 'causat', 'hierarchi', 'unsolv', 'door', 'unnecessari', 'avail', 'prompt', 'ordinarili', 'guard']

T6_lev1

['nocicept', 'comput', 'bleed', 'causal', 'night', 'rule', 'seventi', 'acetabular', 'programm', 'tast', 'merck' , 'quicker', 'fall', 'shop', 'albin', 'prodrom', 'undetect', 'christian', 'eleven', 'notabl', 'pace', 'fluent', 'tr ue', 'neuropatholog', 'hemorrhag', 'satisfactori', 'begin', 'malform', 'mncds', 'schizophrenia', 'acyl', ' diurnal', 'wheat', 'protect', 'stabil', 'immin', 'injur', 'besid', 'classic', 'superfici', 'parkinson', 'pairwi', ' insuffici', 'rever', 'biomechan', 'acuiti', 'pgsn', 'pertussi', 'lcis', 'lozeng']

T7_lev1

['lingual', 'trough', 'blank', 'deduc', 'longer', 'mimic', 'stagnant', 'licen', 'taper', 'nanowir', 'spill', 'spa cer', 'isol', 'stagnat', 'nineteen', 'bottleneck', 'emit', 'fuse', 'evid', 'hydro', 'therebi', 'austria', 'gaug', 'v arieti', 'intersect', '8530', 'random', 'tester', 'realli', 'anchorag', 'raindrop', 'aquif', 'topographi', 'corn er', 'stir', 'slower', 'matrix', 'vicin', 'dredg', 'russia', 'soccer', 'took', 'anolyt', 'brittl', 'orthodont', 'silan ', 'trapezoid', 'subcrit', 'dentur', 'loca']

T8_lev1
['correl', 'pacemak', '11624', 'supplier', 'penetr', 'inhabit', 'baselin', 'prece', 'gossip', 'socialbot', 'eav esdrop', 'tangibl', 'memif', 'snort', 'hazard', 'basketb', 'errat', 'ingress', 'heritag', 'spam', 'master', 'apa rt', 'pound', 'ration', 'chart', 'ceil', 'multifacet', 'immedi', 'spirit', 'homo', 'piec', 'tactic', 'possibilist', ' opportun', 'declar', 'xilinx', 'straightforward', 'readili', 'grasp', 'inexpen', 'volatil', 'threaten', 'speed', 'remov', 'belgium', 'accommod', 'ineffici', 'visibl', 'though', 'essenc']
T9_lev1
['shis', 'instead', 'teen', 'hypoth', 'prompt', 'permit', 'percent', 'alongsid', 'spectrum', 'broaden', 'energ et', 'anxiolyt', 'stereotypi', 'roadmap', 'practi', 'steadili', 'uniform', 'central', 'trust', 'unmet', 'pros', 'ex emplifi', 'arous', 'measur', 'video', 'yard', 'weak', 'durat', 'viewpoint', 'holi', 'googl', 'later', 'telecar', ' bureau', 'southwest', 'overrid', 'sentenc', 'thrust', 'conceiv', 'norwegian', 'bibliometr', 'emdr', 'workf orc', 'possess', 'retain', 'dropout', 'pertin', 'unreason', 'scheff', 'catch']
T10_lev1
['agreement', 'eavesdropp', 'serv', 'impract', 'interconnect', 'photograph', 'wait', 'discontinu', 'withst and', 'pairwi', 'granul', 'recov', 'beam', 'hide', 'centimet', 'harri', 'sent', 'inset', 'throw', 'unbound', 'sol enoid', 'airplan', 'yoke', 'prerequisit', 'immer', 'skew', 'superconduct', 'doubli', 'permit', 'learnt', 'insi d', 'lithium', 'truck', 'tracker', 'cyclic', 'conquer', 'highest', 'cardin', 'figur', 'euler', 'electromechan', 's trang', 'finer', 'microscop', 'circuitri', 'gibb', 'intra', 'bright', 'hitherto', 'spurious']

Top words of topics obtained with hARTM model on the second hierarchical level for balanced WoS dataset (pre-processing 2).

T1_lev2
['polar', 'gastrointestin', 'unaccept', 'cough', 'distinguish', 'base', 'remark', 'dival', 'qualit', 'pack', 'clo se', 'receiv', 'recipi', 'illumin', 'enterocyt', 'ternari', 'retinoid', 'niger', 'entranc', 'regard', 'foren', 'quin on', 'facilit', 'degrad', 'pilot', 'fresh', 'exemplifi', 'thousand', 'smaller', 'perhap', 'make', 'avoid', 'mad e', 'window', 'parallel', 'plastic', 'scan', 'prefer', 'throughout', 'deltamethrin', 'bare', 'subtract', 'immu nostimul', 'hous', 'discov', 'purif', 'certain', 'dinucleotid', 'intragastr', '8491']
T2_lev2
['reconnaiss', 'estuarin', 'appear', 'urgent', 'introduc', 'stagnant', 'offici', 'autom', 'dipol', 'florida', 'be rlin', 'model', 'presum', 'abund', 'hangzhou', 'interspeci', 'discontinu', 'digiti', 'graze', 'alberta', 'some tim', 'floor', 'monoton', 'ortho', 'impli', 'colder', 'remain', 'tongu', 'intercept', 'evidenti', 'link', 'aeron aut', 'seab', 'suit', 'lookup', 'late', 'profit', 'uniform', 'heritag', 'broader', 'multisourc', 'individu', 'com prehen', 'uninterrupt', 'baselin', 'artifact', 'notic', 'featur', 'conduct', 'territori']
T3_lev2
['email', 'delhi', 'romanian', 'elig', 'clear', 'preserv', 'gradual', 'abus', 'alongsid', 'explanatori', 'fashio n', 'strict', 'exemplifi', 'imparti', 'itali', 'sweden', 'transform', 'uncommon', 'inten', 'intend', 'convinc', 'divi', 'verbatim', 'exacerb', 'legisl', 'strive', 'deter', 'popular', 'grew', 'format', 'marketplac', 'biograp h', 'manner', 'ideal', 'avenu', 'fifti', 'book', 'begin', 'congress', 'surround', 'remark', 'shelter', 'cohe', 'r epli', 'centr', 'stratif', 'dissimilar', 'disput', 'occurr', 'sent']
T4_lev2
['accur', 'lost', 'evolv', 'perturb', 'complaint', 'patch', 'brown', 'taken', 'strongest', 'appendag', 'vital', ' zealand', 'nevertheless', 'strengthen', 'origin', 'safe', 'tortuo', 'take', 'coordin', 'suspicion', 'asymmetr' , 'quantif', 'allograft', 'offici', 'themselv', 'corrobor', 'proof', 'brazilian', 'fluent', 'analyz', 'percent', 'a nali', 'immunosuppress', 'expedit', '9810', 'light', 'emphysema', 'nfts', 'indistinguish', 'england', 'do main', 'undertaken', 'labour', 'mainland', 'unresolv', 'insight', 'mood', 'pittsburgh', '8479', 'undesir']
T5_lev2
['advent', 'render', 'least', 'visibl', 'offlin', 'penetr', 'multitask', 'batch', 'bayesian', 'press', 'snapshot', ' tablet', 'homogen', 'practic', 'stop', 'coupl', 'quicker', 'reject', 'offen', 'resolv', 'structur', 'design', 'ma

ndatori', 'distinct', 'probabilist', 'contrari', 'reflector', 'multius', 'sketch', 'allevi', 'sound', 'made', 'malfunct', 'inher', 'shield', 'proof', 'prior', 'periph', 'exit', 'rethink', 'harden', 'inva', 'garbag', 'artifact', 'pseudo', 'chart', 'awaken', 'drastic', 'disjoint', 'nonetheless']

T6_lev2

['movi', 'discoveri', 'disadvantag', 'remedi', 'stand', 'microblog', 'basca', 'hevc', 'tcam', 'hopcncc', 'gepsvm', 'libflasm', 'post', 'ksrc', 'lstm', 'daubechi', 'semisupervi', 'jpeg', 'grayscal', 'throughput', 'weber', 'retrain', 'stepwi', 'prohibit', 'whiten', 'king', 'craft', 'otherwi', 'ceil', 'celebr', 'alon', 'return', 'unbalanc', 'promin', 'fragil', 'abnorm', 'larger', 'conquer', 'erron', 'carv', 'spatiotempor', 'multitempor', 'reach', 'possibl', 'hough', 'appropri', 'lasso', 'wang', 'road', 'exud']

T7_lev2

['bottom', 'feder', 'inexpen', 'crown', 'scope', 'detent', 'cook', 'newli', 'pollut', 'hilli', 'exclus', 'specifi', 'decid', 'predomin', 'spanish', 'globe', 'biggest', 'tight', 'accomplish', 'attent', 'amplifi', 'bound', 'desir', 'robust', 'amongst', 'octob', 'ambigu', 'shorter', 'dure', 'secondari', 'street', 'incent', 'inflow', 'citrus', 'heavili', 'easili', 'reliev', 'demograph', 'procedur', 'unexpect', 'strict', 'attract', 'ration', 'concept', 'unlik', 'agro', 'light', 'tabul', 'worsen', 'intend']

T8_lev2

['insight', 'intol', 'civic', 'autonomi', 'diminish', 'discomfort', 'requir', 'placement', 'pakistan', 'regul', 'kasr', 'eject', 'escap', 'fetal', 'varieti', 'egocentr', 'symptomatolog', 'spain', 'epilepsi', 'upper', 'expiratori', 'either', 'rural', 'match', 'detoxif', '4081', 'febril', 'prematur', 'warmth', 'systemat', 'went', 'unlik', 'holist', 'likert', 'crystal', 'yale', 'modifi', 'subclin', 'pair', 'volunt', 'plural', 'brazilian', 'borderlin', 'activ', 'perpetr', 'medit', 'repress', 'bedroom', 'diagnosi', 'increment']

T9_lev2

['satisfi', 'grip', 'corner', 'quick', 'lymphoma', 'nerv', 'predispo', 'ltda', 'editora', 'counsel', 'longterm', 'stoke', 'commenc', 'depth', 'wider', 'pedro', 'foetal', 'within', 'laparoscop', 'lewi', 'unusu', 'distinct', 'sost', 'harri', 'request', 'traver', 'encompass', 'virolog', 'test', 'irrespect', 'cranial', 'around', 'urinari', 'recov', 'coincid', 'mucosa', 'fifth', 'antiretrovir', 'reclassifi', 'referenc', 'curat', 'intol', 'pilot', 'neoplasia', 'pharmacotherapi', 'moreov', 'immobil', 'femor', 'medullari', 'squar']

T10_lev2

['utero', 'spatiotempor', 'colli', 'hectar', 'yangtz', 'drawn', 'tunisia', 'southwestern', 'although', 'check', 'juvenil', 'succeed', 'light', 'toxic', 'java', 'lesson', 'without', 'fibr', 'encroach', 'opportun', 'permit', 'abroad', 'attempt', 'think', 'crude', 'grade', 'leakag', 'wide', 'absenc', 'seismic', 'manur', 'referenc', 'archaeolog', 'extra', 'plausibl', 'logit', 'madrid', 'vast', 'unsaf', 'tabl', 'microhabitat', 'equilibrium', 'antimicrobi', 'therein', 'harsh', 'rose', 'inadequ', 'unsuit', 'flank', 'illicit']

T11_lev2

['penal', 'titl', 'noteworthi', 'simpli', 'pencil', 'uncoupl', 'effort', 'apart', 'predefin', 'routin', 'emphasi', 'lambda', 'hard', 'varieti', 'radial', 'especi', 'deduc', 'trajectori', 'complianc', 'integro', 'tomo', 'lexicograph', 'aosvr', 'inviscid', 'steinmetz', 'monomi', 'stencil', 'diffeomorph', 'belfast', 'alya', 'traceless', 'tmdl', 'chapman', 'petiga', 'isoc', 'cocycl', 'conclud', 'reveal', 'build', 'strive', 'queen', 'fluenc', 'inspir', 'photogrammetr', 'freedom', 'descent', 'straightforward', 'eulerian', 'sinc', 'similar']

T12_lev2

['third', 'explicit', 'slower', 'refin', 'email', 'mental', 'assumpt', 'multifacet', 'flash', 'sequenti', 'analog', 'coher', 'collat', 'disinhibit', 'lead', 'held', 'adaptor', 'tribal', 'theorist', 'shot', 'nonsignif', 'adduct', 'gentl', 'shave', 'whose', 'influenti', 'associ', 'affirm', 'extra', 'english', 'permit', 'height', 'shed', 'select', 'fake', 'multilingu', 'stop', 'wisdom', 'unclear', 'abov', 'refer', 'elucid', 'neither', 'hormon', 'russel', 'shape', 'intern', 'axillari', 'sincer', 'anova']

T13_lev2

['report', 'built', 'exhibit', 'connect', 'rest', 'viabl', 'criterion', 'digest', 'univari', 'bill', 'gmbh', 'asymmetri', 'payload', 'refer', 'wrong', 'arbitrarili', 'pack', 'supplementari', 'chameleon', 'central', 'runtim', 'paid', 'wire', 'permiss', 'subgroup', 'massiv', 'speed', 'merchant', 'detect', 'membership', 'basic', 'pate

nt', 'seek', 'resembl', 'preval', 'spread', 'theori', 'straightforward', 'altern', '1926', 'latter', 'batch', 'fragment', 'peculiar', 'crosstalk', 'ultra', 'commit', 'harder', 'http', 'commut']

T14_lev2

['anom', 'charger', 'evacu', 'departur', 'simplifi', 'split', 'weak', 'pellet', 'hand', 'match', 'port', 'irrev', 'liter', 'miss', 'toroid', 'suggest', 'gateway', 'late', 'wherebi', 'park', 'invent', 'switzerland', 'unaffect', 'intertwin', 'recess', 'duct', 'empha', 'creativecommon', 'poison', 'readili', '7310', 'inward', 'otherwi', 'orient', 'prefer', 'meant', 'cermet', 'heav', 'occurr', 'shave', 'longer', 'sunlight', 'provi', 'speciat', 'stiffen', 'decoupl', 'tall', 'carlo', 'inabl', 'done']

T15_lev2

['australian', 'king', 'tend', 'influenc', 'twelv', 'compen', 'gearbox', 'free', 'colleagu', 'subsequ', 'room', 'massachusett', 'languag', 'hypoth', 'coast', 'wheel', 'much', 'ethnographi', 'obtain', 'drastic', 'tech', 'ever', '5082', 'plug', 'read', 'ball', 'modular', 'fellow', 'submit', 'push', 'georgia', 'accompani', 'younger', 'impart', 'listen', 'symbol', 'outsid', '2782', 'western', 'contrast', 'math', 'blue', 'greatest', 'necessit', 'auto', 'undergo', 'biograph', 'accid', '4664', 'commiss']

T16_lev2

['current', 'ioniz', 'exceed', 'divid', 'increa', 'regular', 'mismatch', 'proof', 'antimicrobi', 'condit', 'singapor', 'west', 'quick', 'detoxif', 'delimit', 'immedi', 'attenu', 'embryon', 'inocul', 'contemporari', 'suffici', 'line', '9135', 'catalogu', 'simultan', 'cytogenet', 'retain', 'trace', 'gather', 'unequivoc', 'peel', 'draft', 'later', 'substrat', 'homozygot', 'birth', 'http', 'modif', 'stripe', 'gata', 'eater', 'therebi', 'brazil', 'mechanist', 'transpo', 'iran', 'barrier', 'trim', 'alien', 'linnaeus']

T17_lev2

['manifest', 'preliminari', 'alway', 'sentenc', 'request', 'symmetri', 'recollect', 'attain', 'extrapol', 'inflat', 'suppress', 'facebook', 'total', 'stretch', 'momentari', 'underpin', 'debilit', 'expan', 'oneself', 'concret', 'conver', 'constraint', 'exclud', 'color', 'german', 'regular', 'consensu', 'stori', 'full', 'harder', 'powerless', 'affluent', 'warmer', 'recogn', 'yield', 'hundr', 'unattract', 'note', 'virtuous', 'relianc', 'elimin', 'letter', 'oppon', 'amelior', 'owner', 'contagi', 'pivot', 'bayesian', 'wherebi', 'virtual']

T18_lev2

['govern', 'zinc', 'unwant', 'accord', 'afflict', 'migratori', 'hematolog', 'sclerosi', 'label', 'rodent', 'intrana', 'under', 'antagon', 'reli', 'vasculatur', 'prognosi', 'shorter', 'vein', 'retent', 'ultrastructur', 'begin', 'mesent', 'ioniz', 'transduc', 'becam', 'lumin', 'lipolysi', 'length', 'unalt', 'immunoblot', 'would', 'huvec', 'fgcs', 'brdu', 'simvastatin', 'tslp', 'dpsc', 'trpm', 'gingipain', 'oculoplast', 'fifibroblast', 'abdsc', 'planarian', 'breve', 'importin', 'fimbria', 'alkalosi', 'lsds', 'tnbs', 'proapoptot']

T19_lev2

['said', 'verbatim', 'unsatisfactori', 'triall', 'succeed', 'ambiti', 'without', 'today', 'quick', 'merchant', 'enjoy', 'twenti', 'redund', 'technolog', 'perform', 'unexplor', 'invit', 'voluntari', 'complement', 'interdisciplinari', 'counter', 'script', 'warrant', 'analyt', 'spoke', 'former', 'netherland', 'much', 'economist', 'heterogen', 'difficulti', 'endeavour', 'toward', 'feedback', 'monoton', 'duti', 'elicit', 'necessarili', 'client', 'compliant', 'produc', 'question', 'revenu', 'frustrat', 'moreov', 'step', 'consist', 'correct', 'accomplish', 'builder']

T20_lev2

['pursuit', 'grade', 'actual', 'elus', 'pitfal', 'capac', 'simpl', 'surpri', 'major', 'tell', 'flourish', 'discrep', 'bolster', 'restrain', 'smooth', 'inabl', '8912', 'impoverish', 'multifacet', 'teen', 'evolutionari', 'engend', 'unsuccess', 'except', 'behind', 'exogen', 'manageri', 'elabor', 'vantag', 'debat', 'broaden', 'gradual', 'characteri', 'intercour', 'possess', 'pride', 'conceptuali', 'fail', 'tradeoff', 'basi', 'fifti', 'adjust', 'preserv', 'negat', 'earlier', 'masteri', 'neuroendocrin', 'seat', 'naturalist', 'revi']

T21_lev2

['restrict', 'exampl', 'extern', 'constitu', 'prefabr', 'utili', 'analysi', 'build', 'surpri', 'interf', 'know', 'tight', 'envelop', 'clean', 'seab', 'downward', 'frequent', 'comprehen', 'increment', 'favor', 'given', 'tetrahedr', 'stringent', 'absenc', 'empha', '6067', 'understand', 'exist', 'inflex', 'undertaken', 'devic', 'progr

am', 'insert', 'intact', 'databa', 'gain', 'joint', 'consequ', 'reconstitut', 'phenomenon', 'areal', 'execut', 'bureau', 'escap', 'peripheri', 'highlight', 'breakag', 'hydrostat', 'municip', 'suscept']

T22_lev2

['depend', 'descript', 'regain', 'fidel', 'weakest', 'membran', 'isol', 'contralat', 'accompani', 'manipul', 'consum', 'trauma', 'formic', 'radiographi', 'write', 'differenti', 'ballist', 'static', 'chondrocyt', 'bilay', 'dosag', 'thorac', 'moor', 'flaw', 'cation', 'least', 'receiv', 'augment', 'diffus', 'flexur', 'skeleton', 'sonograph', 'somewhat', 'dedic', 'granul', 'inert', 'locat', 'bench', 'amen', 'degrad', 'unlik', 'histolog', 'sever', 'justifi', 'trabecular', 'labor', 'entir', 'medium', 'zeta', 'plaqu']

T23_lev2

['detent', 'newli', 'almost', 'beamform', 'seek', 'succ', 'rfid', 'changeabl', 'inclus', 'move', 'promin', 'island', 'oscillatori', 'rever', 'drastic', 'proof', 'accomplish', 'mppt', 'bode', 'emitt', 'verilog', 'modular', 'submicron', 'cnns', 'within', 'lyapunov', 'still', 'shorten', 'despit', 'sampler', 'waveguid', 'multimod', 'clic', 'illustr', 'cutoff', 'origin', 'multilay', 'versus', 'polymet', 'lossless', 'shis', 'deterior', 'kalman', 'clear', 'drawback', 'etait', 'tackl', 'storey', 'rise', 'gradient']

T24_lev2

['close', 'campaign', 'characteri', 'prospect', 'optimum', 'durat', 'none', 'uncorrel', 'promin', 'narrow', 'autocorrel', 'consequ', 'second', 'coordin', 'version', 'third', 'mixtur', 'consum', 'stick', 'pertain', 'popular', 'shot', 'buoyant', 'pairwi', 'inten', 'routin', 'presenc', 'compil', 'occurr', 'principl', 'neglig', 'distribut', 'bodi', 'reserv', 'requir', 'compromi', 'cutoff', 'percent', 'inaccuraci', 'either', 'endolymph', 'csnn', 'reopen', 'venturi', 'usual', 'area', 'conveni', 'straightforward', 'balanc', 'probabilist']

T25_lev2

['legaci', 'emit', 'wait', 'success', 'princip', 'changeabl', 'inher', 'trend', 'artifici', 'downtim', 'interchang', 'still', 'mention', 'impo', 'depot', 'investig', 'favor', 'breakag', 'cheap', 'foundat', 'railway', 'crucial', 'upon', 'recogn', 'commiss', 'expedit', 'multilay', 'section', 'chain', 'unfea', 'compli', 'three', 'sort', 'promi', 'unifi', 'studi', 'known', 'subcompon', 'disadvantag', 'kaizen', 'figur', 'extrus', 'almost', 'intermedi', 'finish', 'formal', 'modem', 'graphic', 'scratch', 'drank']

T26_lev2

['experi', 'estradiol', 'tracer', 'caus', 'intracerebr', 'neurotox', 'histon', 'anesthesia', 'told', 'counterpart', 'spent', 'cyclic', 'onto', 'adra', 'brainstem', 'subset', 'unfair', 'threshold', 'address', 'disinhibit', '5579', 'feedback', 'cortisol', 'predictor', 'hidden', 'neuropath', 'minut', 'hydrochlorid', 'strike', 'simultan', 'prosodi', 'psychoact', 'counteract', 'lack', 'cage', 'lobular', 'disconnect', 'cent', 'inflex', 'scanner', 'confirm', 'sole', 'compel', 'asymmetr', 'glia', 'ration', 'gradual', 'breath', 'bradykinin', 'orexin']

T27_lev2

['thereof', 'https', 'extraordinari', 'hinder', 'sacrif', 'favor', 'immunoprecipit', 'page', 'cytolog', 'catalog', 'lifecycl', 'shape', 'clone', 'adeno', 'milieu', 'retino', 'afford', 'faith', 'characteri', 'fecal', 'oblig', 'tolerogen', 'chyma', 'scale', 'outlin', 'erythrocyt', 'spragu', 'inva', 'comprehen', 'intim', 'phosphata', 'termin', 'occupi', 'warsaw', 'give', 'union', 'less', 'accel', 'allevi', 'streamlin', 'lactobacillus', 'mutagenesi', 'hela', 'breakdown', 'creat', 'calv', 'showca', 'prolin', 'contracept', 'epstein']

T28_lev2

['termin', 'poor', 'barcelona', 'enrich', 'retinoid', 'broader', 'frozen', 'strong', 'formula', 'repetit', 'respond', 'aeruginosa', 'enhanc', 'consumpt', 'imbal', 'clinicopatholog', 'taiwan', 'fourth', 'hypercholesterolemia', 'histolog', 'reduct', 'bovin', 'occa', 'radon', 'type', 'intraperiton', 'subcutan', 'characterist', 'length', 'polyarticular', 'matur', 'phylum', 'expenditur', 'nest', 'sinc', 'serv', 'preeclampsia', 'quantil', 'noninva', 'particular', 'data', 'peak', 'stay', 'typic', 'repr', '8764', 'vapour', 'immunohistochemistri', 'intrigu', 'order']

T29_lev2

['cloth', 'reloc', 'near', 'overnight', 'exclud', 'ongo', 'coff', 'succeed', 'suffer', 'primari', 'search', 'chosen', 'street', 'alik', 'disconnect', 'reproduc', 'divert', 'naturalist', 'mode', 'frail', 'crucial', 'eccentr', 'hinder', 'biom', 'avoid', 'constrain', 'empha', 'click', 'leverag', 'slave', 'viewpoint', 'alert', 'seven', 'fuse', '

coin', '4294', 'impreci', 'articul', 'shed', 'personali', 'necessit', 'sedentari', 'notion', 'multipath', 'sopc', 'proprietari', 'unwant', 'consist', 'newer', 'omnidirect']

T30_lev2

['fall', 'never', 'disconnect', 'obscur', 'incept', 'primari', 'path', 'shape', 'interrel', 'deduc', 'reader', 'handl', 'inquiri', 'receiv', 'corrobor', 'blow', 'divi', 'ensu', 'mark', 'true', 'incr', 'push', 'dyer', '10009', 'book', 'philosophi', 'endeavour', 'analysi', 'upcom', 'confer', 'reali', 'accept', 'conjunct', 'respon', 'prioriti', 'tabl', 'journey', 'resurg', 'longest', 'adapt', 'strong', 'restrict', 'instanc', 'multitud', 'showca', 'adopt', 'necessit', 'judgeabl', 'mysteri', 'deep']

T32_lev2

['unlik', 'asynchron', 'practic', 'coerciv', 'epstein', 'aurora', 'pico', 'solidwork', 'usag', 'workspac', 'counterfeit', 'xmgfe', 'tire', 'smooth', 'contribut', 'discrep', 'launch', 'greater', 'resolv', 'fulfil', 'slip', 'relev', 'seek', 'groov', 'lift', 'shift', 'accel', 'satisfactorili', 'attain', 'strategi', 'truck', 'function', 'step', 'fourier', 'intensifi', 'onlin', 'novelti', 'feder', 'kept', 'fold', 'promin', 'connect', 'sometim', 'enlarg', 'simplif', 'refrig', 'prioriti', 'undertak', 'meanwhil', 'command']

T33_lev2

['span', 'snow', 'reli', 'adjust', 'vine', 'bathymetri', 'aquacultur', 'deterior', 'hyper', 'alloc', 'convent', 'databa', 'nevertheless', 'manur', 'residu', 'paraguay', 'minus', 'photograph', 'nile', 'eight', 'manual', 'centuri', 'uniform', 'java', 'subtl', 'overestim', 'entri', 'wetter', 'note', 'satisfactorili', 'spot', 'footprint', 'mainten', 'current', 'upward', 'expan', 'insignif', 'transit', 'shoal', 'compact', 'hurrican', 'waterlog', 'attempt', 'front', 'multicriteria', 'penetr', 'intend', 'holder', 'class', 'franc']

T34_lev2

['guid', 'likewi', 'nuclei', 'track', 'inconsist', 'appropri', 'recip', 'counter', 'sought', 'exogen', 'guard', 'malondialdehyd', 'center', 'genera', 'quick', 'newli', 'goal', 'intact', 'grave', 'prerequisit', 'unabl', 'inher', 'span', 'carbohydr', 'taller', 'appar', 'agronom', 'alli', 'outweigh', 'provok', 'herbicid', 'massiv', 'empha', 'shorter', 'alik', 'dealt', 'intra', 'workshop', 'enough', 'micronutri', 'alien', 'photochem', 'scrub', 'cholestasi', 'seem', 'monocultur', 'visibl', 'russian', 'nigra', 'collar']

T35_lev2

['intraocular', 'reach', 'relax', 'straightforward', 'around', 'incompat', 'chief', 'behalf', 'iran', 'fact', 'expedit', 'epiderm', 'paradox', 'hypoth', 'appli', 'protect', 'gave', 'controversi', 'lifetim', 'mandatori', 'took', 'asept', 'done', 'steadili', 'uniform', 'driver', 'small', 'postmenopaus', 'germlin', 'vulgari', 'larger', 'impart', 'adeno', 'past', 'reconsid', 'irish', 'inabl', 'mammalian', 'undesir', 'eleven', 'phenomenon', 'immunotherapeut', 'itali', 'potassium', 'mapk', 'antagonist', 'throat', 'began', 'specialti', 'dephosphoryl']

T36_lev2

['behind', 'choo', 'zero', 'woodland', 'humid', 'actual', 'golden', 'backbon', 'compli', 'begin', 'impact', 'option', 'cyprinid', 'pore', 'respond', 'start', 'close', 'regrowth', 'hold', 'flat', 'deduc', 'superior', 'ultra', 'dead', 'eleven', 'clog', 'common', 'literatur', 'lithium', 'beverag', 'eman', 'aurora', 'buildup', 'stratum', 'biogeochem', 'bioluminesc', 'disturb', 'grow', 'react', 'zeta', 'explain', 'meal', 'nylon', 'abandon', 'colon', 'keep', 'holist', 'februari', 'sichuan', 'facilit']

T37_lev2

['swallow', 'promin', 'hematocrit', 'favour', 'besid', 'guidelin', 'appropri', 'cushion', 'camera', 'seawat', 'topolog', 'dramat', 'blend', 'postul', 'descend', 'trend', 'exampl', '4424', 'idea', 'strongest', 'expo', 'meander', 'argon', 'adiabat', 'coaptat', 'plung', 'laryng', 'kick', 'spring', 'unaffect', 'trail', 'tendenc', 'current', 'otherwi', 'incorpor', 'trigger', 'interdepend', 'sheet', 'beyond', 'fine', 'motil', 'stratifi', 'fourier', 'laid', 'highlight', 'compressor', 'steep', 'irregular', 'electrostat', 'help']

Top words of topics obtained with hPAM model on the second hierarchical level for balanced WoS dataset (pre-processing 1), $\eta = 0.5$.

T1
['written', 'feminin', 'extract', 'veteran', 'appar', 'valuabl', 'write', 'almost', 'near', 'suffici', 'partnership', 'humor', 'unrel', 'checklist', 'inappropri', 'respiratori', 'hour', 'germani', 'absent', 'clarifi', 'summar', 'atyp', 'uncertainti', 'predomin', 'intuit', 'acut', 'site', 'scholar', 'forward', 'broader', 'guilt', 'mainten', 'quit', 'drawn', 'contrari', 'synchron', 'agreeabl', 'bystand', 'ambiv', 'vital', 'matur', 'occurr', 'interdisciplinari', 'nevertheless', 'argument', 'dyadic', 'intend', 'felt', 'upnt', 'caruso']

T2
['amplitud', 'synergist', 'cgmcc', 'appetit', 'fusarium', 'messeng', 'cancer', 'goat', 'much', 'actin', 'dis muta', 'monom', 'acquisit', 'carbon', 'pyrrolidin', 'polypeptid', 'microbiolog', 'quantit', 'strong', 'aum an', 'alkalin', 'green', 'orientali', 'gdps', 'taker', 'hypoth', 'electrostat', 'micronutri', nan, 'etoac', 'deliv ', 'pancrea', 'batch', 'kill', 'lignin', 'immunofluoresc', 'spine', 'hydrochlorid', 'rhces', 'estradiol', 'trace r', 'palat', 'ketamin', 'chamber', 'leav', 'cleft', 'scopolamin', 'transfect', 'mocv', 'amph']

T3
['laidlawii', 'prevel', 'negro', 'heartburn', 'spore', 'silico', 'mainland', 'bleed', 'subaxi', 'apai', 'zooplan kton', 'diagno', 'cytocompat', 'benign', 'thick', 'fluvial', 'latissimus', 'sweden', 'fucus', 'aerobiolog', 's tay', 'haplogroup', 'aortic', 'cerclag', 'replica', 'avoid', 'physico', 'objectif', 'vitro', 'achiev', 'ardiscove ri', 'grandi', 'physiochem', 'cardiopulmonari', 'sometim', 'personn', 'read', 'bangladesh', 'humanist', ' masset', 'interchang', 'corro', 'clifford', 'immunotherapi', 'homm', 'downrush', 'dentsplysirona', 'gou t', 'ainsi', 'craftolog']

T4
['lethal', 'deliv', 'ectop', 'kill', 'million', 'phage', 'ablat', 'plus', 'sperm', 'immunofluoresc', 'reproduc', 'bear', 'tendon', 'helper', 'intron', 'communiti', 'distanc', 'note', 'fibrosi', 'tend', 'wall', 'epiderm', 'alm ost', 'leav', 'assign', 'perturb', 'visit', 'offspr', 'give', 'exosom', 'osteoclast', 'sacroili', 'geograph', 'met abolom', 'under', 'threshold', 'methodolog', 'kyphosi', 'fatal', 'prescrib', 'chaperon', 'homozyg', 'croh n', 'librari', 'erythematosus', 'histon', 'biochemistri', 'neuropatholog', 'upper', 'cost']

T5
['stabili', 'kinet', 'embryogenesi', 'retain', 'homogen', 'phosphat', 'pathologist', 'edat', 'ablat', 'vein', 't fts', 'plasmacyt', 'cellobio', 'elegan', 'atresia', 'ssris', 'psnr', 'cotton', 'tobago', 'unexplain', 'would', 'dr ess', 'perpendicular', 'optogenet', 'nonfat', 'inflow', 'decid', 'collat', 'nanofib', 'extub', 'mesenchym', ' algin', 'nobori', 'fibrin', 'lymphoendothelium', 'reuptak', 'occas', 'cretac', 'oleagin', 'plastin', 'import', 'gondwana', 'form', 'know', 'reattach', 'semiperm', 'hilum', 'autolog', 'wavefield', 'emboli']

T6
['aeuro', 'volcano', 'bitstr', 'essay', 'pdam', 'ivoclar', 'anthropometri', 'condominium', 'fluidi', 'carabi da', 'crosswi', 'predecessor', 'dodi', 'lengthwi', 'ethnomedicin', 'mungo', 'hellenist', 'inlab', 'xukou', ' arbitrarili', 'lncs', 'cicer', 'germain', 'impair', 'hogno', 'climat', 'canal', 'tape', 'cloudsat', 'tyre', 'ellig', ' bonferroni', 'circular', 'artifactu', 'coron', 'seedless', 'rediscoveri', 'luigi', 'school', 'dlsw', 'bathtub', 't ibet', 'massachusett', 'salewa', 'flake', 'rossler', 'tanneri', 'bore', 'customiz', 'sass']

T7
['order', 'freundlich', 'ccsr', 'homogen', 'proxim', 'icef', 'foil', 'heterocycl', 'minim', 'readili', 'obvious ', 'rhfix', 'maldi', 'hous', 'monolith', 'dosimetri', 'phenylimidazo', 'fluidiz', 'sphingolipid', 'flora', 'all oy', 'sulphid', 'gumenek', 'caroten', 'nicotin', 'biogeochem', 'benzophenon', 'orchard', 'cymen', 'ghgs ', 'arachidon', 'ofbr', 'seen', 'except', 'pbcho', 'nadph', 'pboh', 'flower', 'linker', 'consortium', 'firmicu t', 'undop', 'scaveng', 'granulocyt', 'especi', 'crucial', 'phyla', 'alprazolam', 'relea', 'exceed']

Top words of topics obtained with hPAM model on the second hierarchical level for balanced WoS dataset (pre-processing 2), $\eta = 0.5$.

T1
['citi', 'ownership', 'divid', 'vital', 'insula', 'discount', 'fluenci', 'mainten', 'fiction', 'subordin', 'multiv ari', 'extrav', 'ignor', 'isol', 'ecolog', 'alexithymia', 'confound', 'repetit', 'bulli', 'experienti', 'decod', ' diagno', 'occup', 'amplitud', 'vignett', 'german', 'attain', 'compen', 'suffici', 'evolut', 'pronounc', 'spe cifi', 'offic', 'televi', 'psychot', 'invest', 'revi', 'hair', 'under', 'thirti', 'persua', 'invari', 'databa', 'evok', ' wealth', 'forward', 'overt', 'inclin', 'growth', 'uncertainti']
T2
['excess', 'maxillofaci', 'bite', 'volunt', 'percutan', 'print', 'forc', 'infanc', 'window', 'true', 'wrist', 'illic it', 'melanoma', 'difficulti', 'numer', 'infest', 'adapt', 'commentari', 'coexist', 'target', 'feno', 'predispo ', 'prematur', 'bleed', 'throughout', 'member', 'erap', 'consequ', 'kappa', 'descript', 'fibromyalgia', 'im munocap', 'forward', 'vomit', 'pertussi', 'uptak', 'antimicrobi', 'infertil', 'virus', 'chemotherapi', 'dese nsit', 'pelvic', 'edema', 'morn', 'complementari', 'north', 'toxic', 'stiff', 'flap', 'pgsn']
T3
['thermoelectr', 'carboxyl', 'semiconductor', 'moon', 'chinensi', 'abnorm', 'combat', 'instal', 'assembl ag', 'reform', 'sigma', 'gradual', 'rhizosph', 'thermo', 'coeffici', 'nigeria', 'capacit', 'macau', 'whirl', 'v apour', 'charcoal', 'attain', 'bioavail', 'inert', 'kaolinit', 'sodi', 'gscw', 'characteri', 'gase', 'extratrop', ' holist', 'septemb', 'spontan', 'disk', 'southeast', 'distinguish', 'geotherm', 'endem', 'cold', 'necessari', ' poland', 'nyquist', 'underground', 'bean', 'littl', 'respond', 'fluctuat', 'distanc', 'union', 'briggsa']
T4
['undrain', 'amazon', 'nonlinear', 'wash', 'pave', 'drawdown', 'coir', 'frame', 'relief', 'rfpfm', 'softwar', 'resili', 'inerti', 'accid', 'petiol', 'quantiti', 'nevertheless', 'shield', 'polyest', 'accept', 'broad', 'impo', 'a eolian', 'dredg', 'coordin', 'phantom', 'spatio', 'shore', 'saudi', 'macro', 'timesc', 'systemat', 'roller', 'pl ug', 'miner', 'microchannel', 'tooth', 'ansi', 'woodchip', 'suppress', 'cochlea', 'deficit', 'split', 'regular' , 'adequ', 'torsion', 'hour', 'principl', 'bore', 'inertia']
T5
['mddl', '3056;renin', '6839;paper', '4989;paper', '3860;paper', 'ethnograph', '2700;studi', 'transcran i', '7291;studi', '1695;posit', '3520;induc', 'commentari', '8867;paper', '1826;emerg', '2473;drill', '7 124;ester', '5276;kinet', '8599;trace', '3921;paper', '4214;paper', '7029;paper', '1067;activ', 'disinhi bit', '5363;three', '6768;singl', '11774;main', 'anaphylaxi', '8113;three', '1302;fiber', '9432;small', ' 8356;novel', '9846;rapid', 'decontamin', '2246;studi', '1591;water', 'officinali', '4160;paper', '6571; evalu', '9836;studi', '4127;digit', '5018;digit', '7514;purpo', 'deindividu', '528;review', '8291;sever', '8346;evalu', '10960;emot', 'catastroph', 'fingolimod', '10153;fuel']
T6
['dron', 'nnos', 'recognit', 'edaravon', 'insid', 'diminish', 'glycidamid', 'address', 'creatin', 'hypometa bol', 'hydroxybutyr', 'extra', 'compul', 'statement', 'flight', 'amyloidosi', 'bird', 'radiat', 'herb', 'tdga', 'return', 'poison', 'fungicid', 'paper', 'concomit', 'spectroscop', 'take', 'glucuronida', 'larva', 'underw', 'lactic', 'issu', 'stronger', 'detoxif', 'autopsi', 'peroxida', 'spectrum', 'extend', 'forc', 'cerebellum', 'aun p', 'proanthocyanidin', 'taker', 'allergen', 'neurobiolog', 'zinc', 'plum', 'peroxisom', 'equiv', 'becom']
T7
['avenu', 'adenosin', 'excel', 'polio', 'henc', 'adam', 'expect', 'prospect', 'rout', 'believ', 'neoplasm', 'bi ologist', 'begun', 'suffici', 'stabl', 'myocard', 'ontolog', 'occurr', 'truncat', 'mobil', 'center', 'hematopo iesi', 'incomplet', 'diminish', 'tkis', 'cycla', 'layer', 'prepar', 'vast', 'clarifi', 'citrus', 'pbmc', 'belong', ' diversif', 'deleteri', 'sporad', 'codon', 'erbb', 'benefici', 'agap', 'htra', 'trade', 'avoid', 'drosophila', 'co mparison', 'purpo', 'incrna', 'massiv', 'acquisit', 'elegan']

Top words of topics obtained with hPAM model on the second hierarchical level for 20 Newsgroups dataset (pre-processing 1), $\eta = 0.2$.

T1
['select', 'ceas', 'pervers', 'status', 'saturn', 'style', 'depth', 'primarili', 'aris', 'osrh', 'furthermor', 'berna dett', 'risk', 'advic', 'supernatur', 'mistaken', 'thoma', 'queen', 'foolish', 'throw', 'usenet', 'heal', 'shalt'

, 'okcforum', 'proud', 'counter', 'luther', 'nonsens', 'solid', 'kulikauska', 'stalin', 'crime', 'welcom', 'brent', 'below', 'touch', 'speech', 'wall', 'strength', 'classic', 'buphi', 'ithink', 'khomeini', 'readi', 'wherea', 'whatsoev', 'lunat', 'preserv', 'carleton', 'section']

T2

['complaint', 'laboratori', 'arbor', 'wisdom', 'viewson', 'multisess', 'rebat', 'thatthey', 'sharp', 'preamp', 'iicx', 'impli', 'wood', 'eeee', 'avoid', 'suck', 'potenti', 'marc', 'attent', 'greet', 'guid', 'finish', 'carlo', 'eject', 'jose', 'pair', 'connor', 'frequent', 'bgrubb', 'kinda', 'regardless', 'revers', 'guarante', 'serv', 'presenc', 'informatik', 'sydney', 'sullivan', 'choos', 'rotat', 'saiid', 'toward', 'mint', 'summar', 'loos', 'width', 'known', 'ignor', 'initi', 'minimum']

T3

['brownbladerunnersugarcubeselectronicblaylockpowersspikel', 'wwizbhj', 'rlhz', 'wwizw', 'niet', 'cologn', 'pchz', 'siunivers', 'heikki', 'sdsu', 'cscx', 'respons', 'boucher', 'dkril', 'raider', 'zrlk', 'xrastool', 'bore', 'ucsc', 'vclk', 'iqvf', 'wocbn', 'magicangl', 'halici', 'gyuc', 'norm', 'static', 'derek', 'jrlk', 'onderzoek', 'proverb', 'adapt', 'smyx', 'kali', 'coventri', 'window', 'bizghj', 'lavrencicj', 'mbxlt', 'rqqd', 'meer', 'whensomeon', 'share', 'articl', 'tohc', 'gizd', 'scand', 'zghj', 'bnkjz', 'scub']

T4

['circumcis', 'kenneth', 'okay', 'balsamo', 'lincoln', 'bone', 'fatti', 'hail', 'impair', 'lugnut', 'anymor', 'tach', 'altitud', 'glove', 'elain', 'scam', 'punch', 'lactobacillus', 'favor', 'cabot', 'buyer', 'weird', 'ought', 'exceed', 'clue', 'kissan', 'grind', 'soft', 'refus', 'mega', 'cite', 'tough', 'acura', 'characterist', 'ident', 'graham', 'shaz', 'thanx', 'fictionin', 'argu', 'alic', 'numb', 'kinda', 'clutchless', 'gone', 'donald', 'appl', 'phenylalanin', 'bleed', 'kiran']

T5

['glass', 'heap', 'mayor', 'rocket', 'mission', 'cabin', 'hairenik', 'deliber', 'akgun', 'patriarch', 'defeat', 'pure', 'captain', 'shelter', 'fifti', 'jimmi', 'foundat', 'macedonia', 'assembl', 'outrag', 'erevan', 'telephon', 'vehicl', 'eggertj', 'troubl', 'beirut', 'herzl', 'amir', 'yeah', 'census', 'valuabl', 'grandfath', 'pano', 'terrifi', 'forward', 'scale', 'guardian', 'stomach', 'wast', 'fallen', 'closer', 'theisra', 'mouth', 'jump', 'track', 'network', 'intimid', 'bobb', 'disappear', 'leagu']

T6

['summer', 'optic', 'tobuild', 'dryden', 'mini', 'iiin', 'televis', 'circul', 'broderick', 'usingth', 'meemploy', 'billionin', 'launchpad', 'landi', 'port', 'ngai', 'compaq', 'strict', 'defray', 'cabin', 'gave', 'whatev', 'imaginit', 'ifit', 'kitchen', 'tout', 'brilliant', 'myself', 'conduct', 'leandro', 'bale', 'reader', 'defens', 'parachut', 'joist', 'cancel', 'hotwel', 'rack', 'triumph', 'henc', 'judg', 'nttcom', 'yougiv', 'sensor', 'packet', 'merchant', 'hanshin', 'extinguish', 'hafeez', 'doppelgang']

T7

['enrout', 'tonga', 'topex', 'educ', 'fisher', 'spread', 'ottolini', 'kaynaklari', 'ventura', 'parallel', 'evolv', 'okay', 'mount', 'apryan', 'stewart', 'drought', 'among', 'worth', 'seti', 'drake', 'dive', 'stream', 'academi', 'imperi', 'morgan', 'norad', 'hart', 'proper', 'vicin', 'astrophi', 'nickh', 'halo', 'landsat', 'axial', 'tether', 'rapid', 'stsci', 'macmillan', 'ingress', 'pacemak', 'lifetim', 'peri', 'gave', 'difficulti', 'modern', 'credibl', 'noaa', 'carrier', 'belgeleriyl', 'shaw']

T8

['ehrlich', 'behold', 'frameof', 'roommat', 'itinvolv', 'camco', 'ccreegan', 'stalnak', 'morkuna', 'abbrevi', 'gmill', 'magus', 'jdnicol', 'album', 'thumper', 'maestro', 'doubt', 'realidad', 'christen', 'deedon', 'burn', 'medg', 'anywher', 'hubert', 'seeeeee', 'room', 'hpscit', 'arrevola', 'tranquil', 'upsilon', 'canterburi', 'presumpt', 'muir', 'kant', 'deus', 'static', 'foreign', 'reed', 'muscl', 'buzz', 'cunixf', 'frashokereti', 'sever', 'goddam', 'blush', 'pivarnik', 'mecca', 'davison', 'quid', 'reitmanhow']

T9

['seventh', 'gwynn', 'harkey', 'nordiqu', 'build', 'stretcher', 'yell', 'kingoz', 'context', 'fifteen', 'wouldnot', 'leius', 'geoff', 'bomber', 'wish', 'surf', 'walter', 'cesar', 'velard', 'phillip', 'stronger', 'statement', 'lion', 'arbitr', 'jplee', 'geronimo', 'vineland', 'degan', 'gross', 'circl', 'steinman', 'inde', 'sylvain', 'syste

m', 'hanson', 'bone', 'junior', 'south', 'alex', 'push', 'forgot', 'omar', 'barber', 'piazza', 'sutter', 'collect', 'kean', 'calcul', 'santiago', 'shame']

T10

['notat', 'vitiello', 'status', 'subset', 'plenti', 'withth', 'libxaw', 'wiseb', 'lauderdal', 'meunier', 'decis', 'cmdline', 'aboutx', 'thoma', 'sheaffer', 'discret', 'tuchman', 'prestonm', 'inspect', 'operand', 'asctim', 'unlik', 'uuprog', 'usenix', 'passcdebugflag', 'pmview', 'interact', 'fantasi', 'theorigin', 'convexpolygon', 'areavail', 'hamlab', 'interv', 'whetherth', 'thesmal', 'barnsley', 'pound', 'none', 'anon', 'initialis', 'alford', 'siltanen', 'offx', 'inou', 'recov', 'comxanon', 'modul', 'textbook', 'hundr', 'fileif']

T11

['infinit', 'mitch', 'landsat', 'wiley', 'ncsu', 'intro', 'toronto', 'europ', 'magnet', 'matt', 'pari', 'craig', 'foley', 'instanc', 'minut', 'pixmap', 'semi', 'reprint', 'xloadimag', 'athen', 'rout', 'riordan', 'richmond', 'consider', 'lowel', 'gadget', 'decnet', 'variat', 'lack', 'seminar', 'properti', 'extra', 'mapl', 'kingdom', 'scroll', 'hello', 'reduct', 'japanes', 'latex', 'iowa', 'minimum', 'overview', 'cose', 'xmdrawingarea', 'compliant', 'london', 'svga', 'technion', 'zero', 'graphlib']

T12

['govern', 'enough', 'pharmaceut', 'tail', 'guidanc', 'televis', 'necessari', 'austria', 'spot', 'netherland', 'vari', 'demon', 'janus', 'exempt', 'ignor', 'familiar', 'assoc', 'oncologist', 'slaughter', 'perman', 'alchemi', 'applelink', 'fake', 'construct', 'print', 'internship', 'permiss', 'tenth', 'land', 'healthinsur', 'fuse', 'sixth', 'rank', 'fewer', 'stori', 'institution', 'anxieti', 'retina', 'motiv', 'geometri', 'baltimor', 'medlin', 'degener', 'scott', 'sunday', 'gross', 'klaus', 'uniform', 'generalmal', 'republiqu']

T13

['xresourc', 'outer', 'issu', 'mkfontdir', 'directcolor', 'ramakrishnan', 'elbel', 'search', 'former', 'sens', 'raymond', 'node', 'menchett', 'uniform', 'virtualbindingx', 'resolv', 'summar', 'abandon', 'clara', 'caveat', 'athena', 'singh', 'epfl', 'liddelow', 'thatth', 'tcsh', 'circular', 'autotrac', 'tester', 'repres', 'column', 'lusardi', 'libxext', 'morley', 'cardin', 'eventu', 'scan', 'dsto', 'dsysv', 'backgroundpixmap', 'correspond', 'dspptr', 'compar', 'xtaddeventhandl', 'sigpip', 'lookup', 'wide', 'tobi', 'acsc', 'kaleb']

T14

['ulkyvx', 'everytim', 'unca', 'suit', 'surpris', 'timbol', 'beast', 'netter', 'techniqu', 'nilay', 'walford', 'axion', 'workshift', 'iubac', 'solari', 'wise', 'monster', 'hijaak', 'hein', 'montana', 'grabber', 'umassd', 'borg', 'mycal', 'belong', 'versionof', 'ulowel', 'blackman', 'height', 'assur', 'withth', 'weird', 'aerg', 'meyer', 'muchado', 'danni', 'geni', 'microscopi', 'unsw', 'compon', 'uicvm', 'pope', 'eecg', 'cfmu', 'victor', 'damn', 'forum', 'interrupt', 'uninstal', 'desir']

Top words of topics obtained with hPAM model on the second hierarchical level for 20 Newsgroups dataset (pre-processing 2), $\eta = 0.2$.

T1

['enciph', 'think', 'axwm', 'fulfil', 'wrapper', 'bizwt', 'toth', 'terror', 'imagin', 'potenti', 'formula', 'tool', 'compon', 'discoveri', 'filler', 'rwing', 'alic', 'addr', 'adopt', 'sandman', 'slightest', 'ghjn', 'remedi', 'havel', 'checksum', 'czar', 'weitzner', 'thesystem', 'obscur', 'cxwm', 'scanner', 'layer', 'botz', 'myrto', 'levi', 'readi', 'axgiz', 'unclassifi', 'znkj', 'overs', 'isdn', 'flaw', 'torranc', 'toxicwast', 'bamford', 'barrus', 'ofencrypt', 'theconv', 'news', 'reverend']

T2

['plain', 'stick', 'jude', 'bgsu', 'saythat', 'redeem', 'nasb', 'teeth', 'essenti', 'sanctifi', 'yourselv', 'herring', 'restor', 'reproduc', 'fatima', 'hypocrit', 'ephesian', 'realm', 'theme', 'whatsoev', 'vatican', 'donot', 'gatech', 'masonri', 'anti', 'regardless', 'char', 'jeremiah', 'label', 'irrat', 'geno', 'imposs', 'abomin', 'denial', 'literari', 'nextwork', 'sing', 'virtual', 'redempt', 'crossroad', 'uunet', 'thecathol', 'ingl', 'messian', 'manuscript', 'eastern', 'isth', 'tortur', 'paraphra', 'nation']

T3

['vanhorn', 'epidem', 'whohav', 'tosometh', 'brand', 'accord', 'gage', 'gontier', 'younger', 'nathan', 'karl', 'abdomen', 'size', 'steam', 'gasin', 'watch', 'bimac', 'cern', 'allegra', 'enterpri', 'morphin', 'pelliccio

', 'augment', 'magick', 'rebutt', 'five', 'pathet', 'loren', 'andr', 'clamen', 'catch', 'ruegg', 'assembl', 'skull', 'emmisionsfrom', 'birth', 'cent', 'justcal', 'wont', 'china', 'telecom', 'minski', 'nail', 'gtefsd', 'hine', 'cuni', 'winter', 'italian', 'portland', 'karma']

T4

['stoop', 'thine', 'vernon', 'thus', 'cant', 'datsun', 'rebuilt', 'favorit', 'illeg', 'panic', 'sedat', 'comuucp', 'bowman', 'hang', 'matmcinn', 'myfriend', 'puzzl', 'temporari', 'voodoo', 'weekend', 'broke', 'norman', 'skcla', 'karl', 'horizont', 'arlington', 'checker', 'nuet', 'hhtra', 'honk', 'liggett', 'bianchi', 'trail', 'travi', 'thoma', 'slam', 'dduff', 'andrei', 'bowmanj', 'superior', 'impair', 'ownership', 'interv', 'ripoff', 'eight', 'duff', 'compet', 'crazi', 'poster', 'defrost']

T5

['applicationshellwidgetclass', 'hewlett', 'disabl', 'minster', 'xtaddcallback', 'bindir', 'xclrs', 'xtsetvalu', 'christi', 'construct', 'bockamp', 'wnkretz', 'mterm', 'stupid', 'hash', 'goudswaard', 'deskset', 'tend', 'popular', 'concurr', 'msdos', 'austria', 'bourn', 'xtappmainloop', 'wynap', 'kbyte', 'hardcod', 'energietechnik', 'adjust', 'mario', 'plymouth', 'dxterm', 'walter', 'extend', 'haywood', 'station', 'averag', 'prove', 'toml', 'writer', 'psize', 'trystero', 'consist', 'defaultscreen', 'isbn', 'bstatic', 'greet', 'bore', 'collect', 'quark']

T6

['poland', 'sloan', 'rental', 'hogan', 'saga', 'cunningham', 'hagen', 'upto', 'challeng', 'land', 'magic', 'breed', 'sicha', 'servicein', 'lupica', 'conduct', 'gannon', 'scandinavia', 'atlas', 'hold', 'loath', 'further', 'seam', 'broadway', 'jami', 'arlut', 'ucsd', 'agent', 'messina', 'issueof', 'kozierok', 'withdrawn', 'helsinki', 'dale', 'princ', 'multi', 'rail', 'tanner', 'circuit', 'banquet', 'swap', 'extend', 'geoffrey', 'berg', 'husc', 'legion', 'reynold', 'triumph', 'blackshaw', 'told']

T7

['wang', 'lossi', 'american', 'miscellan', 'mask', 'guidelin', 'mission', 'trentu', 'evan', 'morpholog', 'springer', 'grid', 'primari', 'zhenghao', 'nearest', 'figur', 'pixar', 'suffici', 'forg', 'columbia', 'macbinari', 'piec', 'neron', 'ntsc', 'promot', 'stand', 'contin', 'nssdca', 'hint', 'cracker', 'adverti', 'reduct', 'stop', 'recogn', 'hundr', 'shell', 'foley', 'self', 'fine', 'austin', 'cdrom', 'condit', 'companion', 'mind', 'comprehen', 'success', 'infinit', 'devot', 'carpent', 'spacelink']

T8

['wichitak', 'seat', 'forjust', 'infest', 'grab', 'analyz', 'kick', 'mistak', 'synchron', 'former', 'larger', 'initi', 'linnig', 'replic', 'eventu', 'infra', 'avenu', 'seek', 'bath', 'auth', 'knob', 'inclus', 'toaster', 'cope', 'glow', 'rage', 'addr', 'burt', 'stand', 'suscept', 'fiber', 'contrib', 'extend', 'fiberglass', 'universitaet', 'invent', 'stat', 'ihav', 'hatch', 'neededi', 'telecommun', 'andth', 'shar', 'hawkin', 'although', 'neat', 'tran', 'transfer', 'accord', 'cola']

T9

['grief', 'homeopathi', 'lend', 'wherebi', 'disneyland', 'oftheir', 'cooper', 'wouldb', 'sole', 'butthey', 'toparticip', 'tobr', 'innat', 'turmoil', 'peculiar', 'extrem', 'theoriz', 'goykhman', 'walnut', 'theamerican', 'insect', 'interceptor', 'dogmat', 'privat', 'confederaci', 'char', 'uclink', 'wasnot', 'calculus', 'charter', 'misrepr', 'gritti', 'whatexact', 'youin', 'sadden', 'cfaehl', 'brat', 'foryou', 'thatwhil', 'gulf', 'dialysi', 'segreg', 'hplabsz', 'tobeliev', 'baseb', 'entri', 'process', 'morrison', 'divin', 'multi']

T10

['edik', 'analog', 'less', 'somwher', 'forcibl', 'raid', 'kerem', 'progress', 'anticip', 'unten', 'mislead', 'precinct', 'link', 'descend', 'mose', 'smoke', 'victori', 'stole', 'iceland', 'mayor', 'circl', 'levi', 'hotel', 'belgeleriyl', 'thee', 'patriarch', 'brenda', 'complic', 'pleiad', 'terrifi', 'identif', 'quotat', 'danni', 'shop', 'faint', 'maler', 'outrag', 'paid', 'grandfath', 'persia', 'dirt', 'enforc', 'alcor', 'adolf', 'bath', 'forti', 'reaction', 'diplomaci', 'kristina', 'theholocaust']

T11

['flack', 'iceland', 'nothingbut', 'burnt', 'daughter', 'therear', 'evenif', 'flick', 'taketh', 'reckon', 'rode', 'ofsev', 'cherish', 'commonplac', 'obey', 'cartwright', 'ursa', 'iscal', 'sigh', 'ryan', 'egypt', 'ineffici', 'butt', 'pronounc', 'rosi', 'anxious', 'lowli', 'skill', 'buddha', 'appoint', 'quak', 'homeopath', 'streamlin', 'ofoth', 'messeng', 'comeclo', 'ridg', 'hail', 'monkey', 'clueless', 'whatth', 'unveil', 'aberr', 'thanksto', 'abound', 'tocontrol', 'jacket', 'depict', 'norwegian', 'foremost']

T12
['anaerob', 'infantil', 'request', 'poll', 'twice', 'tulsa', 'insect', 'lauren', 'maintain', 'exchang', 'spite', 'u ndisput', 'final', 'undocu', 'disproport', 'overbloom', 'micro', 'tast', 'earth', 'halcyon', 'lifetim', 'hill', ' push', 'pattern', 'volunt', 'stroke', 'evolutionari', 'bleed', 'channel', 'ofpeopl', 'imag', 'rubenfeld', 'sno rt', 'manual', 'oxygen', 'disparag', 'ensur', 'advantag', 'ginzberg', 'blast', 'bethesda', 'generalm', 'antig en', 'commerci', 'feder', 'feed', 'chairmandepart', 'antidiscrimin', 'divid', 'futur']
T13
['steven', 'expand', 'deer', 'irrelev', 'appli', 'madden', 'spent', 'finsh', 'jayson', 'yourself', 'baylor', 'asp ect', 'honest', 'corner', 'noke', 'vizcaino', 'medraut', 'carlo', 'levi', 'frustrat', 'nysernet', 'hojo', 'kahn', ' yale', 'popular', 'theteam', 'oppon', 'silvestri', 'calcul', 'threat', 'septemb', 'smiley', 'eject', 'carina', 'su rround', 'omar', 'polici', 'glad', 'repli', 'bose', 'famein', 'attitud', 'ulkyvx', 'meemploy', 'louisvil', 'sake ', 'curran', 'hidden', 'lanc', 'former']
T14
['projector', 'bosco', 'method', 'interleav', 'viper', 'arriv', 'death', 'inten', 'apart', 'technician', 'stock', ' localtalk', 'finder', 'excus', 'assur', 'guykuo', 'trust', 'bishop', 'mcmain', 'face', 'flaki', 'circuitri', 'aven u', 'suffici', 'middl', 'meant', 'mirror', 'chad', 'jartsu', 'salesperson', 'yourself', 'frode', 'watch', 'would b', 'micron', 'ritvax', 'soul', 'jester', 'desk', 'snail', 'forc', 'nasa', 'learn', 'generic', 'oppo', 'writer', 'unu s', 'marvin', 'meet', 'phys']
